# Heterometallic Au(I)–Cu(I) Clusters: Luminescence Studies and ^1^O_2_ Production

**DOI:** 10.1021/acs.inorgchem.3c00046

**Published:** 2023-05-16

**Authors:** Guillermo Romo-Islas, Jas S. Ward, Kari Rissanen, Laura Rodríguez

**Affiliations:** † Departament de Química Inorgànica i Orgànica, Secció de Química Inorgànica, Universitat de Barcelona, Martí i Franquès 1-11, 08028 Barcelona, Spain; ‡ Institut de Nanociència i Nanotecnologia (IN2UB), Universitat de Barcelona, 08028 Barcelona, Spain; § Department of Chemistry, University of Jyvaskyla, P.O. Box 35, 40014 Jyvaskyla, Finland

## Abstract

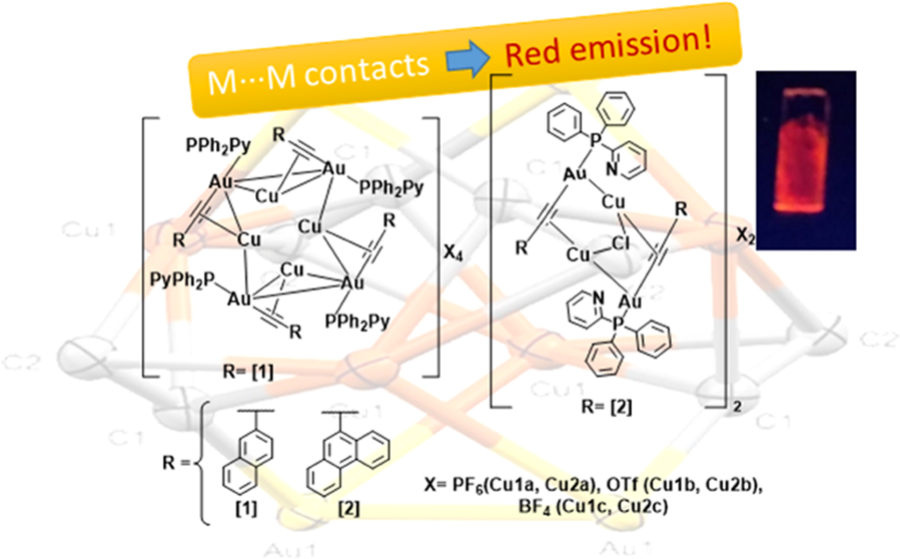

Two different organometallic gold(I) compounds containing naphthalene and phenanthrene as fluorophores and 2-pyridyldiphenylphosphane as the ancillary ligand were synthesized (compounds 1 with naphthalene and 2 with phenanthrene). They were reacted with three different copper(I) salts with different counterions (PF_6_^–^, OTf^–^, and BF_4_^–^; OTf = triflate) to obtain six Au(I)/Cu(I) heterometallic clusters (compounds **1a**–**c** for naphthalene derivatives and **2a**–**c** for phenanthrene derivatives). The heterometallic compounds present red pure room-temperature phosphorescence in both solution, the solid state, and air-equilibrated samples, as a difference with the dual emission recorded for the gold(I) precursors **1** and **2**. The presence of Au(I)–Cu(I) metallophilic contacts has been identified using single-crystal X-ray diffraction structure resolution of two of the compounds, which play a direct role in the resulting red-shifted emission with respect to the gold(I) homometallic precursors. Polystyrene (PS) and poly(methyl methacrylate) (PMMA) polymeric matrices were doped with our luminescent compounds, and the resulting changes in their emissive properties were analyzed and compared with those previously recorded in the solution and the solid state. All complexes were tested to analyze their ability to produce ^1^O_2_ and present very good values of Φ_Δ_ up to 50%.

## Abstract 2

The use of heterometallic Au/Cu systems containing a large number of metallophilic contacts is an ideal platform for obtaining red phosphorescent materials. The compounds synthesized in this work present an ionic character due to the reaction of gold(I) complexes with several copper(I) salts. The counterion affects, in some cases, tuning the emission wavelength. All of the compounds have been tested as photosensitizers in the generation of singlet oxygen.

## Introduction

Polydentate ligands are one of the most employed methods to generate complexes with a large number of atoms in their structure.^1−4^ The chemical structure of these ligands can be modified to include different donor atoms, and following this strategy, a wide variety of metallic clusters, polymers, aggregates, and supramolecular structures have been reported in the literature.^5−8^ Among polydentate ligands, the use of pyridylphosphane seems an ideal choice for the construction of heterometallic structures derived from group 11 elements due to their tendency to coordinated Au(I) through the phosphorus atom and to Ag(I) or Cu(I) through nitrogen centers. The presence of additional ancillary ligands such as acetylide groups may also favor the coordination of Ag(I) or Cu(I), promoting the formation of complex heterometallic assemblies.^9−11^

Metallic centers with closed-shell d^10^ electronic configurations have a tendency to form metallophilic (metal···metal) interactions. These interactions play an important role in the luminescent properties of the compounds, giving as a result compounds with different applications, for example, as luminescent materials with different colors such as blue^12^ green,^13^ red,^14^ or white emissions,^15^ thermally activated delayed fluorescence (TADF) systems,^10,16−18^ or as sensors^19−21^ among others. These applications depend not only on the metallophilic contacts but also on their ligands, where polydentate ligands play an important role in facilitating metal···metal proximity. Among d^10^ metallic compounds, gold(I) complexes bearing alkynyl moieties bonded to polyaromatic fluorophores have been demonstrated to be highly emissive.^22−25^ The emission of these systems is often related to the presence of aurophilic interactions and the formation of aggregates with high nuclearity.^26−32^ These interactions modify the electronic structure of the systems favoring the appearance of cluster-centered (CC) and metal–metal-to-ligand charge-transfer (MMLCT) emissive transitions.^27,33^ Additionally, intramolecular metal···metal interactions can be promoted by using polydentate ligands.^29^ Different donor atoms in these ligands can coordinate to several metal centers, keeping them close to each other and establishing heterometallic metallophilic interactions. Thus, the inclusion of a second metal ion, such as Ag(I) or Cu(I), in Au(I) complexes with multidentate ligands may promote the formation of cluster structures.^34−36^ These structures generate a change in the emission spectra of compounds following diverse processes such as CC, MMLCT, or LMMCT caused by heterometallic interactions with the different metals in the cluster core.^37,38^ Population of the *T*_1_ triplet excited state is quite common in this type of complexes containing heavy atoms that are responsible for the resulting phosphorescence emission.

Compounds with populated triplet excited states can also be involved in singlet oxygen (^1^O_2_) production. This research area is attracting increasing attention over recent years since it plays a key role in a diverse array of processes such as wastewater treatment, blood sterilization, photocatalytic processes, and photodynamic therapy.^39^ As photosensitizers, many organic compounds were studied to produce singlet oxygen with high quantum yields (QY). Usually, these compounds are nanotubes,^40^ graphene derivatives,^41^ and diverse molecules with π-donors and π-acceptors in their structure to promote the charge transfer to the triplet state.^42−45^ In comparison with the organic compounds, photosensitizers containing metal atoms used as ^1^O_2_ sensitizers are less studied and are mainly focused on metalloporphyrin, metal–organic framework (MOF), and nanoparticles.^46−48^

To the best of our knowledge, there are very few reports in the literature regarding organometallic gold(I) complexes used as photosensitizers with yields from low–moderate to high (20–57%).^32,49−54^ Additionally, to the best of our knowledge, no Au(I)/Cu(I) heterometallic clusters have yet been explored in this field. We are convinced that they deserve to be analyzed in this field in order to find out if the increase of the heavy atom effect and the cooperative effect of two different metals can positively affect the resulting energy transfer with oxygen.

Taking all of this into consideration, in this work, we have synthesized two gold(I) complexes containing a P–N polydentate ligand and a polyaromatic chromophore (naphthalene or phenanthrene) coordinated to the metal atom through an alkynyl group. These compounds have been used as precursors to obtain heterometallic clusters by the coordination of Cu(I) in the chemical structure. As is known, both the N-atom in the phosphane ligand and the alkynyl moiety may coordinate with the copper(I) metal centers and provide ancillary support to bimetallic cluster formation, thanks to the additional expected coordination to the Au(I) atoms (Au(I)···Cu(I) weak contacts).^34,35,55−57^ The obtained heterometallic complexes have been studied as red phosphorescence emitters and singlet oxygen photosensitizers.

## Results and Discussion

### Synthesis and Characterization

**Scheme 1:**
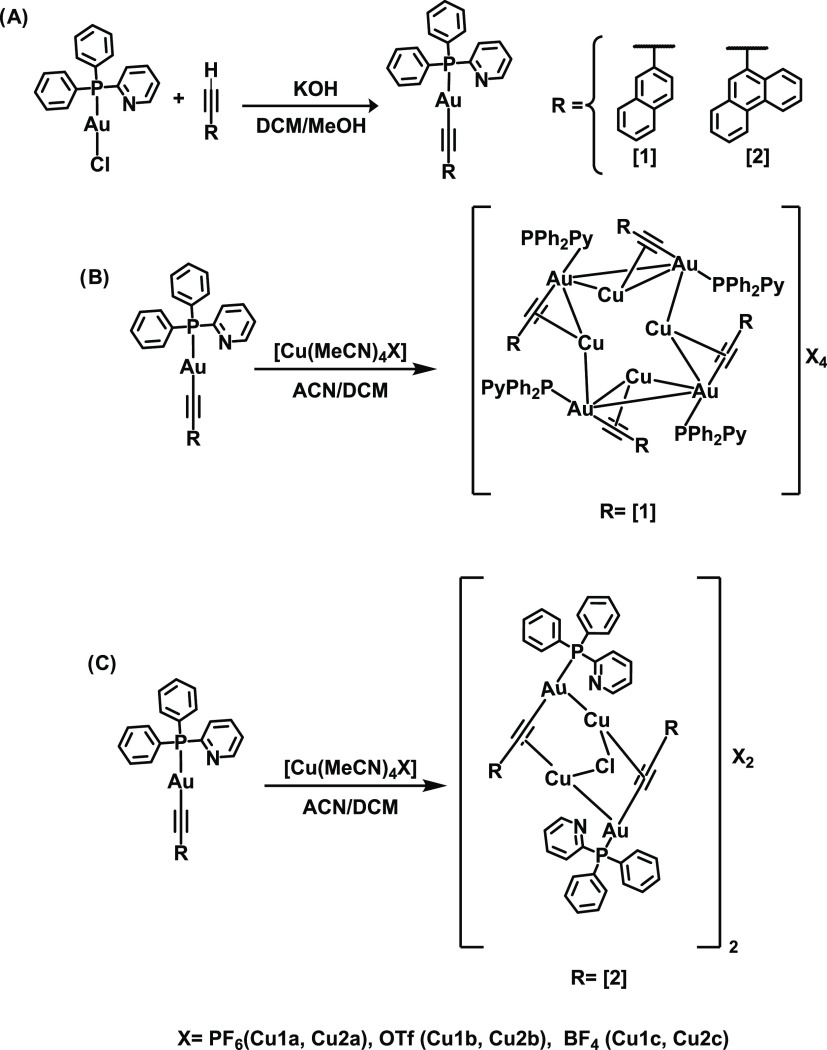
Synthesis of the Gold(I) Complexes **1** and **2** (A) and Their Heterometallic Cu(I)/Au(I) Derivatives (B, C)

The synthesis of the gold(I) complexes **1** and **2** has been carried out following a similar procedure previously used in our research group (Scheme 1A),^58−60^ that is, the deprotonation of the terminal alkynyl moiety and the subsequent reaction with the previously synthesized Au(pyPPh_2_)Cl^61^ compound.

Compounds **1** and **2** were reacted stoichiometrically with different copper(I) salts (PF_6_: **a**; OTf: **b**; BF_4_: **c**) in dichloromethane yielding six new heterometallic Au(I)–Cu(I) complexes (Scheme 1B). The compounds were obtained in pure forms after recrystallization with dichloromethane/hexane. Different heterometallic structures were obtained depending on the chromophore. In the case of the naphthalene derivatives, the resulting cluster consists of a symmetrical structure with a Au_4_Cu_4_ core (Scheme 1B), while a more open structure is generated in the case of the phenanthrene compounds that include chlorine bridging atoms connecting the Cu metal centers in the crystallization process (Scheme 1C). White powders were obtained for the gold(I) compounds, while orange-red solids were obtained for the heterometallic ones, all of them with moderate–high yields.

The ^1^H, ^19^F, and ^31^P NMR and IR spectroscopies together with electrospray ionization mass spectrometry (ESI-MS) (+) and (−) mass spectrometry demonstrate in all cases the correct formation of the desired compounds. The ^1^H NMR spectra of **1** and **2** (see Figures S1 and S3, respectively, in the Supporting Information (SI)) display the disappearance of the signal at 3.18 and 3.48 ppm, respectively, which corresponds to the terminal alkynyl protons and indicates the coordination of the gold(I) atom. The [M + H]^+^ peaks recorded in both cases in the corresponding ESI-MS(+) spectra verify the correct formation of the desired products (Figures S5 and S6 in the SI).

The ^1^H NMR spectra display that the protons in the ortho position to the nitrogen atom in the pyridyl ring of phosphane are affected by the coordination of the Cu(I) center in the case of **1a**–**c**, mainly for **Cu1a** and **Cu1b** with a 0.3–0.5 ppm downfield shift in comparison with **1** (Figure S31).^19^ This effect is even more pronounced for the series of complexes **2** and **Cu2a**–**Cu2c**, with a 0.5–0.6 ppm downfield shift of this proton (Figure S32). We can also observe that both naphthalene and phenanthrene protons become broader and upfield-shifted upon copper(I) coordination, probably due to the repolarization of the bond product of SOC influenced for the cluster formation^62,63^ or the rigidity of the molecule as a result of cluster formation.^55^ In fact, the Cu(I) cation can be bonded both to the pyridine moiety and through the alkynyl group. These interactions may exist with both intermolecular and intramolecular characters. Indeed, the first evidence of the presence of this kind of interaction in the solid state was given by the IR spectra of the compounds. It was observed that the ν(C≡C) vibration of **1** and **2** (observed at 2111 and 2112 cm^–1^, respectively) was shifted to 2005–2077 cm^–1^ indicating a slight decrease in the bond order by the weak coordination to the second metal center.

**Figure 1:**
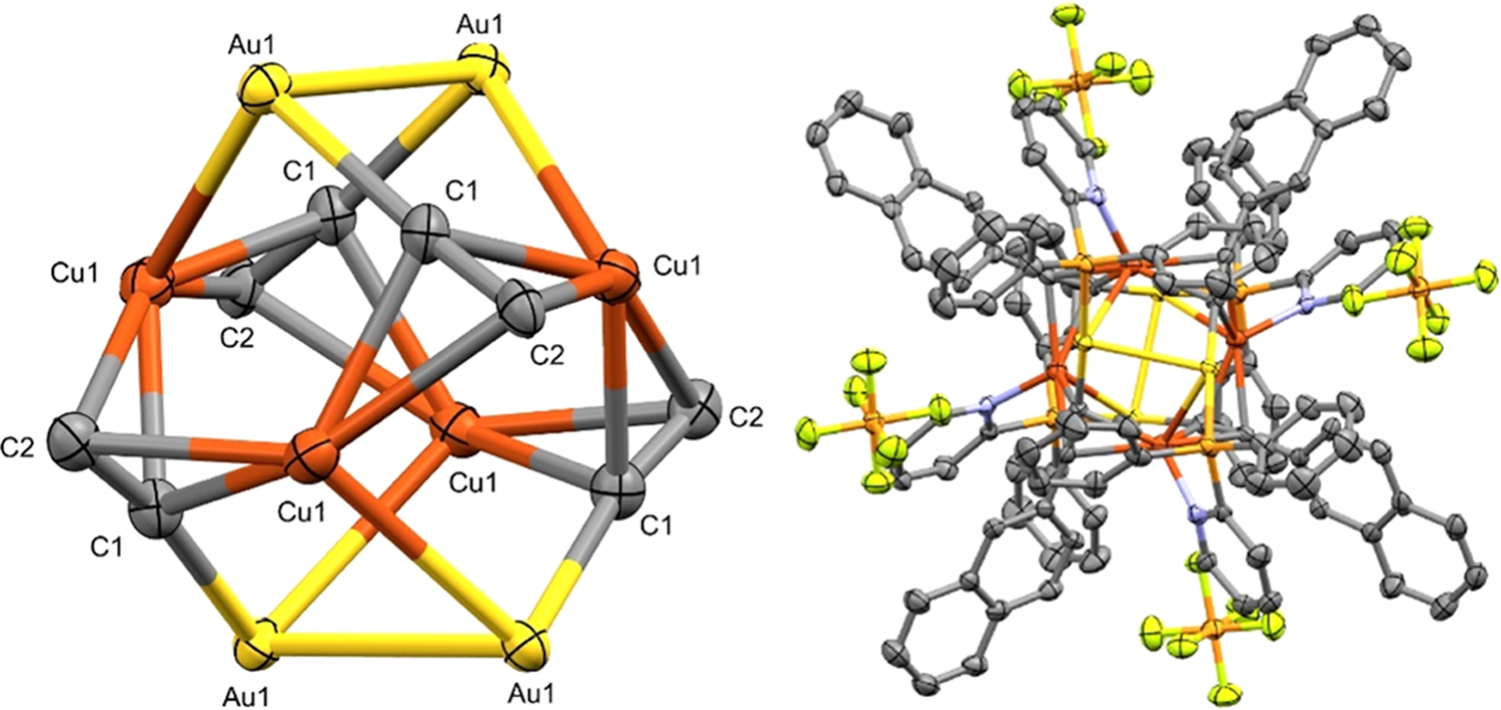
Left: cluster core on **Cu1a** on the axis *c*. Right: structure of complex **Cu1a** on the axis *b*. Atom colors: yellow, gold; bronze, copper; orange, phosphorus; light green, fluorine (thermal parameters at a 50% probability; hydrogen atoms have been omitted for clarity).

The heterometallic Au(I)/Cu(I) complexes present better solubility in common organic solvents and permit the growth, in some cases, of single crystals suitable for X-ray diffraction to determine the structure and coordination motifs of the products. Single crystals of **Cu1a** were grown by slow evaporation of a dichloromethane solution of the compound. The structure of **Cu1a** shows a cluster core containing four Au(I) metal centers and four Cu(I) metal centers in the unit [AuCu(PyPPh_2_)(C≡CC_10_H_7_)]_4_ with two different distances of the Cu(I) to the centroid generated by the two C≡C moieties (*d*_Cu–C≡C(centroid)_ = 2.099(5) and 2.153(5) Å) and a Cu···Cu distance of 3.297 Å. The angles around the Cu centers correspond to N–Cu–centroid(C≡C) of 102.1(2)° and Au–Cu–centroid(C≡C) of 95.0(2)°. For the Au(I) centers, the *d*_Au–P_ = 2.286(2) Å, *d*_Au–C_ = 2.029(6) Å, the angle P–Au–C = 176.4(2)°, P–Au–Cu = 82.40(5)°, and C–Au–Cu = 97.8(2)°. The Au(I)–Cu(I) heterometallic interaction in the cluster presents a *d*_Au–Cu_ = 2.7197(9) Å (Figure 1).

The crystal packing of the compound presents the individual clusters producing empty inner cavities that are occupied by the PF_6_^–^ counterion (Figure S33).

**Figure 2:**
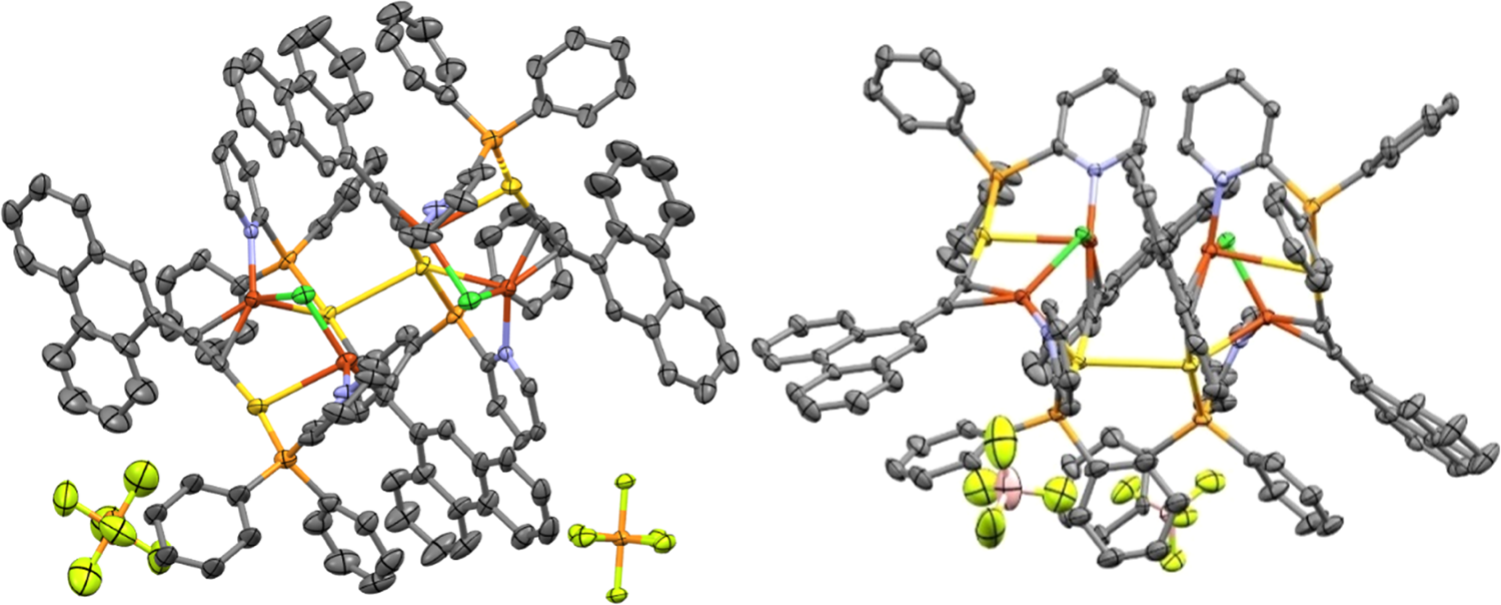
Structure of the cluster core of complexes **Cu2a** (left) and **Cu2c** (right) on the axis *c*. (Atom colors: yellow = gold, bronze = copper, orange = phosphorus, light green = fluorine, green = chlorine, pink = boron; thermal ellipsoids at a 50% probability; hydrogen atoms and solvate molecules have been omitted for clarity).

Red crystals of **Cu2a** and **Cu2c** were grown by the evaporation of the sample in chloroform, and the corresponding cluster core structures are presented in Figure 2. The asymmetric unit cell of **Cu2a** shows a cluster structure with two counteranions. We observe that, in both cases, these clusters derived from phenanthrene present a more open conformation in comparison with the analogous naphthalene **Cu1a**, which could be ascribed to the higher volume of the chromophore and to the inclusion of two chlorine atoms (that may come from the formation of some HCl traces from the solvent of crystallization during the crystal growth) bonded to the Cu(I) atoms. As observed for **Cu2a**, this compound presents two different distances to the centroid generated by the two C≡C moieties with a *d*_Cu–C≡C(centroid)_ = 2.036(6) and 2.042(6) Å (2.053(7) and 2.013(7) Å, respectively, for **Cu2c**), which are shorter than in the naphthalene analogous and a Cu···Cu distance of 3.543 Å. The angles for N–Cu–centroids(C≡C) of **Cu2a** are between 127.98(3)–132.44(3)°, which are similar to the **Cu2c** complex with 126.46(2)–133.83(2)° for equivalent atom connectivity; this is caused by the inclusion of chlorine atoms in the metallic clusters that open the metallic shell being the angles Cu–Cl–Cu = 95.55(7)° (101.51(7)° for **Cu2c**), Au–Cu–centroid(C≡C) = 101.5(2)° (105.5(2)° for **Cu2c**) and Cl–Cu–centroid(C≡C) = 140.3(2)° (142.1(2)° for **Cu2c**). The main parameters around the Au(I) centers are *d*_Au–P_ = 2.280(2) and 2.253(2) Å, *d*_Au–C_ = 2.012(6) and 2.011(7) Å, and the angles P–Au–C = 176.7(2) and 171.1(2)°, P–Au–Cu = 80.71(5) and 81.35(5)° and C–Au–Cu = 97.0(2) and 99.8(2)° for **Cu2a** and **Cu2c**, respectively. The Au(I)/Cu(I) heterometallic interactions present a distance *d*_Au–Cu_ = 2.828(1) and 2.810(1) Å for **Cu2a** and **Cu2c**, slightly larger than in the naphthalene analogous, probably due to the resulting more open structure (Table 1).

**Table 1:** Main Distances and Angles of Complexes **Cu1a**, **Cu2a**, and **Cu2c**

	distances (Å)					angles (deg)		
complex	Au1–Au1	Au1–Cu1	Cu1–N22	Cu1–C2	Au2–Au2	C1–Au1–P1	C1–Cu1–Au1	N22–Cu1–Au1
**Cu1a**	2.9875(7)	2.7197(9)	2.062(6)	2.167(5)	2.9700(7)	176.4(2)	88.2(2)	93.1(2)
**Cu2a**	3.0187(6)	2.790(1)	2.007(6)	2.033(7)		176.7(2)	97.0(2)	89.5(2)
**Cu2c**	2.9940(5)	2.810(1)	2.026(5)	2.079(7)		171.9(2)	105.5(2)	93.3(1)

#### Photophysical Properties

The absorption and emission spectra of the heterometallic complexes and their respective gold(I) precursors were measured in 10^–5^ M CH_2_Cl_2_ solutions at room temperature (Table 2 and Figures S34–S43). The absorption spectra of the compounds show vibronically structured bands at 315 and 333 nm for **1** and **2**, respectively, that are assigned to π–π* intraligand transitions.^64^ A new band centered at 355 nm appears for the heterometallic cluster complexes **Cu1a–Cu1c** and **Cu2a–Cu2c**. This band becomes broader and red-shifted in these complexes due to the presence of metallophilic interactions and the resulting cluster-centered (^3^CC) transitions (Figures S38–S43).^65^

**Table 2:** Photophysical Properties of Compounds **1** and **2** and Their Au(I)/Cu(I) Heterometallic Complexes in Solution^a^

complex	λ_max_ Abs, nm (ε × 10^3^ cm^–1^ m^–1^)	λ_max_ Em (nm)	Φ_fl_ (air-eq/N_2 sat_)	Φ_phos_ (air-eq/N_2 sat_)	τ (air-eq/N_2 sat_, μs)
**1**	315 (23.6)	356, 505, 545	0.002/0.003	0.001/0.019	0.004/0.005
**2**	315 (22.5)	384, 535, 579	0.004/0.005	0.002/0.014	0.005/0.006
**Cu1a**	315 (20.2), 333 (16.4), 355 (9.1)	642		0.02/0.05	0.28/0.29
**Cu1b**	315 (11.3), 333 (10.3), 355 (6.0)	643		0.02/0.06	0.32/0.34
**Cu1c**	315 (11.9), 333 (10.2), 355 (9.0)	643		0.01/0.02	0.10/0.12
**Cu2a**	315 (17.6),333 (20.9), 355 (19.9)	628		0.06/0.13	4.8/7.5
**Cu2b**	315 (21.2), 333 (24.8), 355 (23.3)	630		0.06/0.12	4.2/6.7
**Cu2c**	315 (11.8), 333 (12.9), 355 (11.0)	629		0.05/0.09	4.3/6.9

a Φ_Fl_ = fluorescence QY; Φ_Phos_ = phosphorescence QY. τ = lifetimes.

Fluorescence emission has been recorded for the gold(I) complexes in solution at room temperature with emission maxima at 356 and 384 nm for **1** and **2**, respectively. Dual emission has been recorded when the samples are deoxygenated with a phosphorescence band at 545 for **1** and 535 nm for **2** (Figures S38–S43). The vibronical resolution of both fluorescence and phosphorescence bands allows us to assign these transitions to metal-perturbed IL emissions (^1^IL and ^3^IL) located at the ethynylnapthalene or ethynylphenanthrene units. Phosphorescence emission is much more favored in the case of the naphthalene compounds.

**Figure 3:**
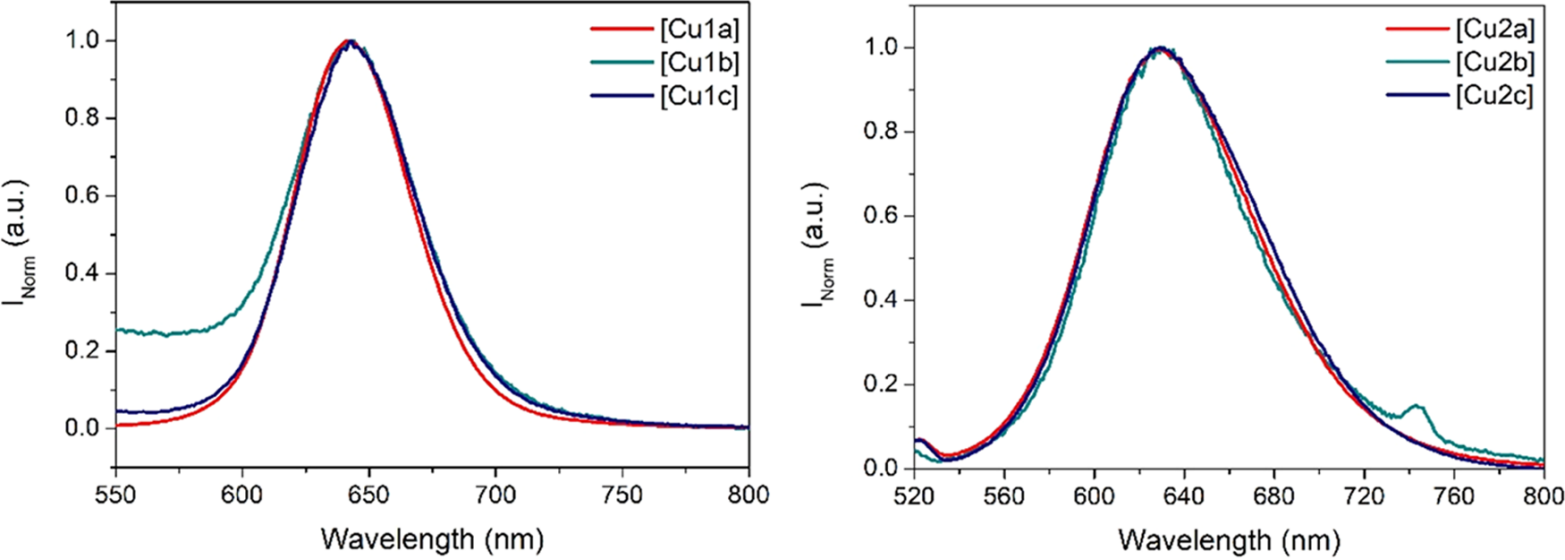
Normalized emission spectra of complexes **Cu1a**–**Cu1c** (left) and **Cu2a**–**Cu2c** (right) in 10^–5^ M CH_2_Cl_2_ solutions (λ_exc_ = 450 nm).

Interestingly, pure room-temperature phosphorescence has been observed for all heterometallic complexes with broad bands centered at *ca.* 642 nm for **Cu1a**–**Cu1c** and at *ca.* 630 nm for **Cu2a**–**Cu2c** (Figures 3, S38, and S41). This emission appears in air-equilibrated solutions and is not affected by the nature of the counteranion. The resulting emission is red-shifted with respect to the gold(I) homometallic compounds as previously observed with other Au/Cu heterometallic systems containing the same pyPPh_2_ phosphane previously reported in the literature. These transitions have been ascribed to an admixture of IL/LL′/LAuMCT transitions.^37,55,56,66−70^ In particular, the ligand-to-metal charge-transfer character of the transitions has been previously attributed thanks to time-dependent density-functional theory (TDDFT) calculations, and this charge-transfer process is observed to increase in the heterometallic compounds with respect to the homometallic, with the main role of the copper center.^55,56^ This would correlate with the observed resulting emission red shift.

Thus, we do not expect any type of interaction in solution between the metallic core and the counterions.^71^ The different properties between the gold(I) parent compounds and the heterometallic compounds support the successful formation and stability of the new Au(I)/Cu(I) heterometallic structures in solution. Unfortunately, we did not manage to detect the desired Au_4_Cu_4_ full core in the mass spectra. Nevertheless, the resulting room-temperature phosphorescence recorded for **Cu1a**–**Cu1c** and **Cu2a**–**Cu2c** strongly supports the formation of the new heterometallic systems, with completely different emission profiles with respect to their parent gold(I) homometallic complexes and, thus, the presence of new (heterometallic) structures in solution.

The recorded photoluminescence quantum yields (QY)s are larger for the phenanthrene heterometallic clusters with respect to the analogous containing naphthalene. In both cases, the triflate and hexafluorophosphate derivatives (**a** and **b** compounds) display QY values larger than the tetrafluoroborate compounds **c** in both air-equilibrated and N_2_-saturated solutions, with larger values (approximately double) in the absence of oxygen (Table 2). Lifetime measurements are around 0.1–0.3 μs for **Cu1a**–**Cu1c** and 5–8 μs for **Cu2a**–**Cu2c**; the heterometallic complexes are slightly affected by the presence of oxygen, being 2 orders of magnitude larger than the corresponding values measured for the gold(I) complexes that display a lifetime of nanoseconds.

The radiative (*k*_r_) and nonradiative constants (*k*_nr_) were calculated in all cases, and we can observe first that the very large *k*_nr_ values of compounds **1** and **2** are responsible for the lack of phosphorescence emission in air-equilibrated solutions and very weak in N_2_-saturated samples. On the other hand, again, the counterion is affecting only series **1** and not series **2** in both air-equilibrated and N_2_-saturated solutions. The nonradiative contributions are also more important in series **1** in agreement with their lower recorded quantum yields (Table 3).

**Table 3:** Radiative (*k*_r_) and Nonradiative (*k*_nr_) Constants of the Gold(I) Complexes **1** and **2** and Their Heterometallic Au(I)–Cu(I) Complexes in Solution Considering the Phosphorescence Emission

complex	*k*_r_ (air-eq) × 10^5^ s^–1^	*k*_nr_ (air-eq) × 10^5^ s^–1^	*k*_r_ (N_2 sat_) × 10^5^ s^–1^	*k*_nr_ (N_2 sat_) × 10^5^ s^–1^
**1**			40	1960
**2**			16.7	1680
**Cu1a**	0.7	34.5	1.8	33.6
**Cu1b**	0.6	31.1	1.8	27.5
**Cu1c**	1.0	102	1.7	81.7
**Cu2a**	0.1	2.0	0.1	1.2
**Cu2b**	0.1	2.2	0.2	1.3
**Cu2c**	0.1	2.2	0.2	1.3

**Figure 4:**
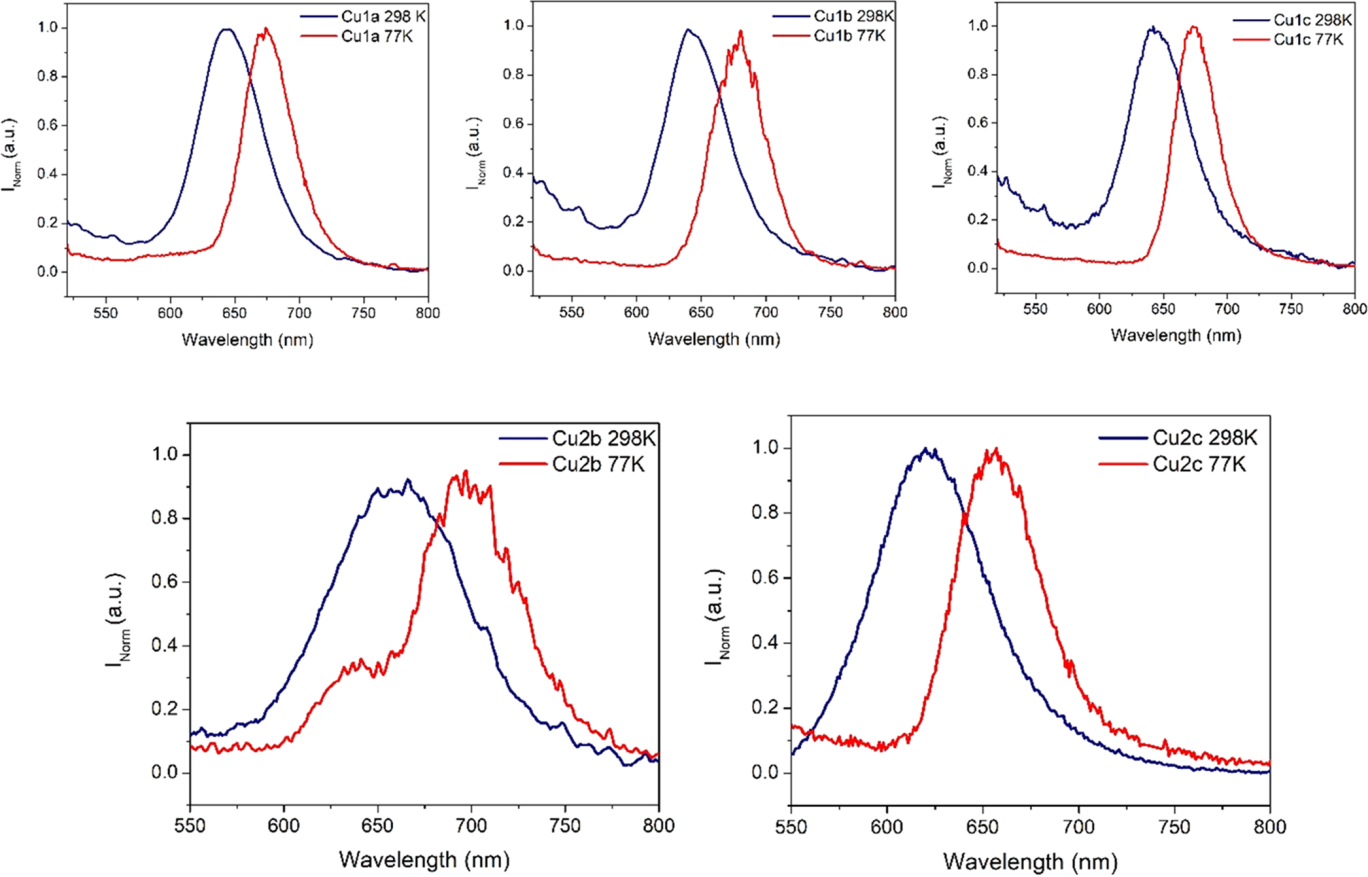
Normalized emission spectra of complexes **Cu1a**–**Cu1c** (above) and **Cu2b**–**Cu2c** (below) in the solid state at 298 and 77 K (λ_exc_ = 450 and 470 nm, respectively).

The emissions in the solid state for the six cluster complexes **Cu1a**–**Cu1c** and **Cu2a**–**Cu2c** were measured at 298 and 77 K (Table S1 in the SI and Figure 4). A red shift of the emission is recorded in almost all cases (except for **Cu2a**) when the powders are cooled (Figure 4). The observed red shift at low temperatures has been observed in other heterometallic Au(I)/Cu(I) complexes reported in the literature.^72−74^

The emission of all of the compounds has also been analyzed when the samples are immobilized within two different polymeric matrices (polystyrene, PS, and poly(methyl methacrylate), PMMA) using a low amount of the compounds (1%) as doping agents. Interestingly, and as a difference with what was previously observed in solution, room-temperature phosphorescence has been recorded as the main emission also for the gold(I) complexes **1** and **2**. The vibronically structured band allowed us to assign this phosphorescence to a metal-perturbed ^3^IL transition. As expected for a more rigid environment, the recorded QY in this media are larger than those previously recorded in solution (see Table 5).

**Figure 5:**
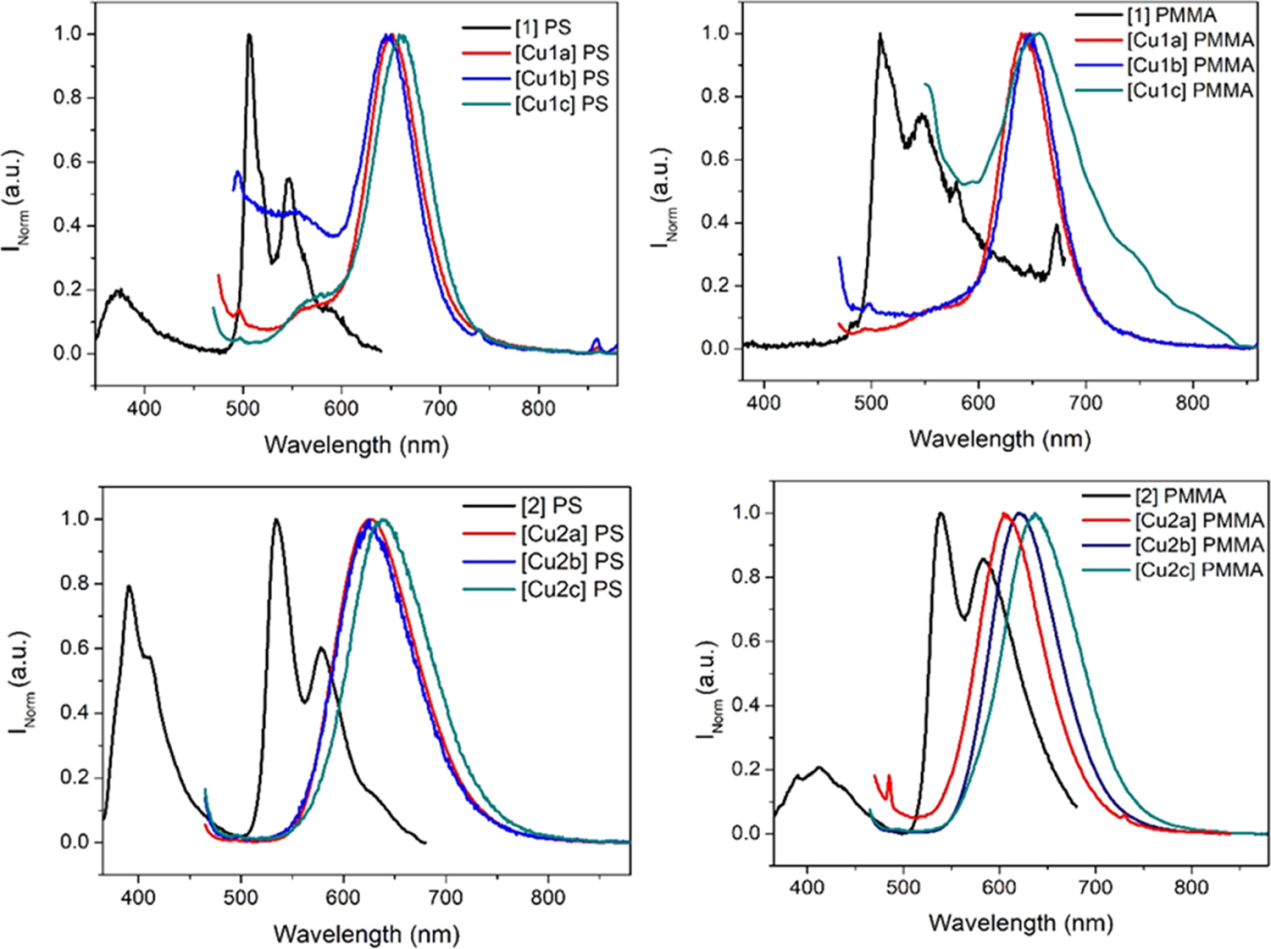
**Cu1a**–**c** (top) and **Cu2a**–**c** (bottom) complexes supported in polymeric solid matrices PS (left) and PMMA (right). λ_exc_ = 450 nm.

The heterometallic compounds do not display the same behavior in both series of complexes. **Cu1a**–**c** heterometallic compounds present a broad phosphorescence band at *ca.* 650 nm in both PS and PMMA (Figure 5). This band is closer to the one recorded in the solid state at 77 K.

On the other hand, the **Cu2a**–**c** compounds are more affected by the polymer, mainly in PMMA where a red shift of *ca*. 100 nm is observed in the order PF_6_^–^ < OTf^–^ < BF_4_^–^ (Figure 5). This can be rationalized by the fact that the less bulky counterion BF_4_^–^ can be more dispersed within the polymer, allowing larger interactions and dispersion of the heterometallic cluster in the matrix than in the case of larger counterions. On the contrary, there is no significant shift in the emission of the samples when they are immobilized in the more apolar PS polymer. All compounds present an enhancement of phosphorescence QY in PMMA in comparison with the PS matrix, in agreement with recent studies with alkynyl gold(I) complexes supported in these matrices (Table 4).^20,75^

**Table 4:** Quantum Yield (QY) Values (Φ) and Emission Lifetimes (τ) of Gold(I) Complexes **1** and **2** and Their Heterometallic Au(I)–Cu(I) Complexes in Solid State and Supported in Polymeric Matrices^a^

complex	Φ_FI/Phos_ (solid state)	Φ_Fl/Phos_ (PMMA)	Φ_Fl/Phos_ (PS)	τ_Fl/Phos_ (μs) solid state	τ_Fl/Phos_ (μs) PMMA	τ_Fl/Phos_ (μs) PS
**1**		0.002/0.046	0.002/0.010		1.1 × 10^–3^/0.166	7.2 × 10^–3^/0.006
**2**		0.006/0.030	0.009/0.010		2.2 × 10^–3^/0.056	6.1 × 10^–3^/0.012
**Cu1a**	–/0.04	–/0.04	–/0.01	–/2.15	–/0.137	–/0.093
**Cu1b**	–/0.01	–/0.03	–/0.01	–/0.05	–/0.121	–/0.089
**Cu1c**	–/0.01	–/0.02	–/0.01	–/1.15	–/0.071	–/0.056
**Cu2a**	–/0.11	–/0.15	–/0.08	–/1.65	–/14.65	–/5.00
**Cu2b**	–/0.07	–/0.09	–/0.05	–/1.04	–/15.34	–/7.38
**Cu2c**	–/0.05	–/0.08	–/0.03	–/0.77	–/15.36	–/5.98

a Φ_Fl_ = fluorescence QY; Φ_Phos_ = phosphorescence QY.

### Singlet Oxygen Production

The use of the two gold(I) complexes **1** and **2** and the six Au(I)–Cu(I) clusters as ^1^O_2_ photosensitizers has been tested since we wondered if the presence of such many heavy atoms in close proximity may have a direct influence on the population of the triplet excited state and the energy-transfer process to ^3^O_2_. These studies have been carried out by the direct measure of the characteristic ^1^O_2_ emission at 1270 nm, which corresponds to ^1^Δ_g_ → ^3^∑_g_^–^ transition upon excitation of the samples at λ_exc_ = 365–380 nm in air-equilibrated acetonitrile solutions and using perinaphthenone as the reference (Φ_Δ_ = 79%).

The results show that all phenanthrene derivatives behave slightly better as ^1^O_2_ photosensitizers than the analogous naphthalene derivatives (Table 5 and Figures S56–S63) with Φ_Δ_ ∼ 50 for series **2** and ∼40% for series **1**. The two BF_4_^–^ derivatives (**Cu1c** and **Cu2c**) display values slightly lower than the other complexes.

**Table 5:** ^1^O_2_ Quantum Yield (Φ_Δ_) Calculated for All Compounds in Acetonitrile Air-Equilibrated Solutions

compound	Φ_Δ_ (%)
**1**	40
**Cu1a**	36
**Cu1b**	37
**Cu1c**	25
**2**	50
**Cu2a**	42
**Cu2b**	47
**Cu2c**	38

Recent studies with alkynyl gold(I) derivatives show ^1^O_2_ production in the range of 9–29%.^53,54^ Thus, we managed to improve the singlet oxygen quantum yield values with our compounds although the expected heterometallic metallophilic interactions do not seem to positively affect the photosensitizing behavior, possibly due to the interference of the counterion (mainly the smaller BF_4_^–^) with the intermolecular contacts in solution or due to the different energy levels between the heterometallic and homometallic systems.

## Conclusions

The addition of Cu(I) salts to gold(I) complexes containing alkynylnaphthalene and alkynylphenanthrene as chromophores allowed us to obtain heterometallic cluster complexes with different emissive properties that differ mainly on the bulkiness of the chromophore. The spectra in the solution of clusters induce a red shift and a pure room phosphorescence emission in air-equilibrated samples in comparison with the Au(I) analogous complexes that only display phosphorescence in the absence of oxygen. The phosphorescence of the gold(I) compounds can also be induced when they are introduced as doping agents of polymeric organic matrices (PMMA and PS). The interaction with the matrix has a larger effect on the phenanthrene heterometallic compounds in PMMA. All compounds present denotable values of singlet oxygen quantum yields in comparison with the literature, offering a platform to improve the research on the use of organometallic complexes as singlet oxygen photosensitizers.

The formation of heterometallic clusters had a direct influence on the resulting phosphorescence and red emissions, though only a minimal effect was seen on the interaction with ^3^O_2_ toward their use as photosensitizers.

## Experimental Section

All air- and moisture-sensitive manipulations were carried out with standard Schlenk techniques under a nitrogen atmosphere. Solvents were purchased from commercial sources and dried by distillation under a nitrogen atmosphere. Reagents 2-ethynyl naphthalene, 9-ethynylphenanthrene, KOH, 2-(diphenylphosphino)pyridine (PyPh_2_P), [Cu(MeCN)_4_]PF_6_, [Cu(MeCN)_4_]OTf, and [Cu(MeCN)_4_]BF_4_ were purchased from commercial sources and used without further purification. [Au(tht)Cl],^76^ the phosphanegold(I) chloride precursor [PyPh_2_PAuCl],^61^ was prepared from the reaction of [Au(tht)Cl] with the phosphane PyPh_2_P.

Infrared spectra have been recorded on an FT-IR 520 Nicolet spectrophotometer. ^1^H NMR (δ(TMS) = 0.0 ppm) and ^31^P NMR (δ(85% H_3_PO_4_) = 0.0 ppm) spectra have been obtained on Bruker 400 and Bruker 500 (Universitat de Barcelona) instruments. Electrospray mass spectra (+) have been recorded on a Fisons VG Quatro spectrometer (Universitat de Barcelona). Absorption spectra were obtained in a 5 or 10 mm quartz cuvette in acetonitrile on a Varian Cary 100 Bio UV spectrophotometer. The emission spectra of the compounds in solution were obtained in a fluorescence quartz cuvette of 10 mm path length, using a Horiba-Jobin-Yvon SPEX Nanolog spectrofluorimeter, using slits of 2 nm. Low-temperature measurements were recorded using a Dewar adapted to the sample location of the spectrofluorometer. Quantum yields have been recorded on a Hamamatsu absolute PL quantum yield spectrometer C11347 upon excitation of the samples at 330 nm. Luminescence lifetimes were measured on a JYF-DELTAPRO-NL equipment upon excitation of the samples with a 390 nm NanoLED and collecting the decays through a bandpass filter of 550, 600, or 650 nm, depending on the emission maximum. The best fittings correspond to biexponential decays, and the indicated values correspond to the average considering the respective amplitudes.

The single-crystal X-ray data for **Cu1a** and **Cu2a** were collected at 120 K using an Agilent SuperNova diffractometer with an Eos detector using mirror-monochromated Mo Kα (λ = 0.71073 Å) radiation. The single-crystal X-ray data for **Cu2c** were collected at 120 K using an Agilent SuperNova dual wavelength diffractometer with an Atlas detector using mirror-monochromated Cu Kα (λ = 1.54184 Å) radiation. All structures were solved by intrinsic phasing (SHELXT)^77^ and refined by full-matrix least squares on *F*^2^ using Olex2,^78^ utilizing the SHELXL module.^79^ Anisotropic displacement parameters were assigned to non-H atoms, and isotropic displacement parameters for all H atoms were constrained to multiples of the equivalent displacement parameters of their parent atoms with *U*_iso_(*H*) = 1.2 *U*_eq_ of their respective parent atoms. The X-ray single-crystal data and CCDC numbers of all new structures are included in the Supporting Information.

### Spectroscopic Measurements

All measures were realized using spectroscopic quality solvents in 10^–5^ M concentration. For ^1^O_2_ measurements, the dichloromethane solutions were adjusted to an *A* = 1 in the peak of excitation wavelengths.

### Singlet Oxygen Quantum Yields

Room-temperature singlet oxygen emission was detected at 1270 nm with a Horiba-Jobin-Yvon SPEX Nanolog spectrofluorimeter (Universitat de Barcelona) using the DSS-IGA020L detector. The use of a Schott RG 1000 filter was essential to eliminate from the infrared signal all of the first harmonic contribution of sensitizer emission in the region below 850 nm. The singlet oxygen formation quantum yield was then determined by the direct measurement of the phosphorescence at 1270 nm following irradiation of the aerated solution in dichloromethane of the samples. Perinaphthenone in dichloromethane was used as a standard reference, applying eq 1
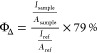
1

### Synthesis and Characterization

#### [(PyPh_2_P)AuC≡CNaph] (**1**)

KOH (17 mg, 0.303 mmol, 1.5 equiv) was added to a methanol (5 mL) solution under a N_2_ atmosphere. 2-Ethynyl-naphthalene (30.7 mg, 0.202 mmol, 1 equiv) was added. The mixture was stirred for 2 h at 25 °C. Then, a solution of [(PyPh_2_P)AuCl] (100 mg, 0.202 mmol, 1 equiv) in 3 mL of dichloromethane was slowly added. After 76 h, the solvents were removed under a vacuum. The solid was dissolved in 1 mL of dichloromethane and precipitated with 3 mL of hexane. **1** was obtained as a white solid (102 mg, 82%). ^1^H NMR: (dimethyl sulfoxide (DMSO)-*d*_6_ 400 MHz) δ = 8.85 (d, *J* = 4.7 Hz, 1H), 8.04–7.97 (m, 1H), 7.84 (ddd, *J* = 15.5, 12.1, 6.6 Hz, 4H), 7.77–7.71 (m, 1H), 7.69–7.57 (m, 10H), 7.53–7.45 (m, 2H), 7.42 (dd, *J* = 8.5, 1.6 Hz, 1H), 7.34 (dd, *J* = 8.5, 1.6 Hz, 1H) ppm; ^31^P NMR: (DMSO-*d*_6_ 162 MHz) δ = 41.29 ppm. ESI (*m*/*z*): 1071.15 [2M-Naph]^+^, 723.13 [Au(PyPhos)_2_]^+^, 612.11 [M + H]^+^, IR (cm^–1^): ν = 3039.50 (ar), 2111.25 (C≡C), 1590.85 (C=N), 1480.63 (C–H ar), 1100.98 (P–C).

#### [(PyPh_2_P)_4_Cu_4_Au_4_(C≡CNaph)_4_][PF_6_]_4_ (**Cu1a**)

In a sealed flask under a N_2_ atmosphere, **complex 1** (10 mg, 0.0178 mmol, 1 equiv) was added in 3 mL of CH_2_Cl_2_ in stirring for 5 min. Then, [Cu(MeCN)_4_]PF_6_ (6.09 mg, 0.0178 mmol, 1 equiv) dissolved in 2 mL of CH_2_Cl_2_ was added. After 3 h, the solution was reduced to dry under vacuum. The solid was dissolved in 1 mL of dichloromethane and precipitated with 2 mL of hexane. **Cu1a** was obtained as a red-orange solid (9.2 mg, 67%). ^1^H NMR: (CDCl_3_, 400 MHz) δ = 8.79 (d, *J* = 4.8 Hz, 1H), 8.00 (dd, *J* = 14.9, 7.4 Hz, 2H), 7.86–7.77 (m, 1H), 7.74–7.65 (m, 6H), 7.57–7.43 (m, 9H), 7.43–7.37 (m, 3H) ppm; ^19^F NMR: (CDCl_3_ 162 MHz) δ = −71.35 ppm (d, *J*_P–F_ 714.9 Hz, 6F), ^31^P NMR: (CDCl_3_ 162 MHz) δ = 33.6 (s), −141.5 (hept) ppm. ESI (*m*/*z*): 1897.25 [3M + Cu + H]^+^, 1383.04 [2M + 2Cu + Cl + H]^+^, 1285.14 [2M + Cu + H]^+^, 723 [Au(PyPhos)_2_]^+^, IR (cm^–1^): ν = 2962.34 (ar), 2012.93 (C≡C), 1584.88 (C=N), 1098.50 (P–C), 835.68 (P–F).

#### [(PyPh_2_P)_4_Cu_4_Au_4_(C≡CNaph)_4_][OTf]_4_ (**Cu1b**)

In a sealed flask under an N_2_ atmosphere **complex 1** (10 mg, 0.0178 mmol, 1 equiv) was added in 3 mL of CH_2_Cl_2_ under stirring for 5 min. Then, [Cu(MeCN)_4_]OTf (6.16 mg, 0.0178 mmol, 1 equiv) dissolved in 2 mL of CH_2_Cl_2_ was added. After 3 h, the solution was reduced to dryness under vacuum. The solid was dissolved in 1 mL of dichloromethane and precipitated with 2 mL of hexane. **Cu1b** was obtained as an orange solid (10.2 mg, 85%). ^1^H NMR: (CDCl_3_, 400 MHz) δ = δ 8.79 (s, 1H), 8.03 (s, 1H), 7.80 (dd, *J* = 8.6, 5.4 Hz, 2H), 7.69 (dd, *J* = 13.5, 7.4 Hz, 4H), 7.57–7.32 (m, 13H) ppm; ^19^F NMR: (CDCl_3_ 162 MHz) δ = −78.12 ppm (s, 3F), ^31^P NMR: (CDCl_3_ 162 MHz) δ = 42.4 (s) ppm. ESI (*m*/*z*): 1896.24 [3M + Cu + H]^+^, 1383.04 [2M + 2Cu + Cl + H]^+^, 1285.14 [2M + Cu + H]^+^, 1071.15 [2M + H]^+^, 723.13 [Au(PyPhos)_2_]^+^, IR (cm^–1^): ν = 2961.91 (ar), 2042.32 (C≡C), 1585.12 (C=N), 1458.15 (C–H), 1258.00 (B–F), (P–C),1093.55 (P–C).

#### [(PyPh_2_P)_4_Cu_4_Au_4_(C≡CNaph)_4_][BF_4_]_4_ (**Cu1c**)

In a sealed flask under a N_2_ atmosphere, complex 1 (10 mg, 0.0178 mmol, 1 equiv) was added to 3 mL of CH_2_Cl_2_ under stirring for 5 min. Then, [Cu(MeCN)_4_]BF_4_ (5.12 mg, 0.0178 mmol, 1 equiv) dissolved in 2 mL of CH_2_Cl_2_ was added. After 3 h, the solution was reduced to dryness under vacuum. The solid was dissolved in 1 mL of dichloromethane and precipitated with 2 mL of hexane. **Cu1c** was obtained as a red-orange solid (9.1 mg, 78%). ^1^H NMR: (CDCl_3_, 400 MHz) δ = 8.80 (s, 1H), 8.0 (m, 2H), 7.86–7.76 (m, 3H), 7.70 (dd, *J* = 13.3, 7.5 Hz, 5H), 7.52 (d, *J* = 7.5 Hz, 2H), 7.47 (d, *J* = 6.1 Hz, 3H), 7.44–7.34 (m, 2H) ppm; ^19^F NMR: (CDCl_3_ 162 MHz) δ = −150.29 ppm (s, 4F), ^31^P NMR: (CDCl_3_ 162 MHz) δ = 32.27 (s) ppm. ESI (*m*/*z*): 1346.93 [2M + 2Cu + H]^+^, 1071.10 [2M + H]^+^, 723.13 [Au(PyPhos)_2_]^+^, IR (cm^–1^): ν = 2962.08 (ar), 2024.47 (C≡C), 1586.24 (C=N), 1098.19 (P–C), 1054.06 (B–F).

#### [(PyPh_2_P)AuC≡CPhen] (**2**)

KOH (17 mg, 0.303 mmol, 1.5 equiv) was added to a methanol (5 mL) solution under a N_2_ atmosphere. 9-Ethynylphenanthrene (40.8 mg, 0.202 mmol, 1 equiv) was added. The mixture was stirred for 2 h at 25 °C. Then, a solution of [(PyPh_2_P)AuCl] (100 mg, 0.202 mmol, 1 equiv) in 3 mL of dichloromethane was slowly added. After 24 h, the solvents were removed under vacuum. The solid was dissolved in 1 mL of dichloromethane and precipitated with 3 mL of hexane. **2** was obtained as a white solid (52 mg, 46%). ^1^H NMR: (DMSO-*d*_6_ 400 MHz) δ = 8.90–8.72 (m, 3H), 8.67–8.54 (m, 1H), 8.07–7.89 (m, 3H), 7.84–7.54 (m, 16H) ppm; ^31^P NMR: (DMSO-*d*_6_ 162 MHz) δ = 40.41 ppm. ESI (*m*/*z*): 1345.23 [2M + Na]^+^, 1121.17 [2M-Phen], 723.13 [Au(PyPhos)_2_]^+^, 662.12 [M + H]^+^, IR (cm^–1^): ν = 3054.27 (ar), 2112.14 (C≡C), 1568.12 (C=N), 1478.04 (C–H), 1100.13 (P–C).

#### [(PyPh_2_P)_4_Cu_4_Au_4_(C≡CPhen)_4_][PF_6_]_4_ (**Cu2a**)

In a sealed flask under a N_2_ atmosphere, **complex 2** (10 mg, 0.0178 mmol, 1 equiv) was added in 3 mL of CH_2_Cl_2_ under stirring for 5 min. Then, [Cu(MeCN)_4_]PF_6_ (6.09 mg, 0.0178 mmol, 1 equiv) dissolved in 2 mL of CH_2_Cl_2_ was added. After 3 h, the solution was reduced to dryness under vacuum. The solid was dissolved in 1 mL of dichloromethane and precipitated with 2 mL of hexane. **Cu2a** was obtained as a red-orange solid (9.6 mg, 82%). ^1^H NMR: (CDCl_3_, 400 MHz) δ = 9.35 (s, 1H), 8.56 (dd, *J* = 16.2, 7.8 Hz, 3H), 8.45 (d, *J* = 7.9 Hz, 2H), 8.07 (s, 2H), 7.78 (s, 1H), 7.60–7.65 (m, 3H). 7.62 (d, *J* = 7.6 Hz, 1H), 7.49 (dd, *J* = 15.2, 7.7 Hz, 1H), 7.39–7.29 (m, 3H), 7.05 (dd, *J* = 7.8 Hz, 1H), 6.74 (s, 1H), 6.44–6.25 (m, 4H) ppm; ^19^F NMR: (CDCl_3_ 162 MHz) δ = −73.65 ppm (d, *J*_P–F_ 712.0 Hz, 6F), ^31^P NMR: (CDCl_3_ 162 MHz) δ = 39.73 (s), −146.98 (hept) ppm. ESI (*m*/*z*): 2046.28 [3M + Cu + H]^+^, 1483.07 [2M + 2Cu + Cl + H]^+^, 1385.17 [2M + Cu + H]^+^, 723.13 [Au(PyPhos)_2_]^+^, IR (cm^–1^): ν = 3057.16 (ar), 2005.33 (C≡C), 1584.29 (C=N), 1132.45 (P–C), 834.20 (P–F).

#### [(PyPh_2_P)_4_Cu_4_Au_4_(C≡CPhen)_4_][OTf]_4_ (**Cu2b**)

In a sealed flask under a N_2_ atmosphere, **complex 2** (10 mg, 0.0178 mmol, 1 equiv) was added in 3 mL of CH_2_Cl_2_ under stirring for 5 min. Then, [Cu(MeCN)_4_]OTf (6.09 mg, 0.0178 mmol, 1 equiv) dissolved in 2 mL of CH_2_Cl_2_ was added. After 3 h, the solution was reduced to dryness under vacuum. The solid was dissolved in 1 mL of dichloromethane and precipitated with 2 mL of hexane. **Cu2b** was obtained as a red-orange solid (10.1 mg, 88%). ^1^H NMR: (CDCl_3_, 400 MHz) δ = 9.37 (s, 1H), 8.59 (d, *J* = 9.1 Hz, 1H), 8.55 (d, *J* = 8.3 Hz, 1H), 8.53 (d, *J* = 7.1 Hz, 1H), 8.17 (s, 1H), 7.93–7.84 (m, 1H), 7.72–7.66 (m, 1H), 7.64 (t, *J* = 7.8 Hz, 1H), 7.55–7.44 (m, 1H), 7.43–7.31 (m, 8H), 7.06 (d, *J* = 11.5 Hz, 1H), 6.77 (s, 1H), 7.50–7.29 (m, 4H) ppm; ^19^F NMR: (CDCl_3_ 162 MHz) δ = −77.96 (s, 3F) ppm,^31^P NMR: (CDCl_3_ 162 MHz) δ = 39.91 (s) ppm. ESI (*m*/*z*): 2047.22 [3M + Cu + H]^+^, 1485.06 [2M + 2Cu + Cl + H]^+^, 1385.17 [2M + Cu + H]^+^, 1121.17 [2M + H]^+^ 723.13 [Au(PyPhos)_2_]^+^. IR (cm^–1^): ν = 2961.97 (ar), 2005.52 (C≡C), 1524.67 (C=N), 1257.80 (CF_3_), 1098.67 (P–C).

#### [(PyPh_2_P)_4_Cu_4_Au_4_(C≡CPhen)_4_][BF_4_]_4_ (**Cu2c**)

In a sealed flask under a N_2_ atmosphere, **complex 2** (10 mg, 0.0178 mmol, 1 equiv) was added in 3 mL of CH_2_Cl_2_ under stirring for 5 min. Then, [Cu(MeCN)_4_]BF_4_ (6.09 mg, 0.0178 mmol, 1 equiv) dissolved in 2 mL of CH_2_Cl_2_ was added. After 3 h, the solution was reduced to dryness under vacuum. The solid was dissolved in 1 mL of dichloromethane and precipitated with 2 mL of hexane. **Cu2c** was obtained as a red-orange solid (8.3 mg, 77%). ^1^H NMR: (CDCl_3_, 400 MHz) δ = 9.36 (s, 1H), 8.59 (s, 1H), 8.54 (d, *J* = 8.3 Hz, 1H), 8.46 (d, *J* = 7.7 Hz, 2H), 8.14 (s, 2H), 7.89–7.80 (m, 1H), 7.70–7.65 (m, 3H), 7.63 (t, *J* = 7.7 Hz, 2H), 7.52–7.44 (m, 2H), 7.39–7.31 (m, 2H), 7.06 (d, *J* = 12.0 Hz, 1H), 7.66 (s, 1H), 7.46–7.29 (m, 4H) ppm; ^19^F NMR: (CDCl_3_ 162 MHz) δ = −153.43 ppm (s, 4F), ^31^P NMR: (CDCl_3_ 162 MHz) δ = 39.77 (s) ppm. ESI (*m*/*z*): 1483.07 [2M + 2Cu + Cl + H]^+^, 1385.17 [2M + Cu + H]^+^, 1121.17 [M + Au + H]^+^, 723.13 [Au(PyPhos)_2_]^+^, IR (cm^–1^): ν = 2905.04 (ar), 2077.07 (C≡C), 1584.96 (C=N), 1258.61 (B–F), 1012.17 (P–C).

## Supporting Information Available

^1^H and ^31^P NMR spectra for all complexes; ESI-TOF spectra for all complexes; absorption and emission spectra for all compounds; X-ray crystal packing of **Cu1a**; absorption and emission spectra of all compounds in solution (air-equilibrated and N_2_-saturated samples), solid and organic polymer matrices (PMMA and PS); emission lifetime data of all compounds; emission spectra for the calculation of singlet oxygen production; and crystal data and structure refinement for the **Cu1a**, **Cu2a**, and **Cu2c** (PDF)
