# Supplementary material for: Heterometallic Au(I)–Cu(I) Clusters: Luminescence Studies and 1O2 Production
Source: Inorg Chem. 2023 May 16;62(21):8101–11. doi: 10.1021/acs.inorgchem.3c00046 (PMC10230500; doi:10.1021/acs.inorgchem.3c00046)
Supplement: Supplementary file 1 — ic3c00046_si_001.pdf [file ic3c00046_si_001.pdf]

Supporting Information

Heterometallic Au(I)-Cu(I) clusters. Luminescence studies and $^1O_2$ production

Guillermo Romo-Islas,[a,b] Jas S. Ward,[c] Kari Rissanen,[c] and Laura Rodríguez[a,b,*]

[a]*Departament de Química Inorgànica i Orgànica. Secció de Química Inorgànica. Universitat de Barcelona, Martí i Franquès 1-11, 08028 Barcelona, Spain. E-mail: laura.rodriguez@qi.ub.es*

[b]*Institut de Nanociència i Nanotecnologia (IN2UB), Universitat de Barcelona, 08028 Barcelona, Spain.*

[c] *Department of Chemistry, Nanoscience Center, University of Jyvaskyla, P.O. Box 35, 40014 Jyvaskyla, Finland*

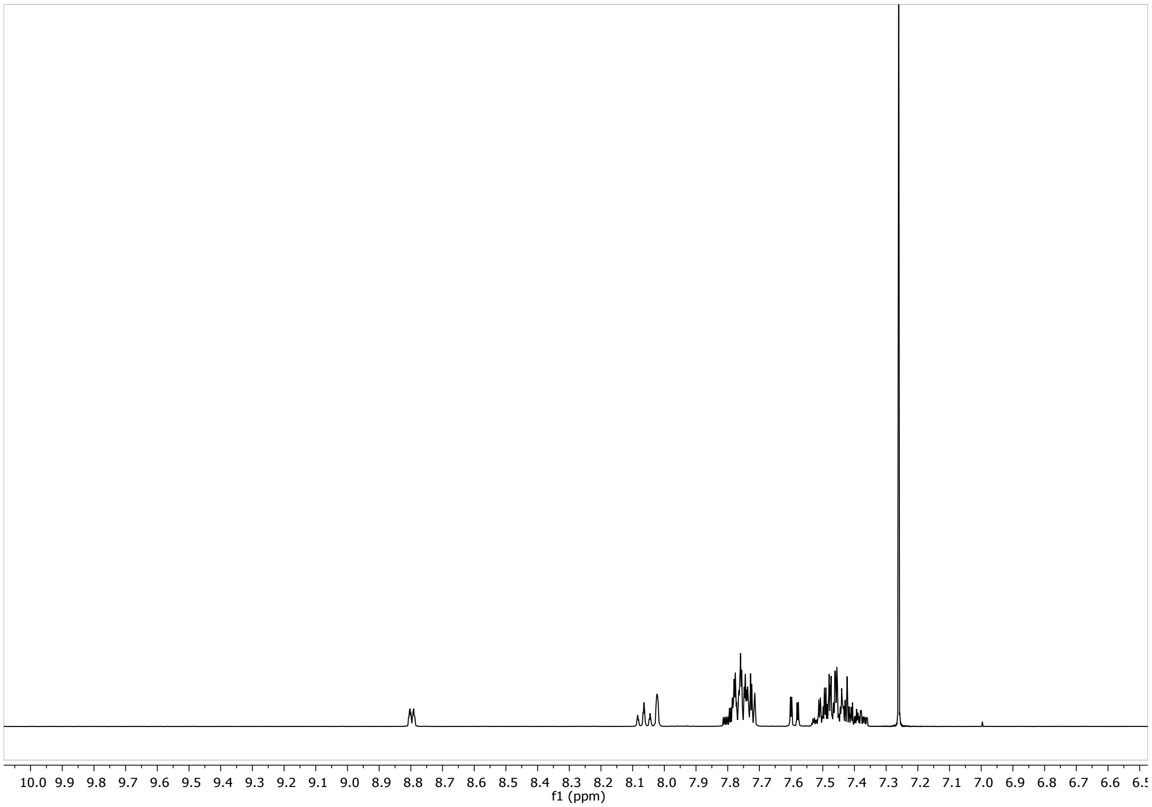

Fig. S1.- ^{1}H NMR spectrum of complex **1** in $CDCl_3$.

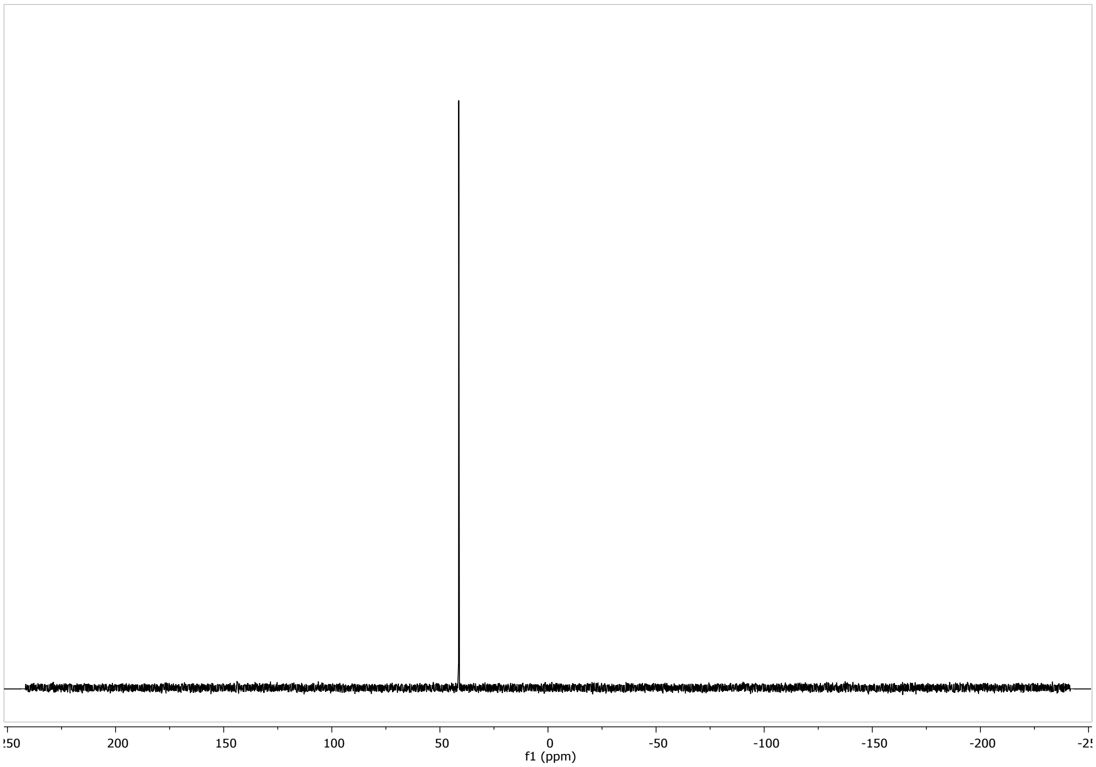

Fig. S2.- ^{31}P NMR spectrum of complex **1** in $CDCl_3$.

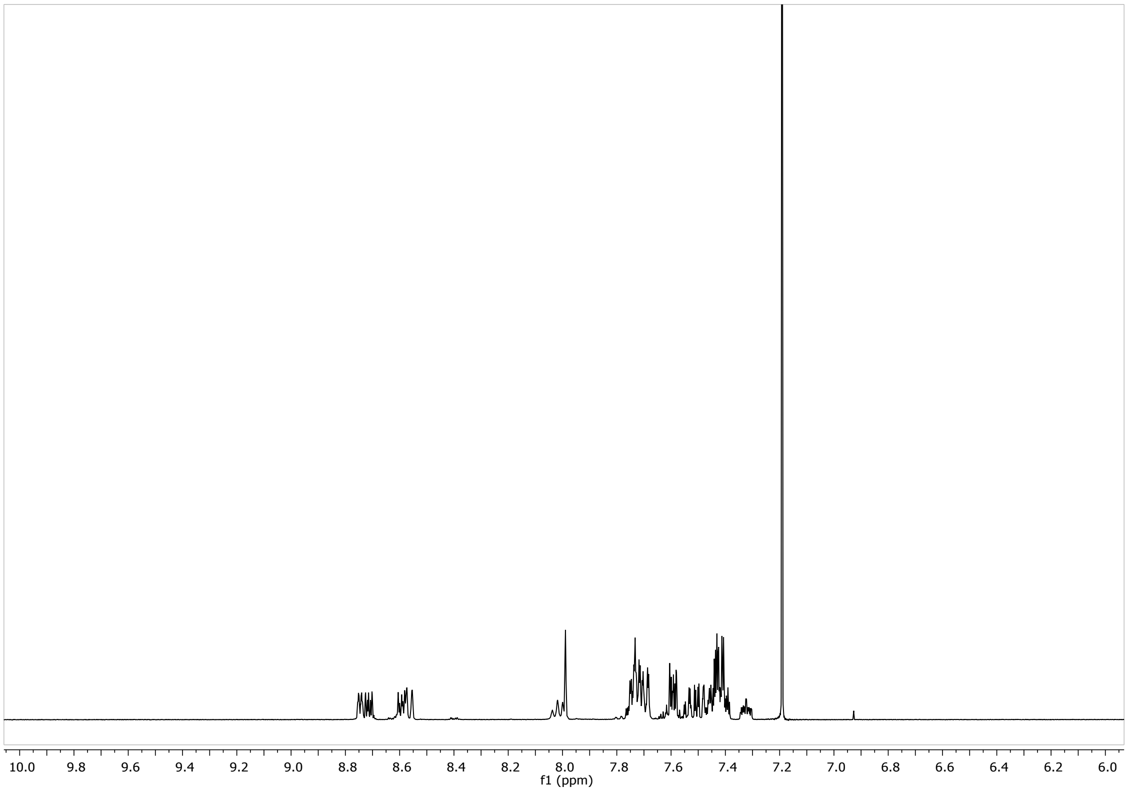

Fig. S3.- ^{1}H NMR spectrum of complex **2** in $CDCl_3$.

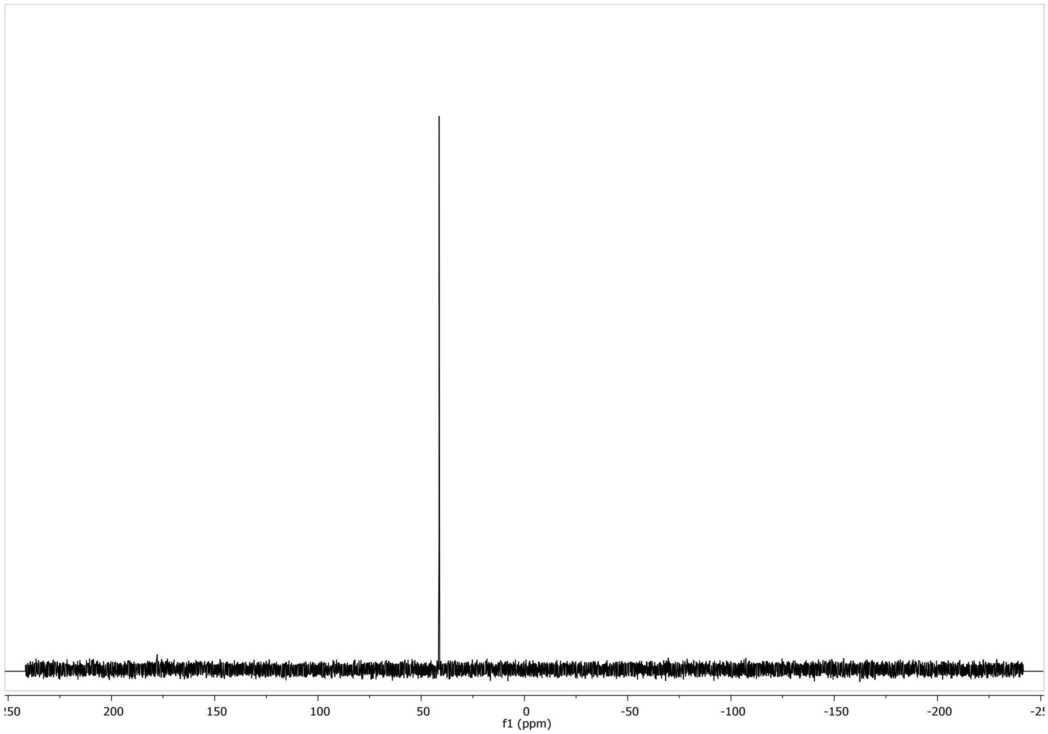

Fig. S4.- ^{31}P NMR spectrum of complex **2** in $CDCl_3$.

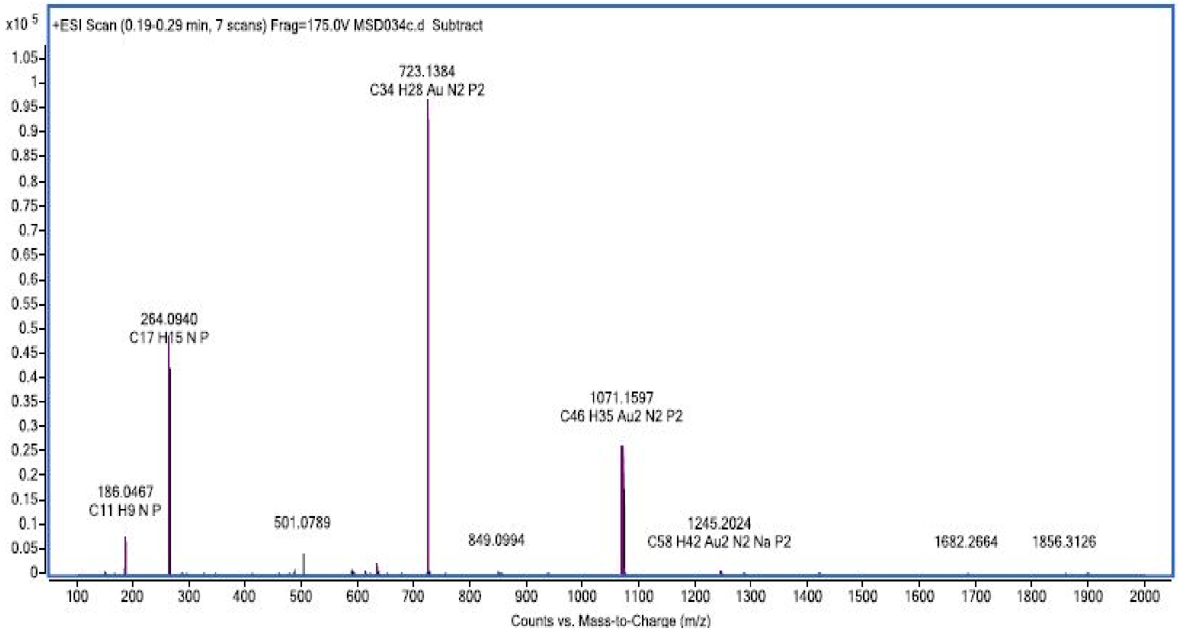

Fig. S5.- ESI-TOF(+) of complex **1**.

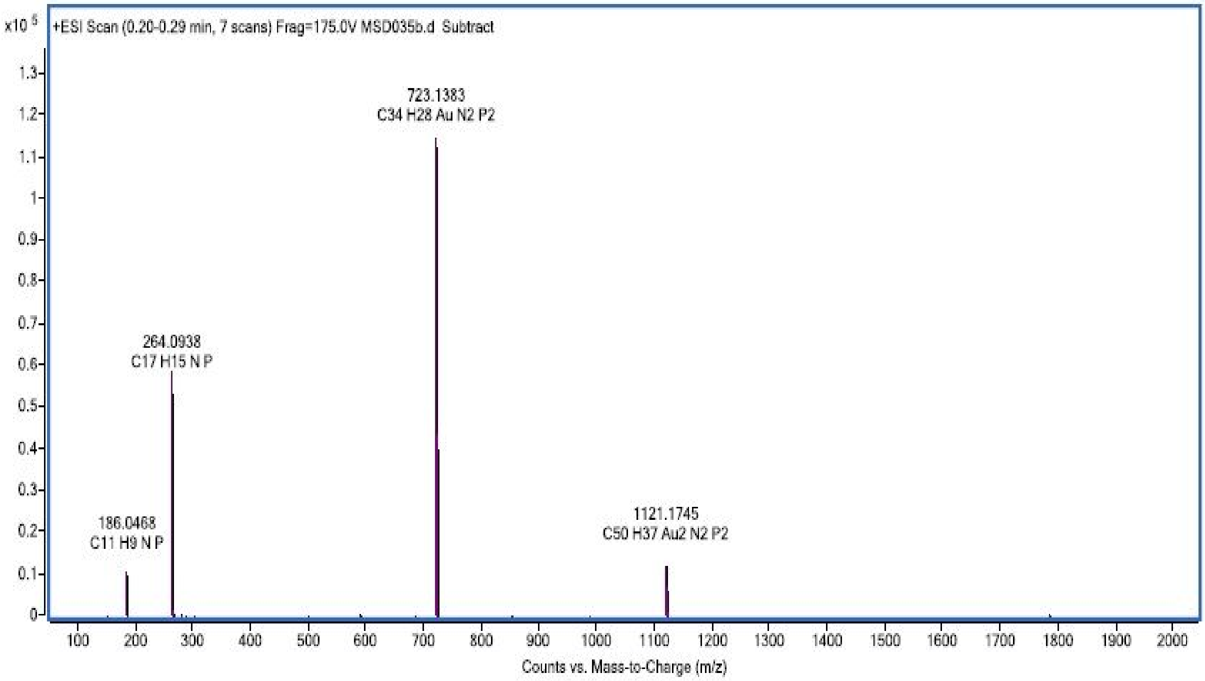

Fig. S6.- ESI-TOF(+) of complex **2**.

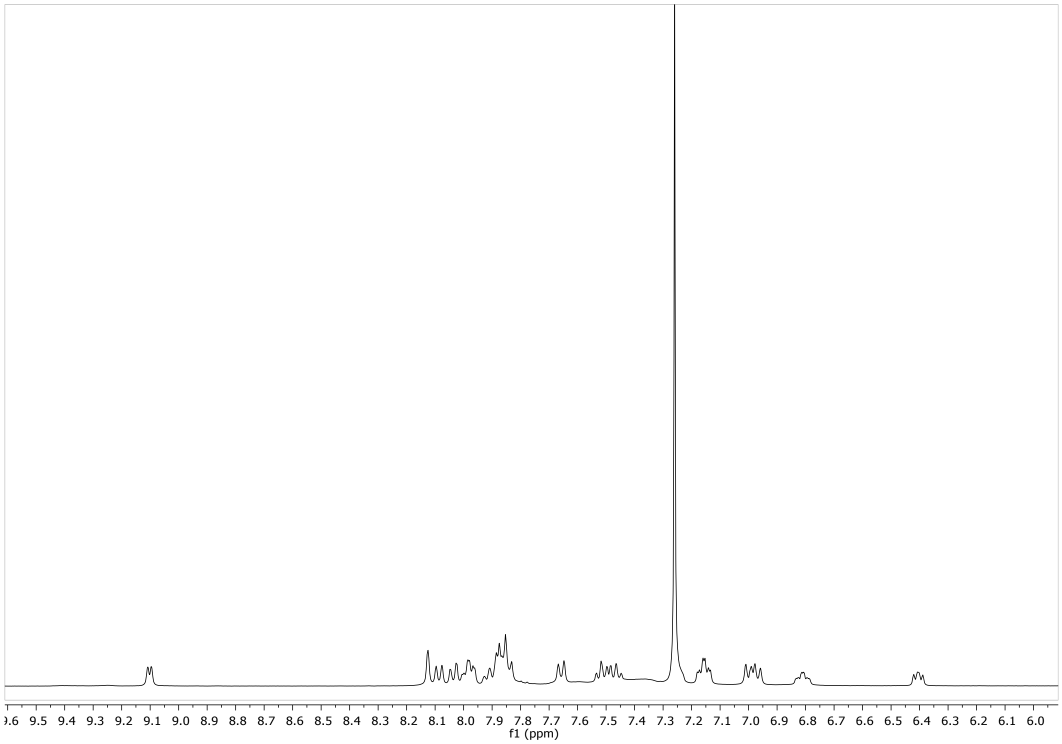

Fig. S7.- ^{1}H NMR spectrum of complex **Cu1a** in $CDCl_3$.

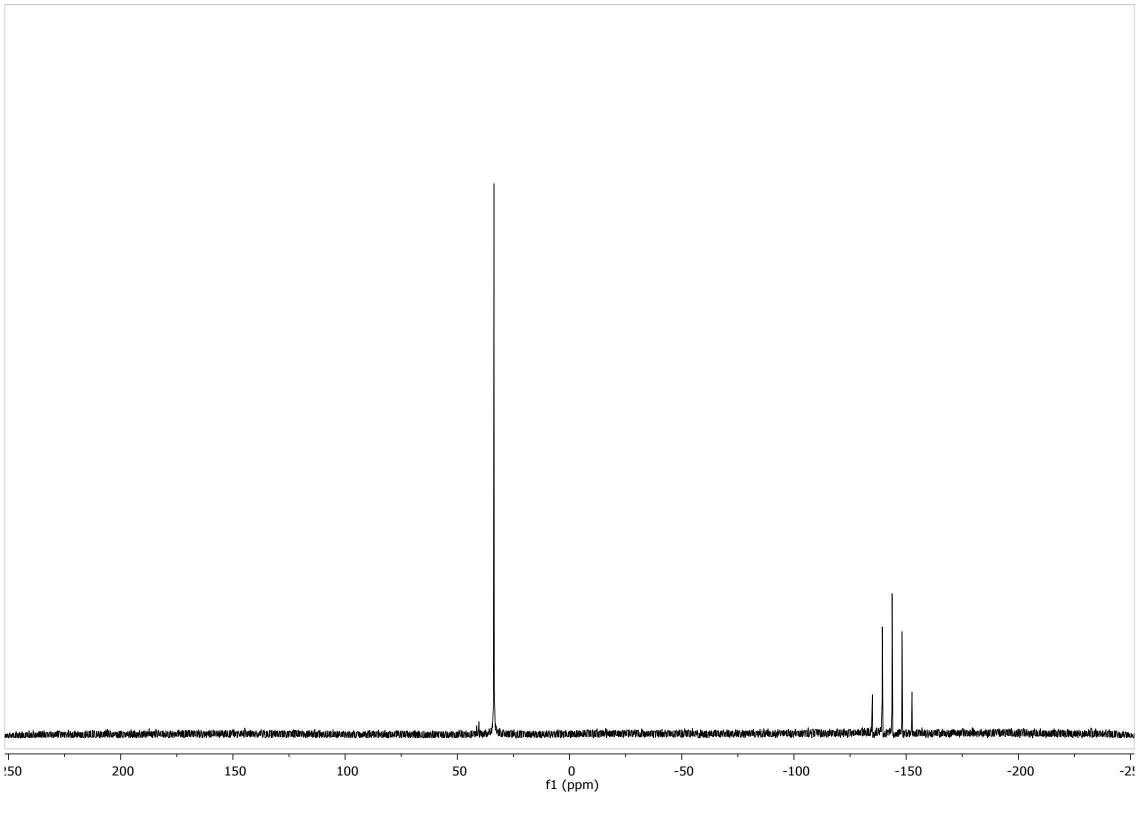

Fig. S8.- ^{31}P NMR spectrum of complex **Cu1a** in $CDCl_3$.

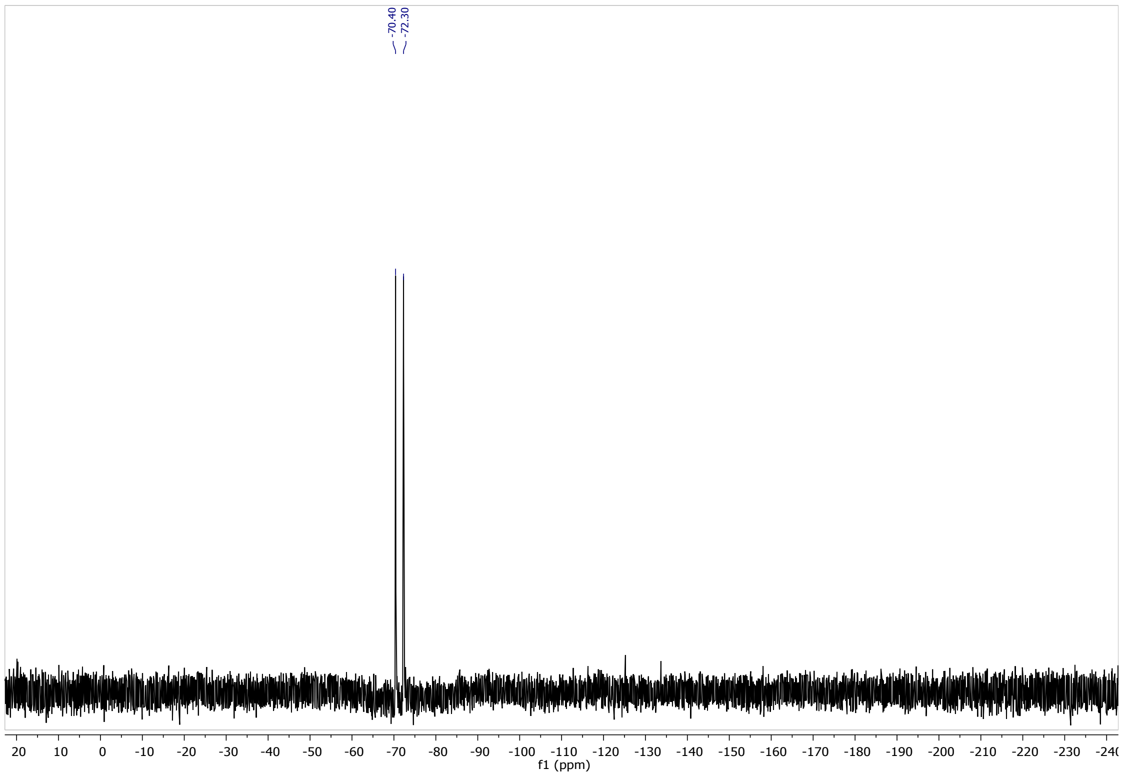

Fig. S9.- ^{19}F NMR spectrum of complex **Cu1a** in $CDCl_3$.

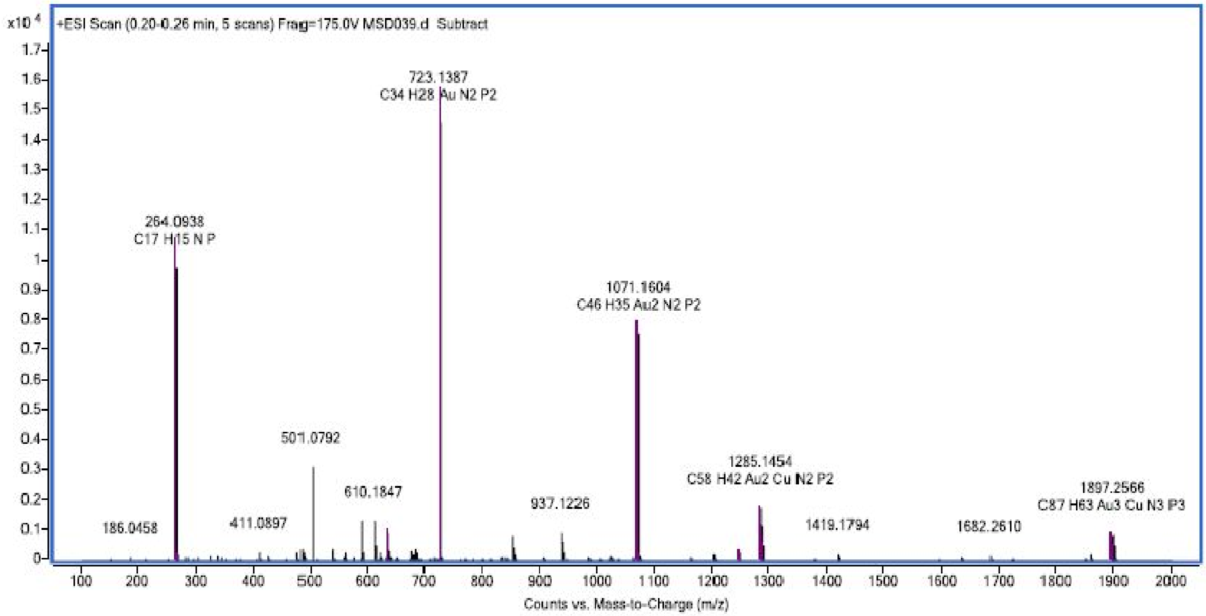

Fig. S10.- ESI-TOF(+) of complex **Cu1a**.

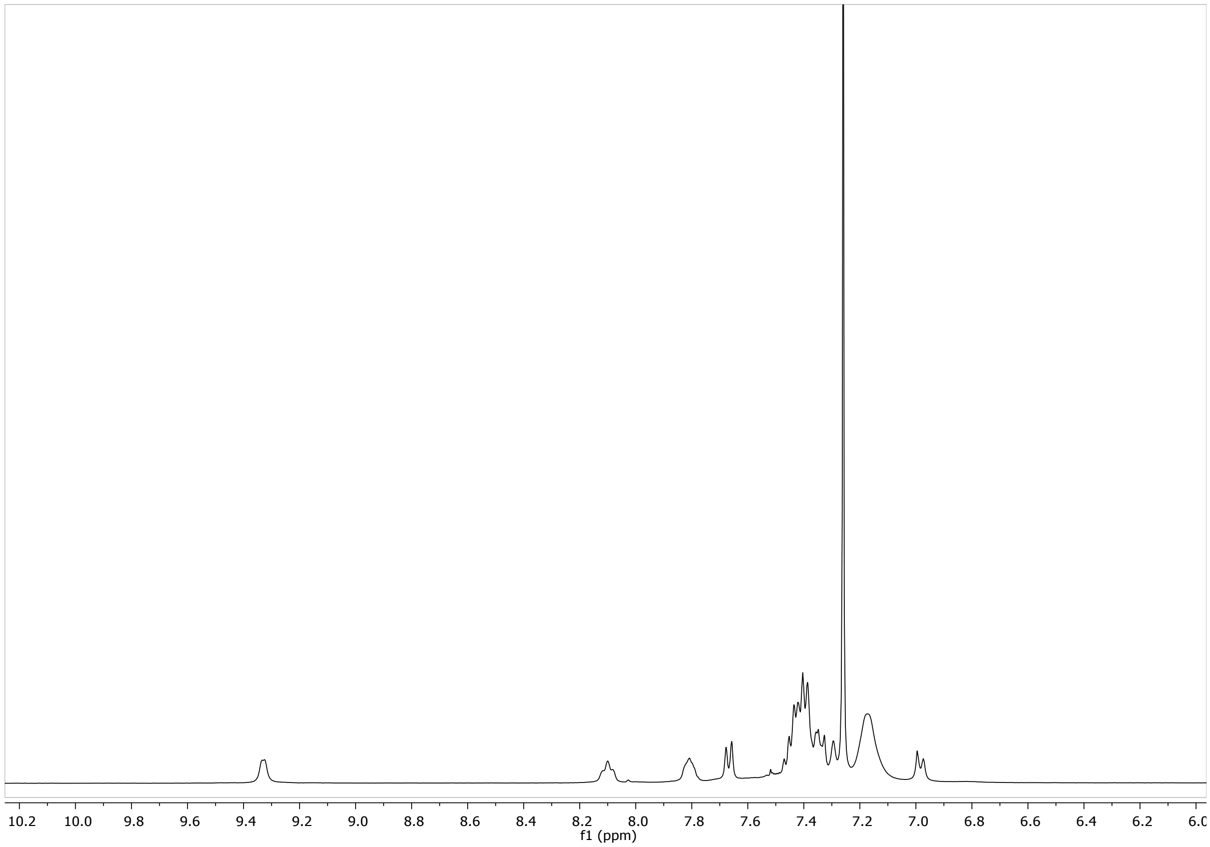

Fig. S11.- ^{1}H NMR spectrum of complex **Cu1b** in $CDCl_3$.

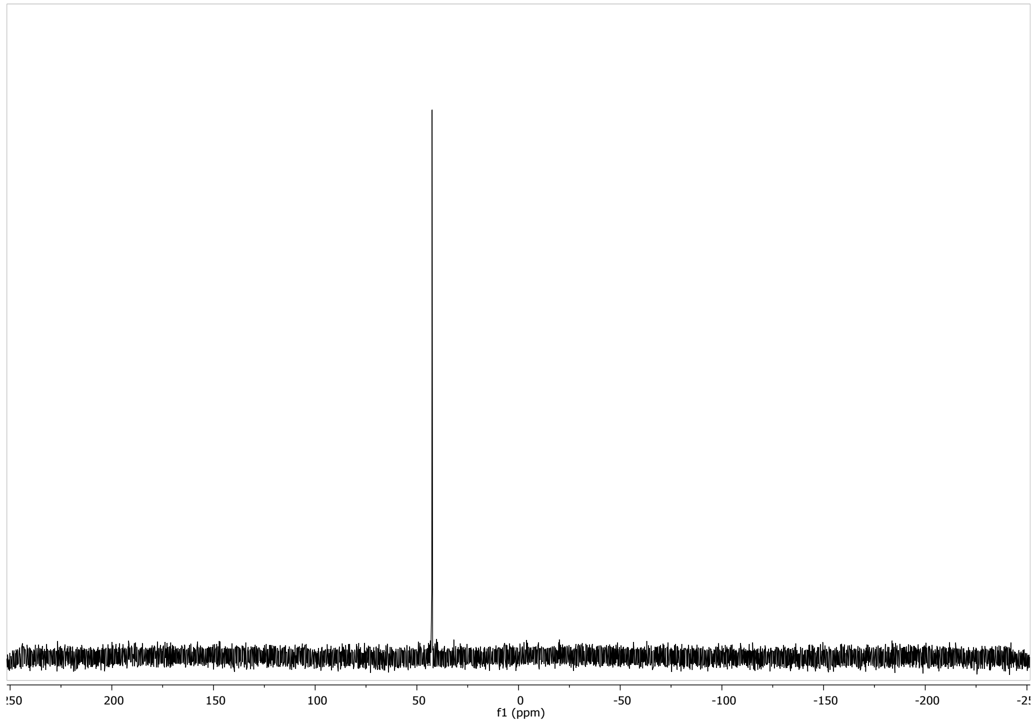

Fig. S12.- ^{31}P NMR spectrum of complex **Cu1b** in $CDCl_3$.

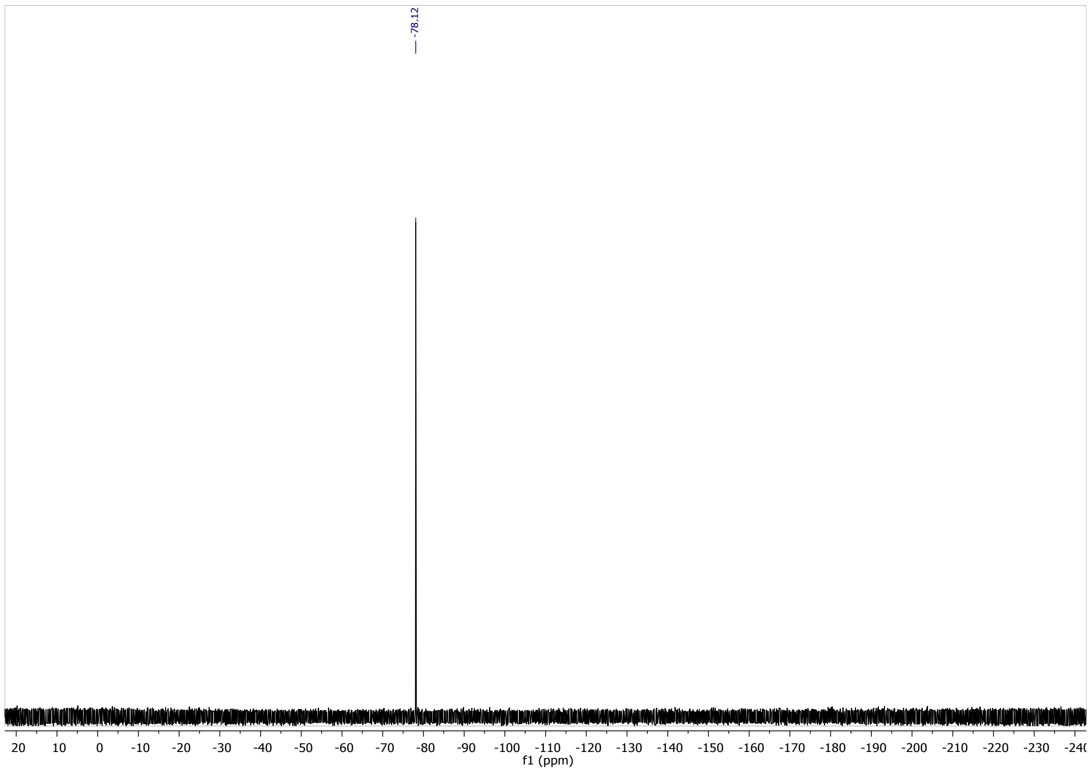

Fig. S13.- ^{19}F NMR spectrum of complex **Cu1b** in $CDCl_3$.

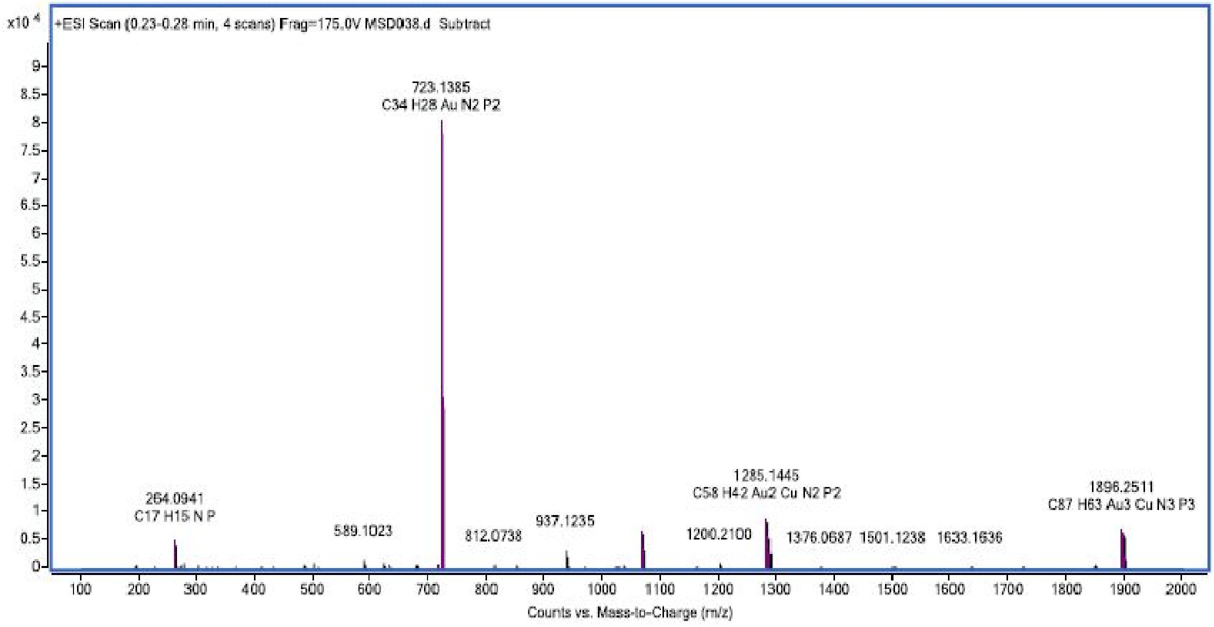

Fig. S14.- ESI-TOF(+) of complex **Cu1b**.

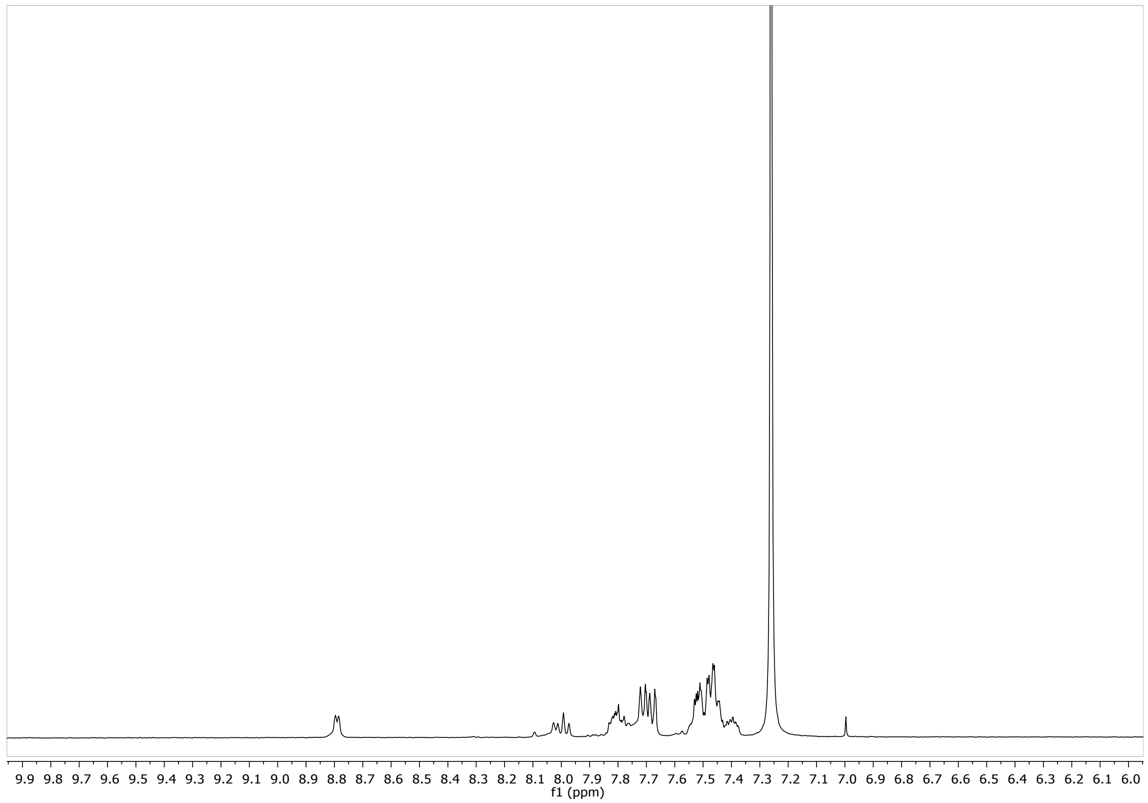

Fig. S15.- ^{1}H NMR spectrum of complex **Cu1c** in $CDCl_3$.

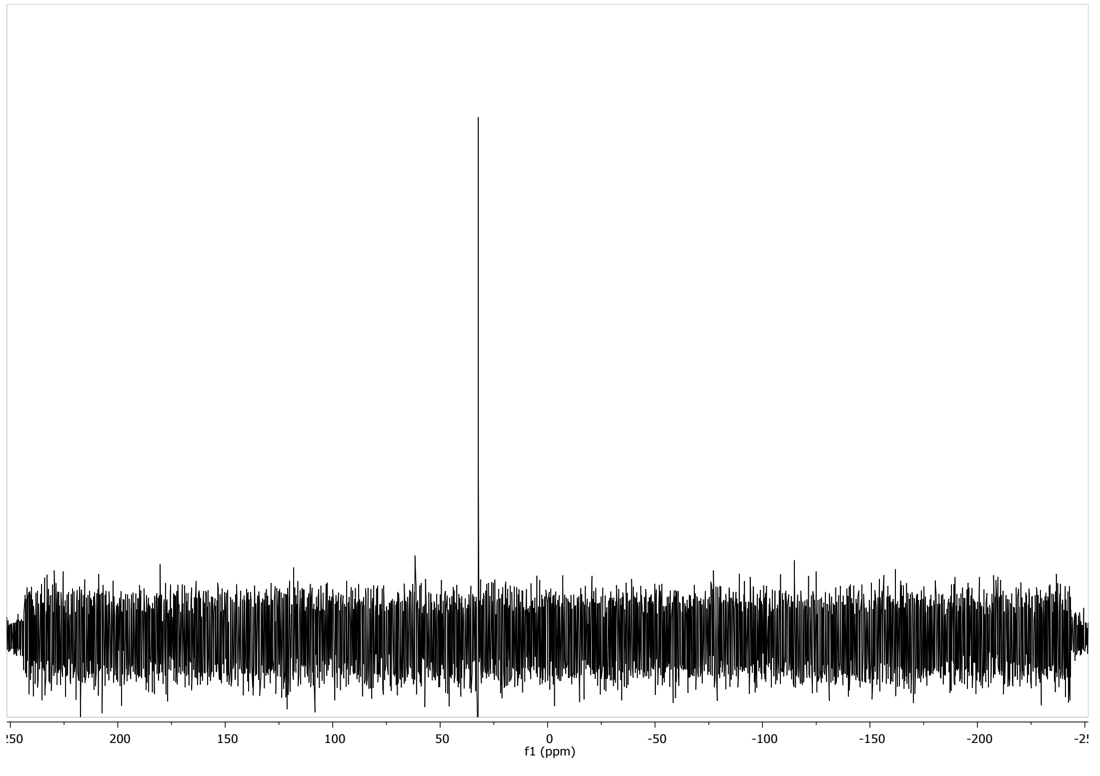

Fig. S16.- ^{31}P NMR spectrum of complex **Cu1c** in $CDCl_3$.

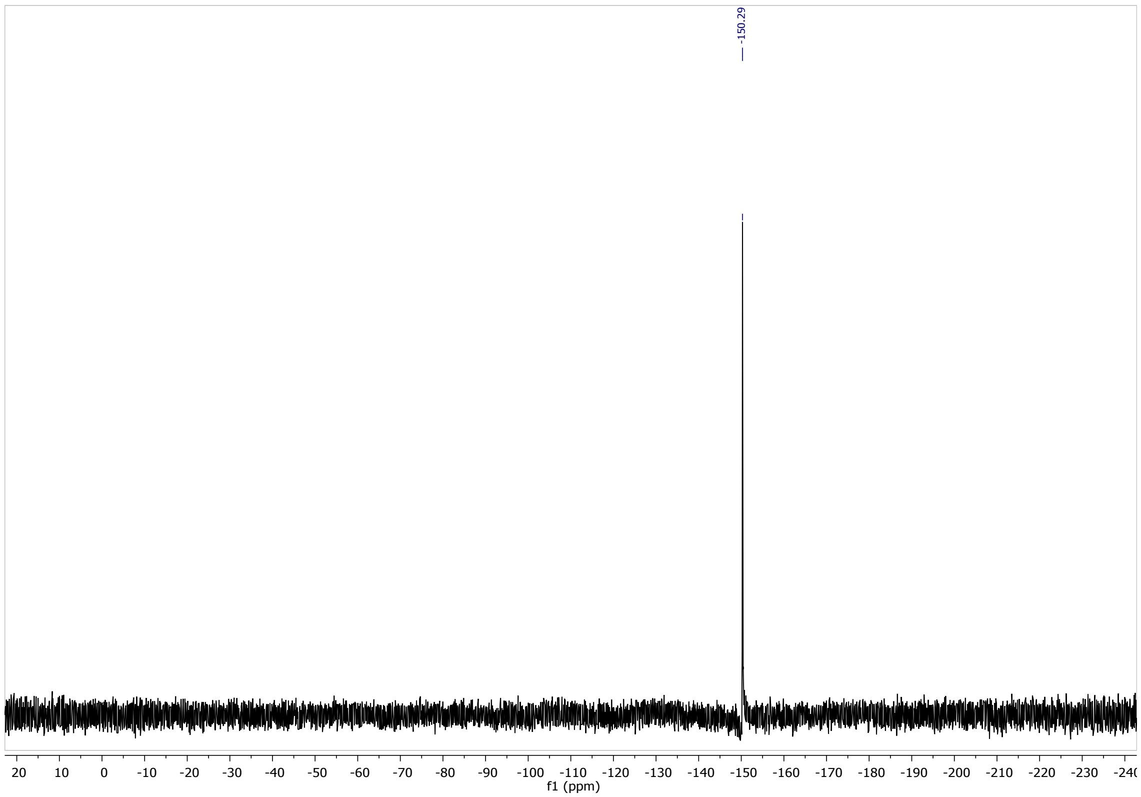

Fig. S17.- ^{19}F NMR spectrum of complex **Cu1c** in $CDCl_3$.

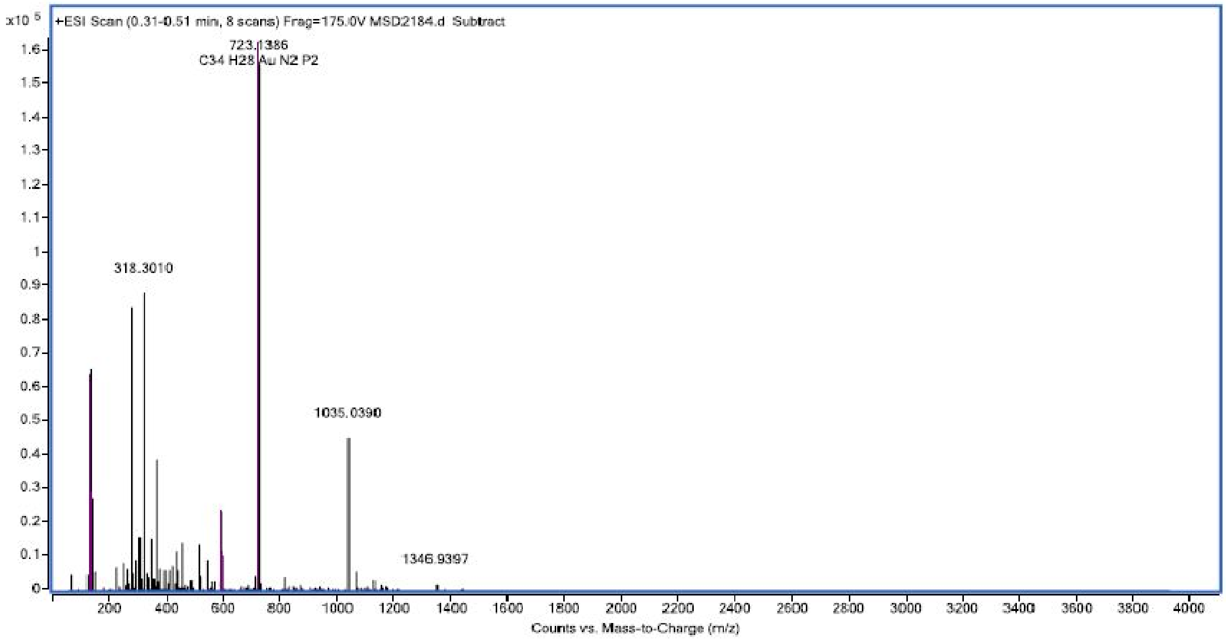

Fig. S18.- ESI-TOF(+) of complex **Cu1c**.

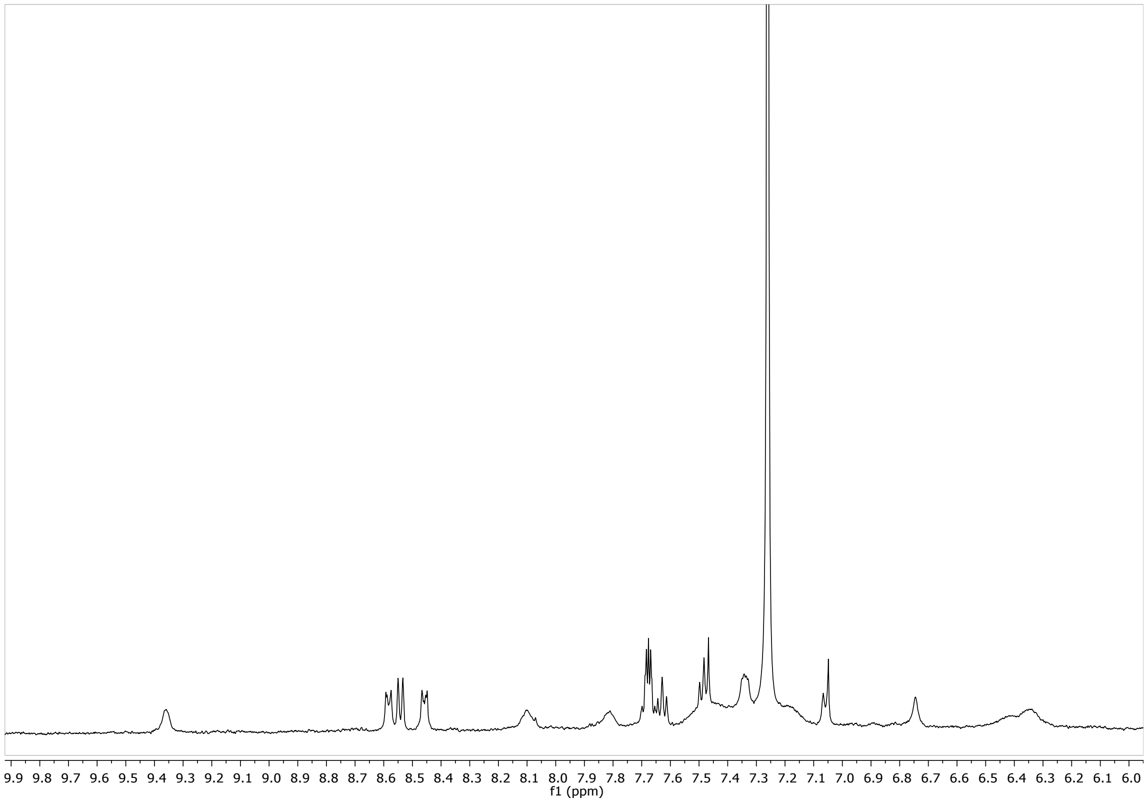

Fig. S19.- ^{1}H NMR spectra of complex **Cu2a** in $CDCl_3$.

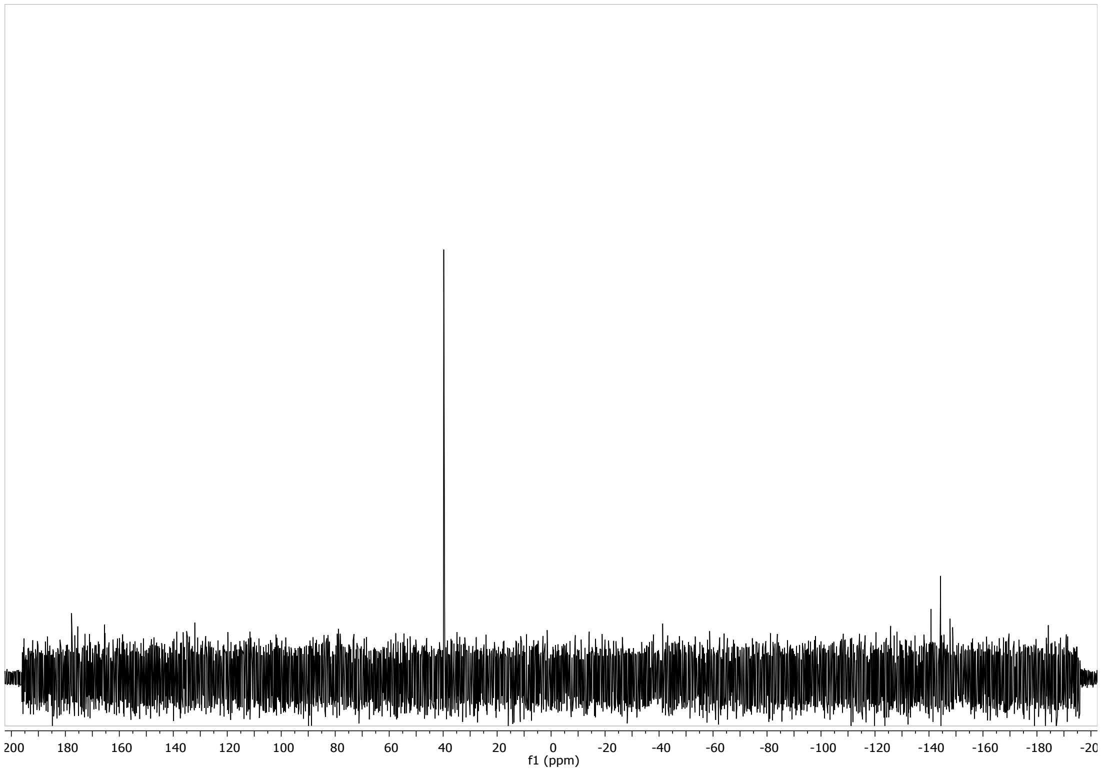

Fig. S20.- ^{31}P NMR spectra of complex **Cu2a** in $CDCl_3$.

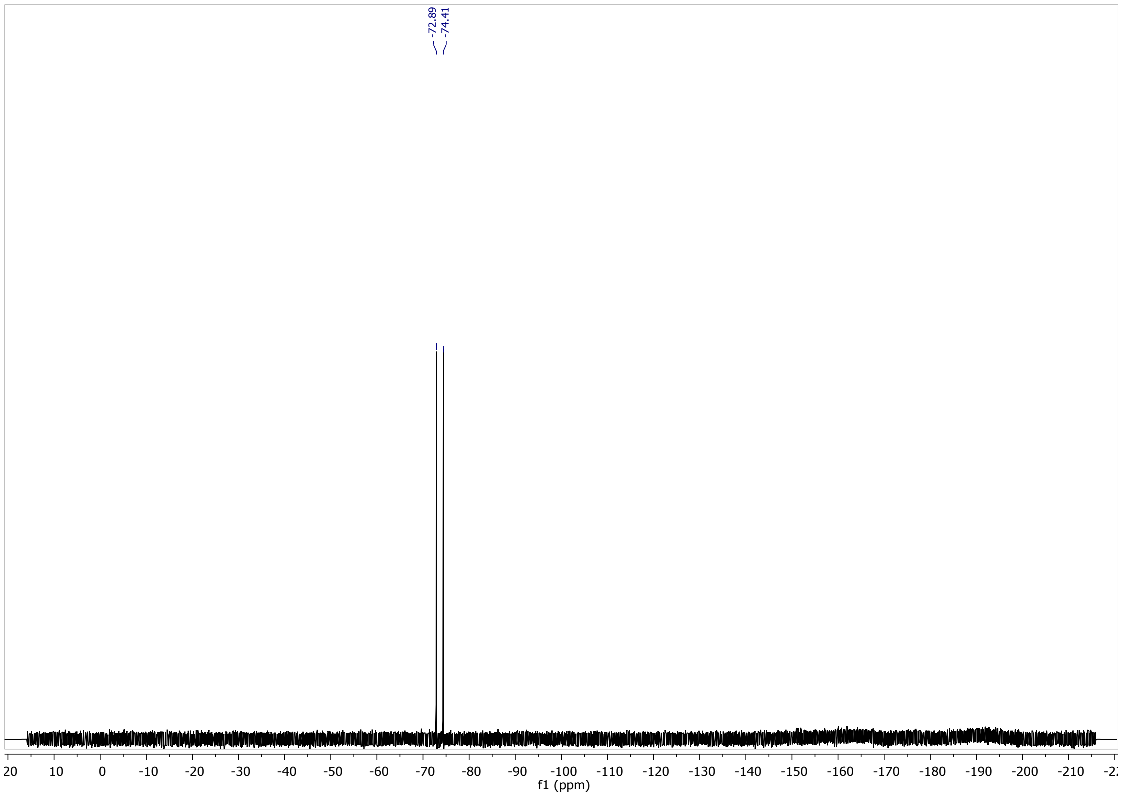

Fig. S21.- ^{19}F NMR spectra of complex **Cu2a** in $CDCl_3$.

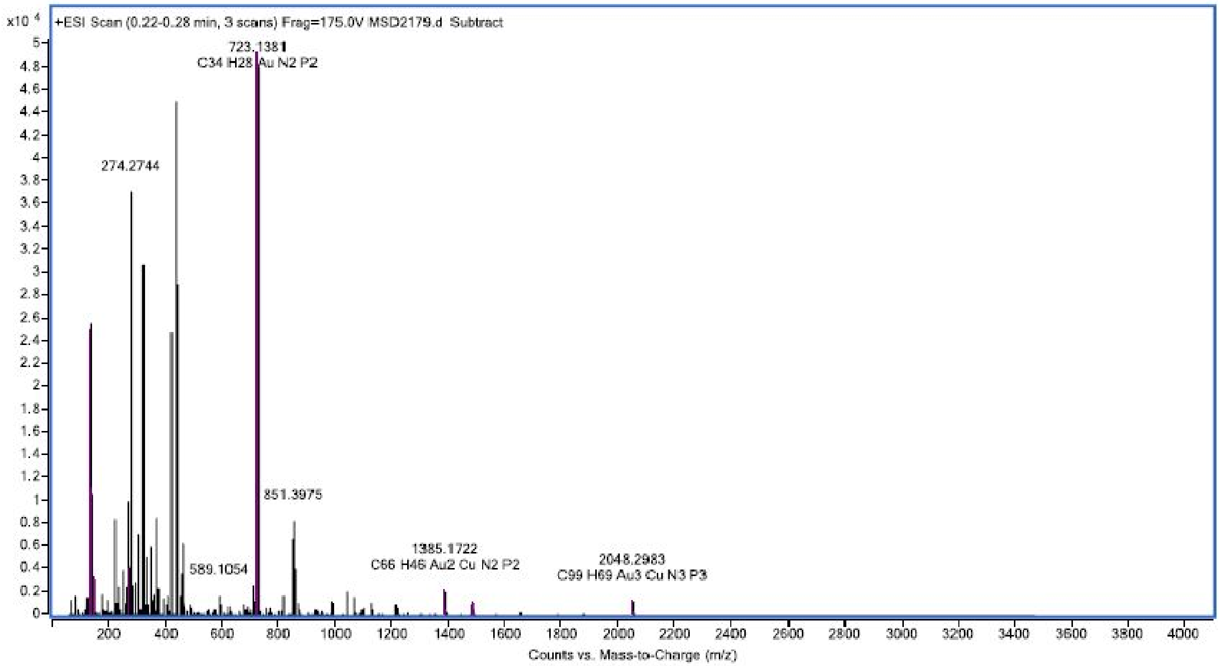

Fig. S22.- ESI-TOF(+) of complex **Cu2a**.

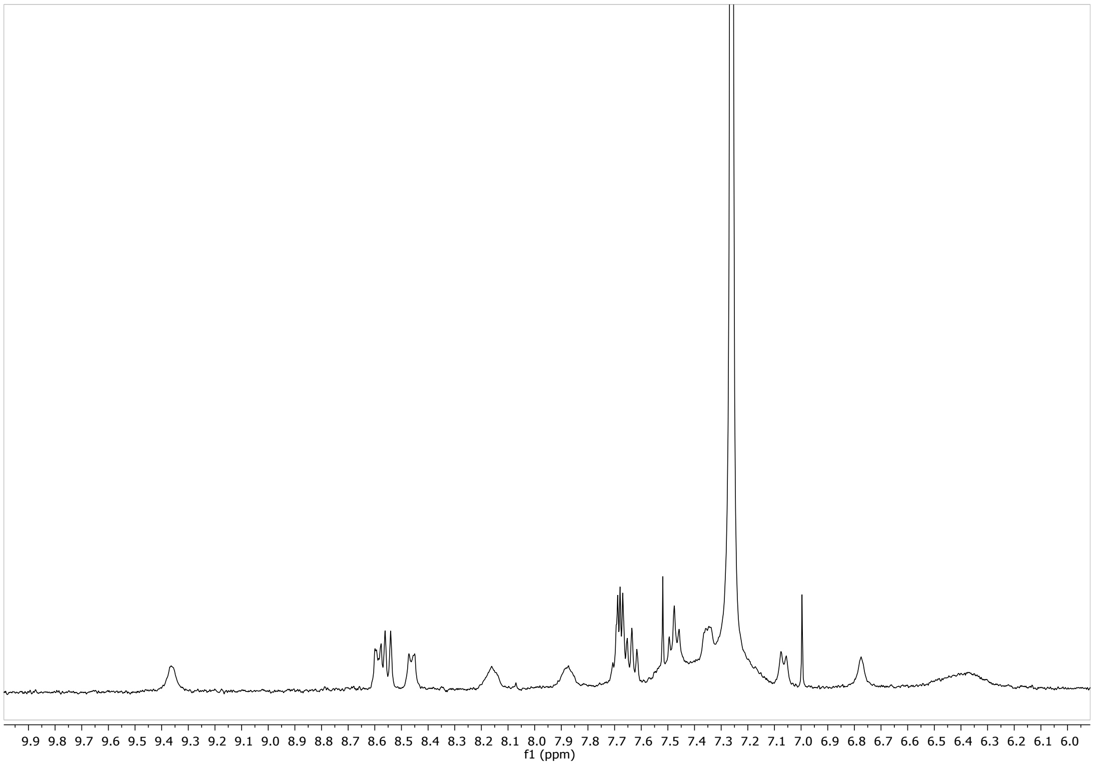

Fig. S23.- ^{1}H NMR spectrum of complex **Cu2b** in $CDCl_3$.

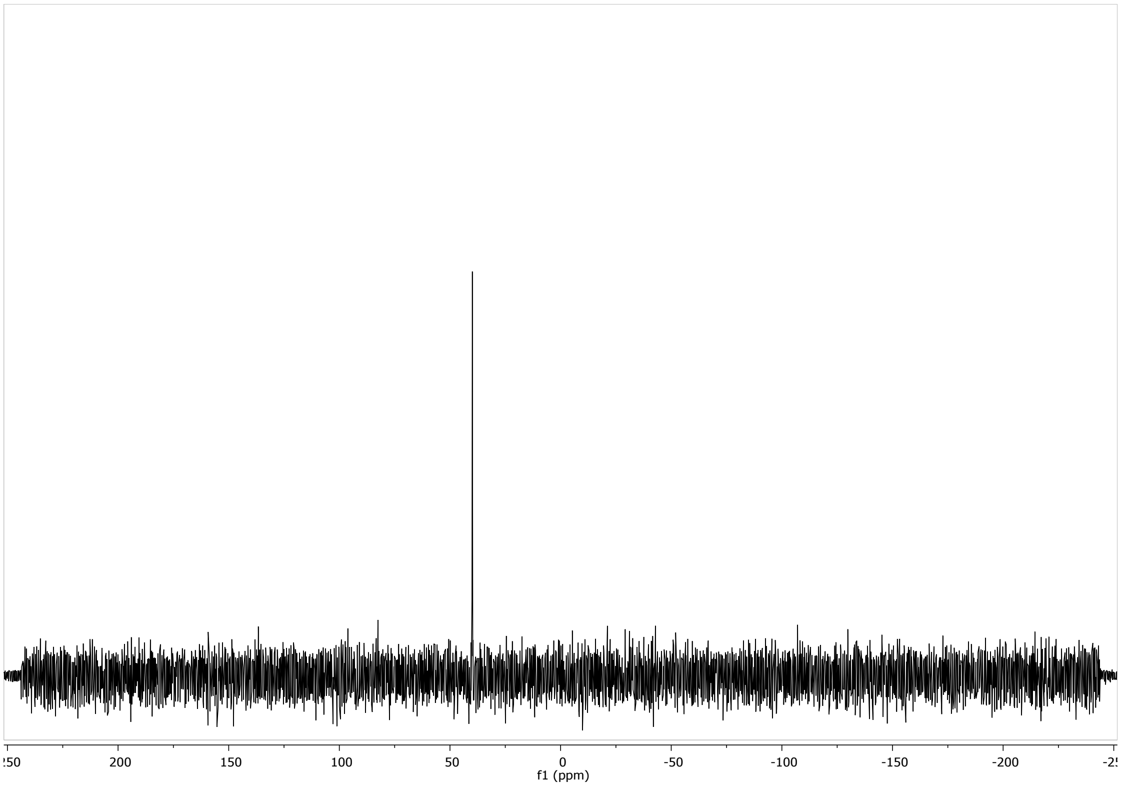

Fig. S24.- ^{31}P NMR spectrum of complex **Cu2b** in $CDCl_3$.

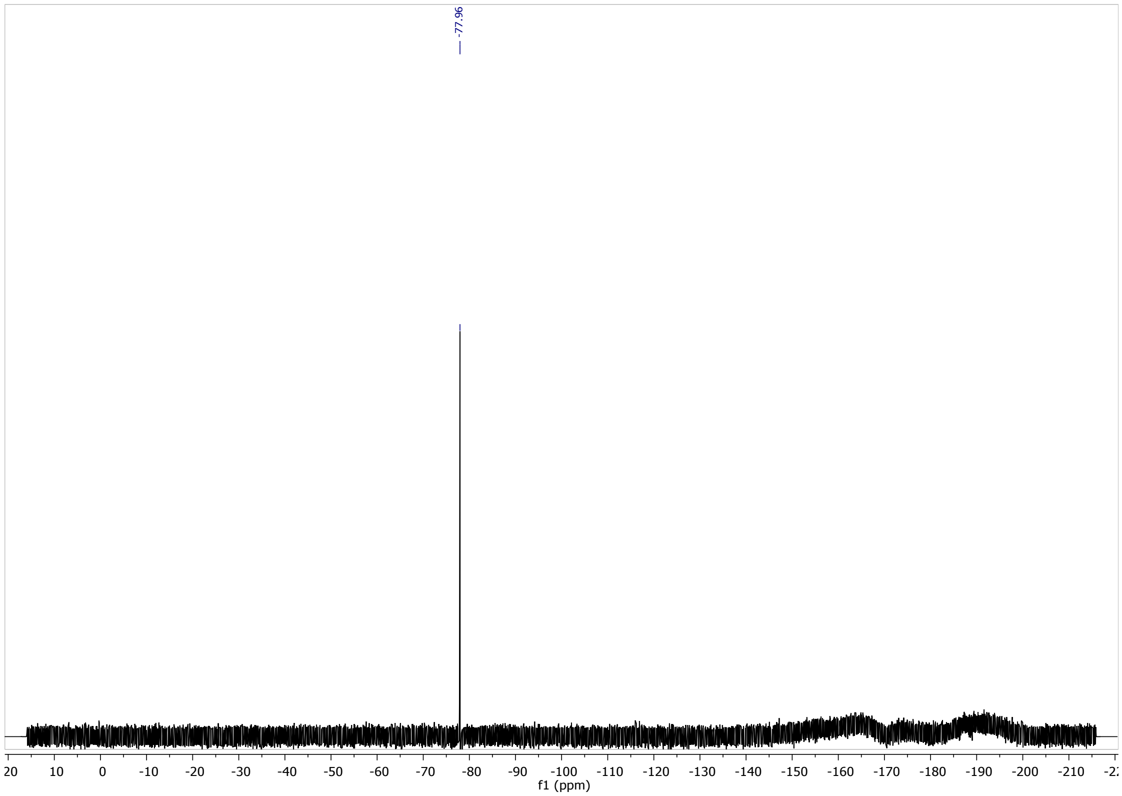

Fig. S25.- ^{19}F NMR spectrum of complex **Cu2b** in $CDCl_3$.

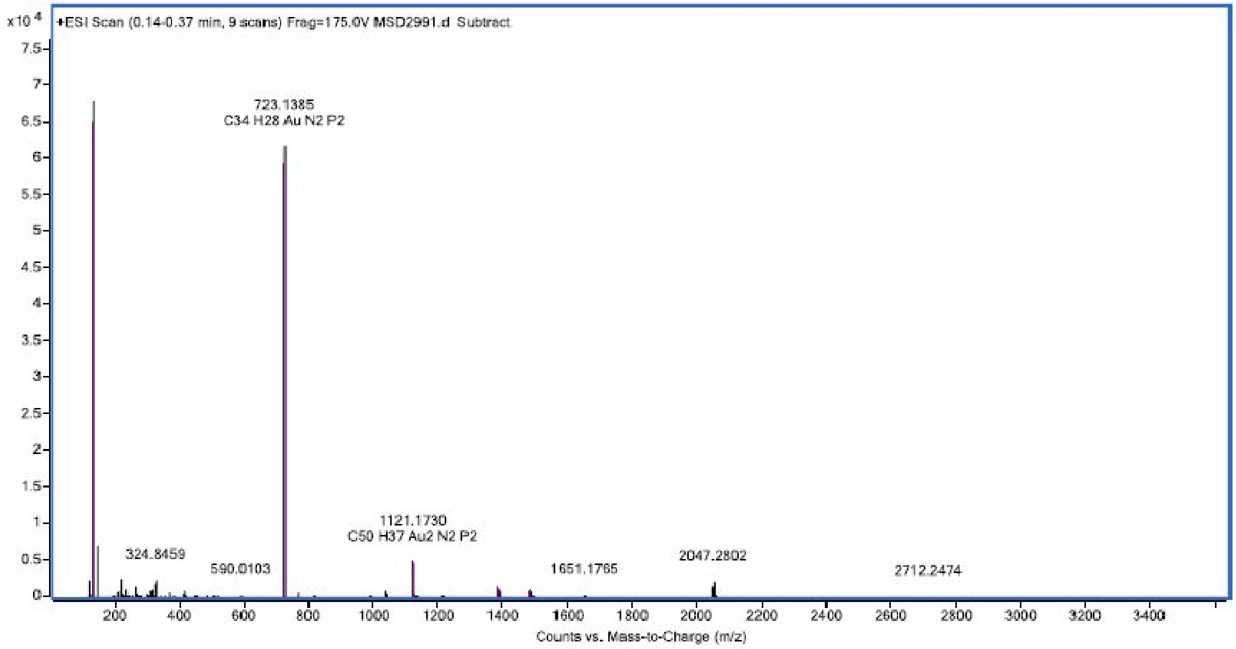

Fig. S26.- ESI-TOF(+) of complex **Cu2b**.

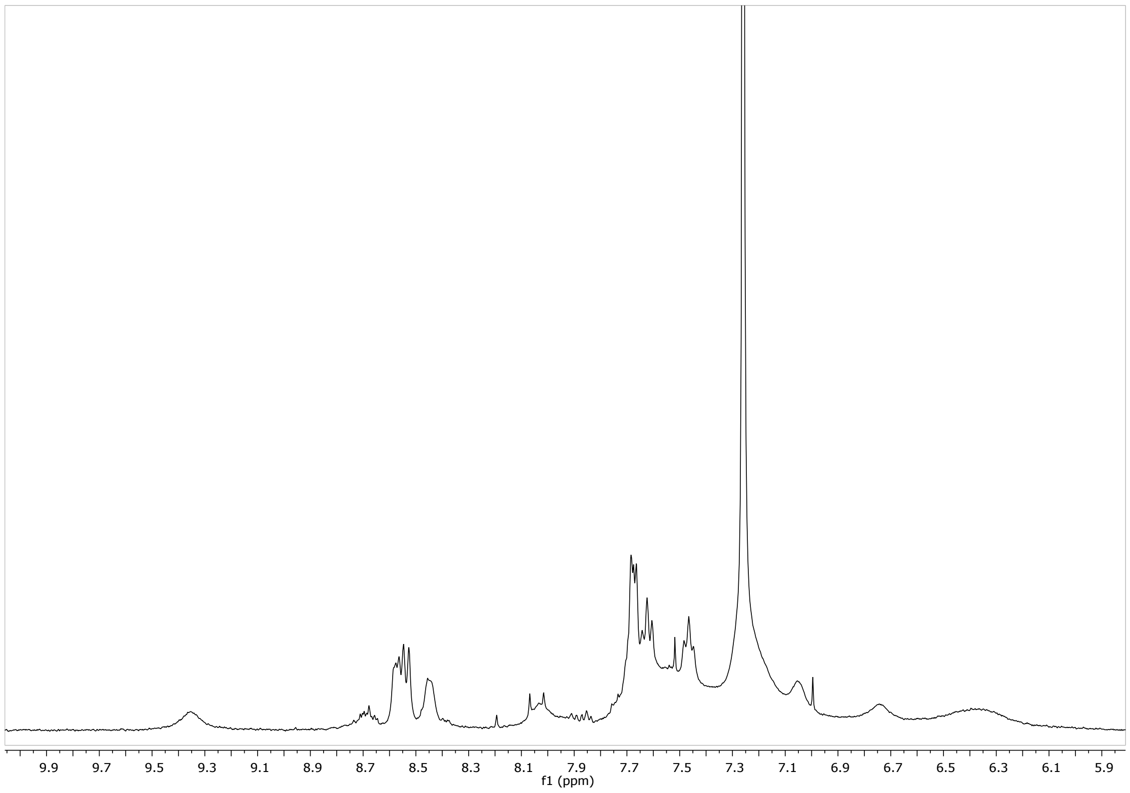

Fig. S27.- ^{1}H NMR spectrum of complex **Cu2c** in $CDCl_3$.

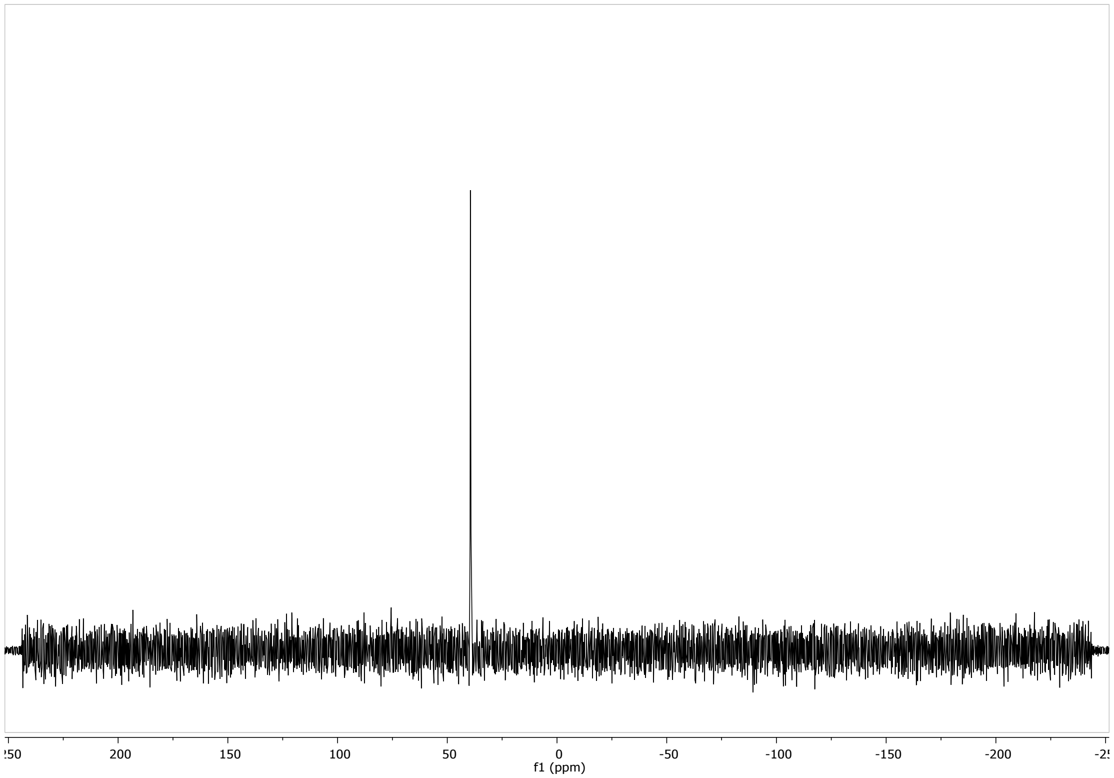

Fig. S28.- ^{31}P NMR spectrum of complex **Cu2c** in $CDCl_3$.

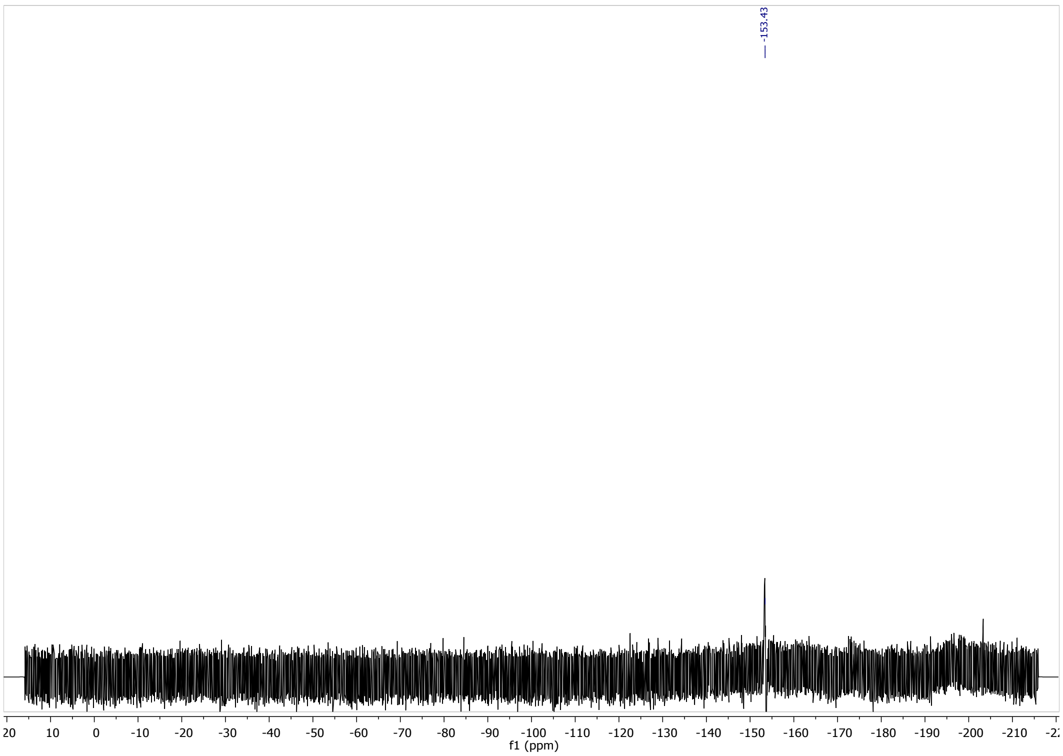

Fig. S29.- ^{19}F NMR spectrum of complex **Cu2c** in $CDCl_3$.

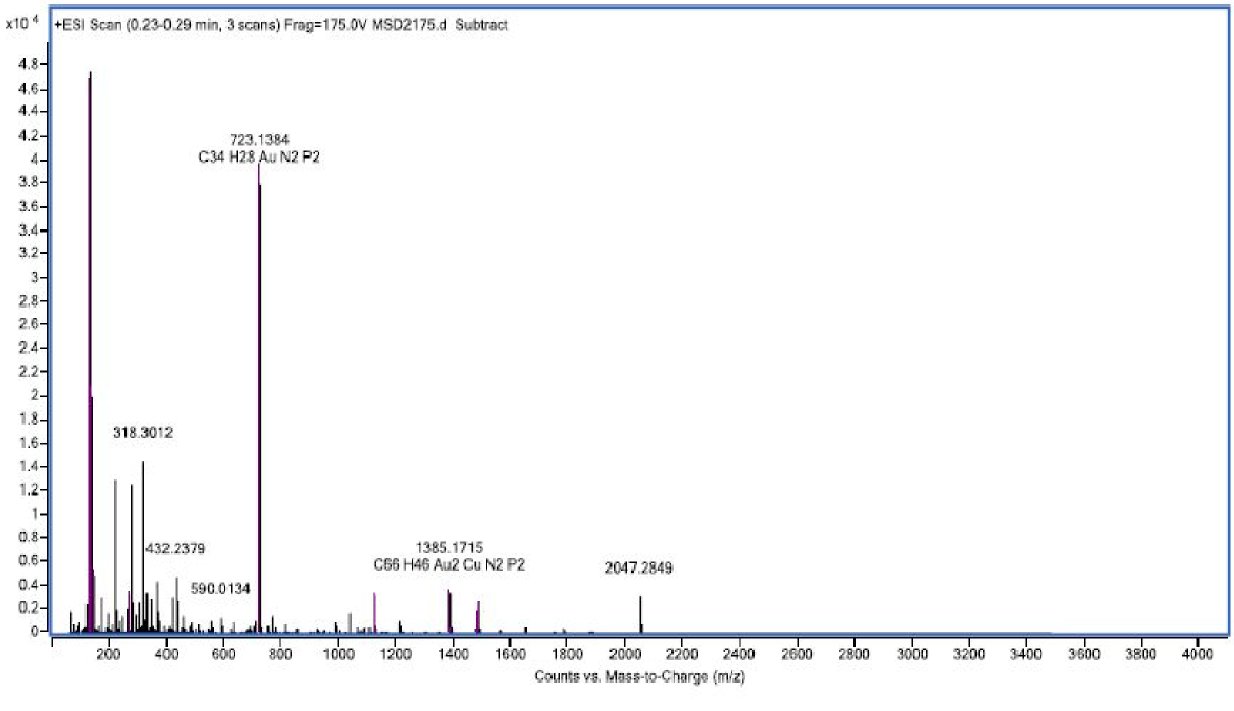

Fig. S30.- ESI-TOF(+) of complex **Cu2c**.

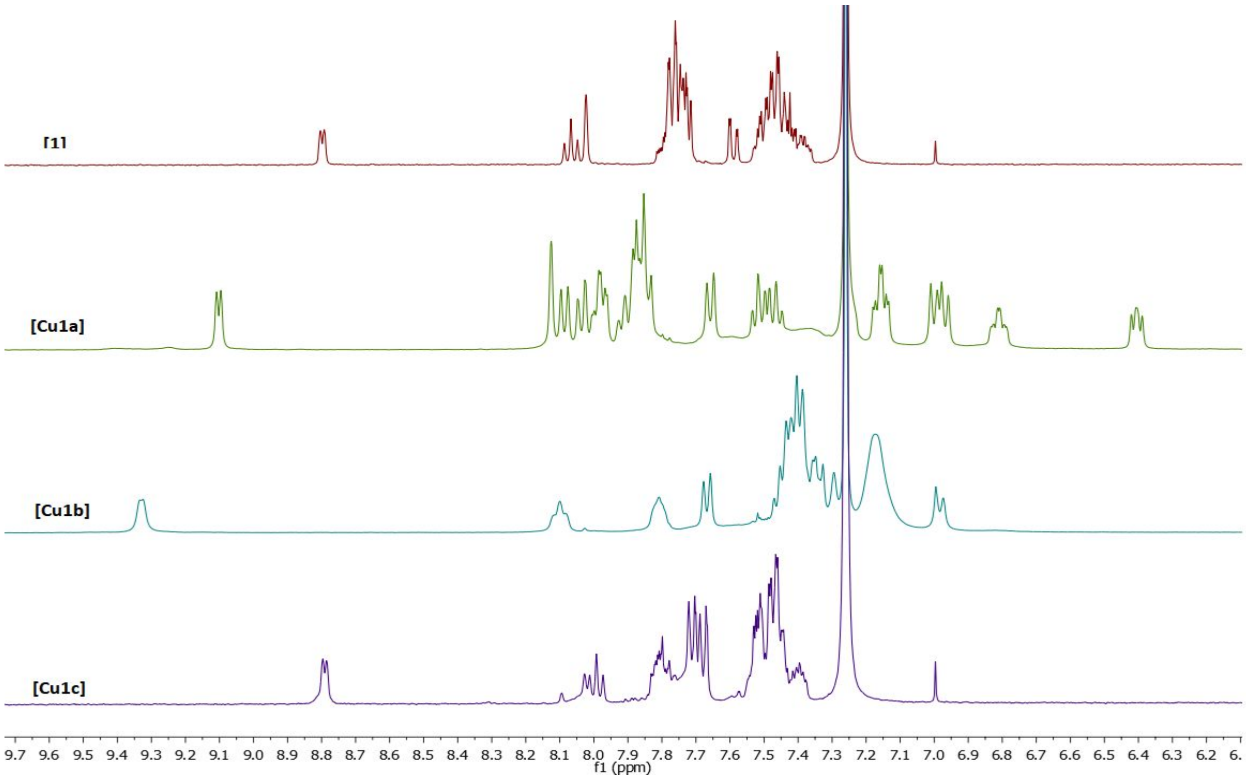

Fig. S31.- Stacked ^{1}H NMR spectra of complexes **1** and **Cu1a-Cu1c** in $CDCl_3$.

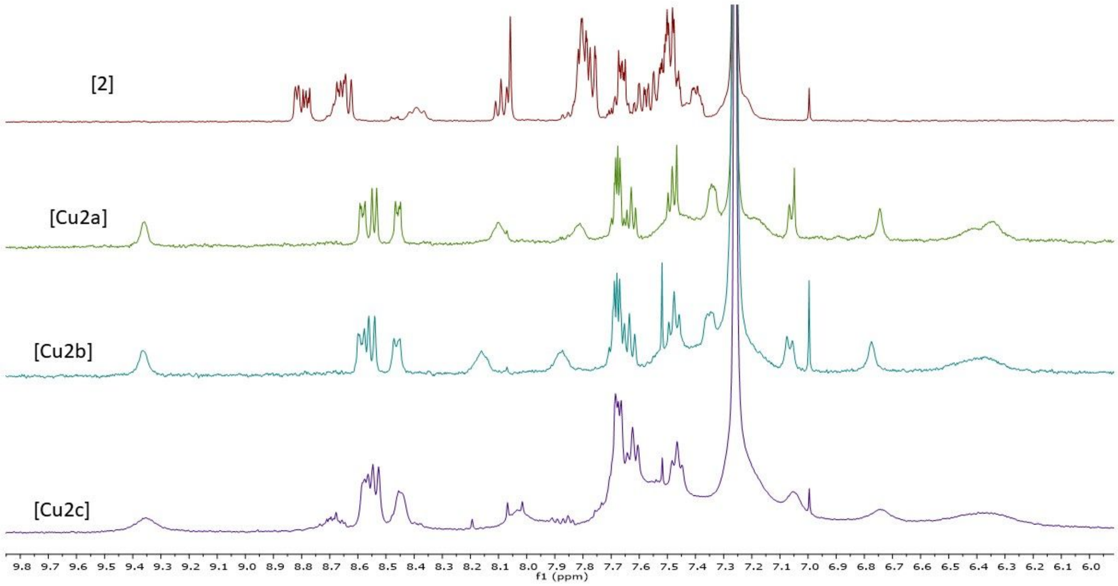

Fig. S32.- Stacked ^{1}H NMR spectra of complexes **2** and **Cu2a-Cu2c** in $CDCl_3$.

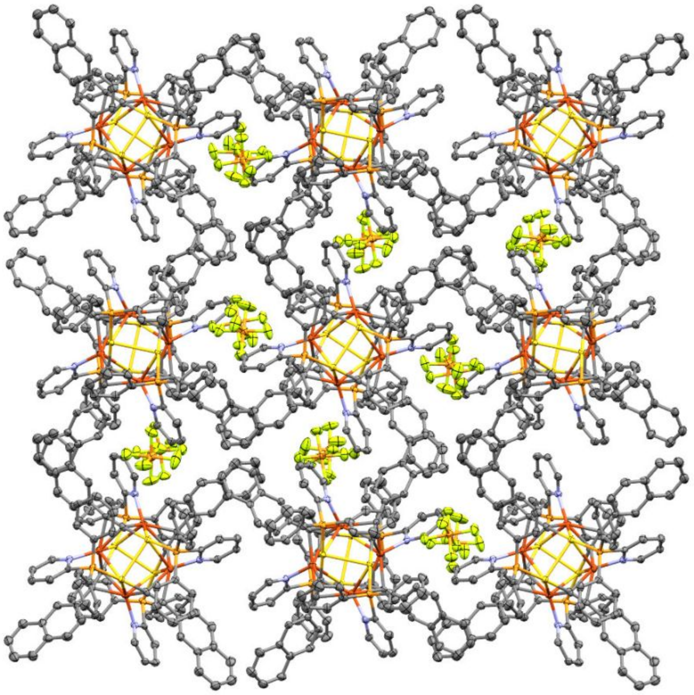

Fig. S33.- Supramolecular arrangement of complex **Cu1a** in the axis *c*. Atoms colors: yellow, gold; bronze, copper; orange, phosphorus; light green, fluorine (thermal parameters at 50% probability; hydrogen atoms have been omitted for clarity).

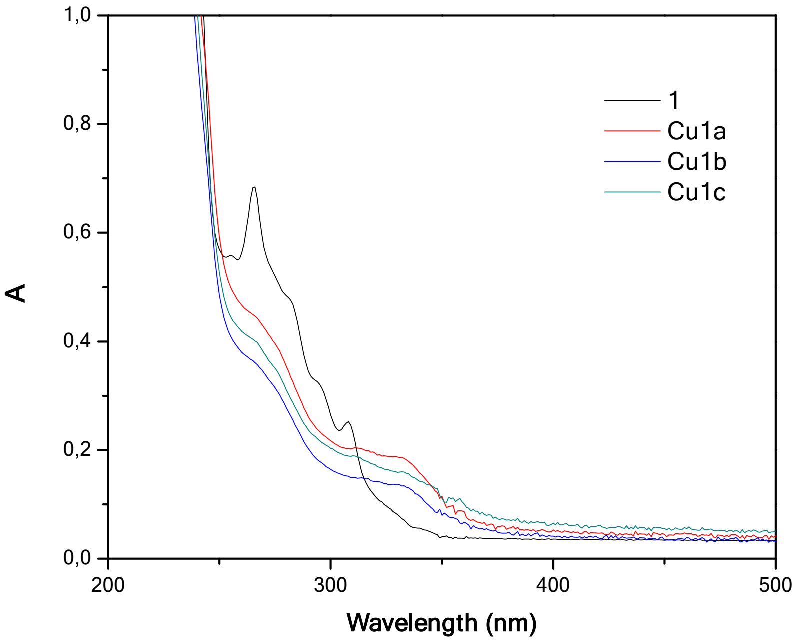

Fig. S34.- Absorption spectra of $\cdot 10^{-5}$ M dichloromethane solutions (air-eq) of compounds **1 -Cu1a–Cu1c**.

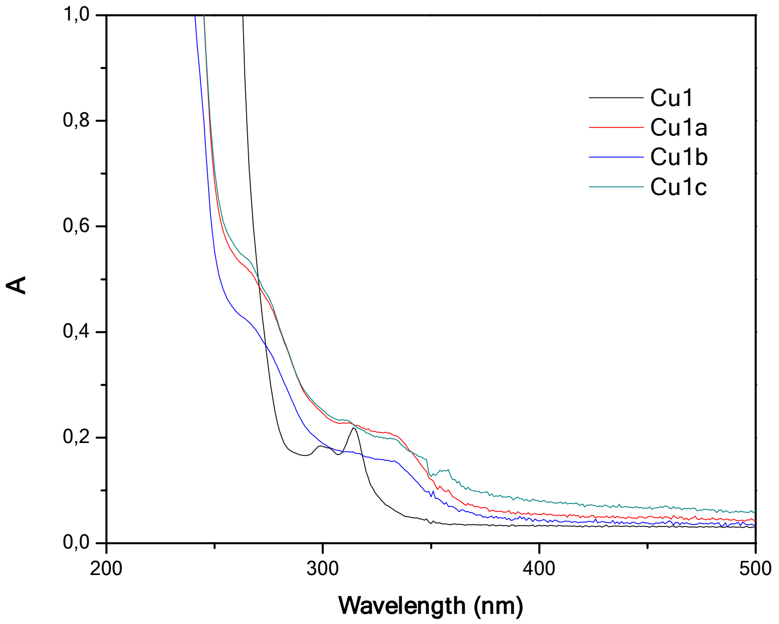

Fig. S35.- Absorption spectra of $\cdot 10^{-5}$ M dichloromethane solutions (N_2 sat) of compounds **1- Cu1a–Cu1c**.

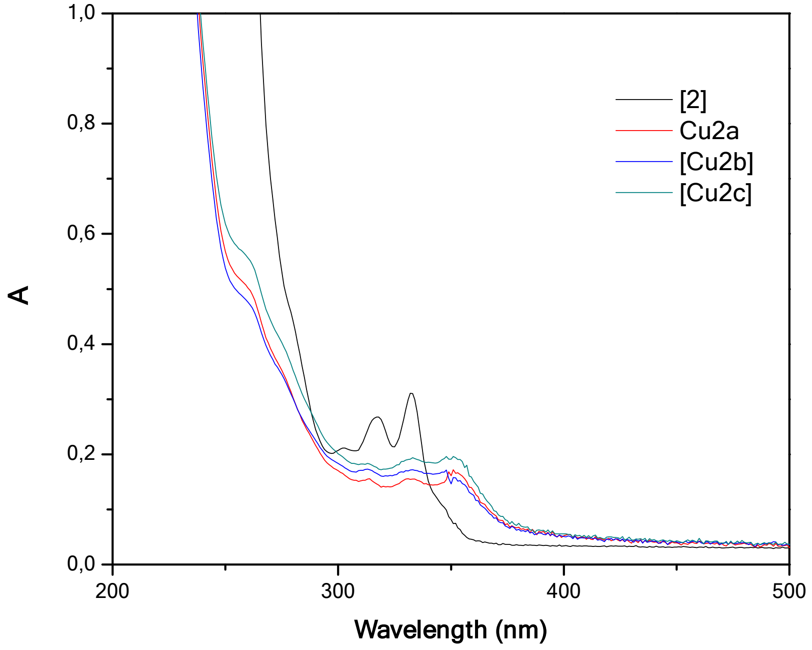

Fig. S36.- Absorption spectra of·10^{-5} M dichloromethane solutions (air-eq) of compounds **2- Cu2a–Cu2c**.

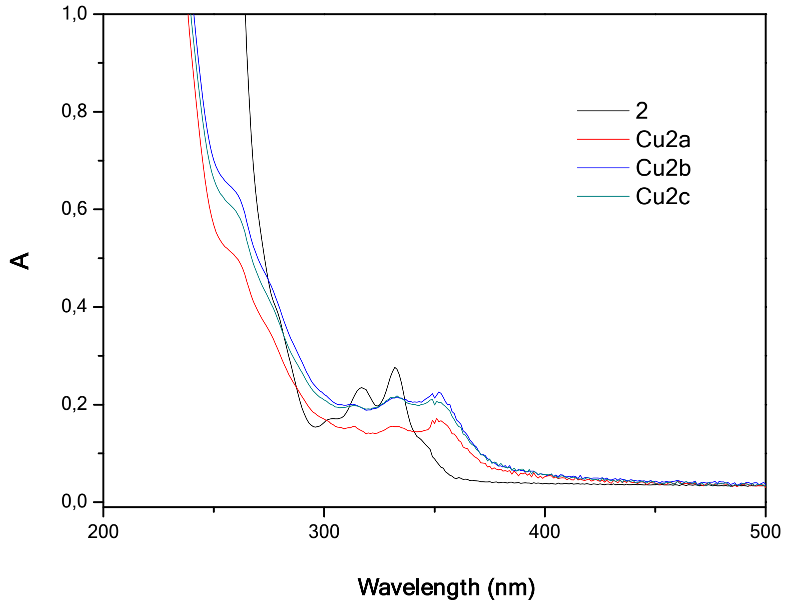

Fig. S37.- Absorption spectra of·10^{-5} M dichloromethane solutions (N_2 sat) of compounds **2- Cu2a–Cu2c**.

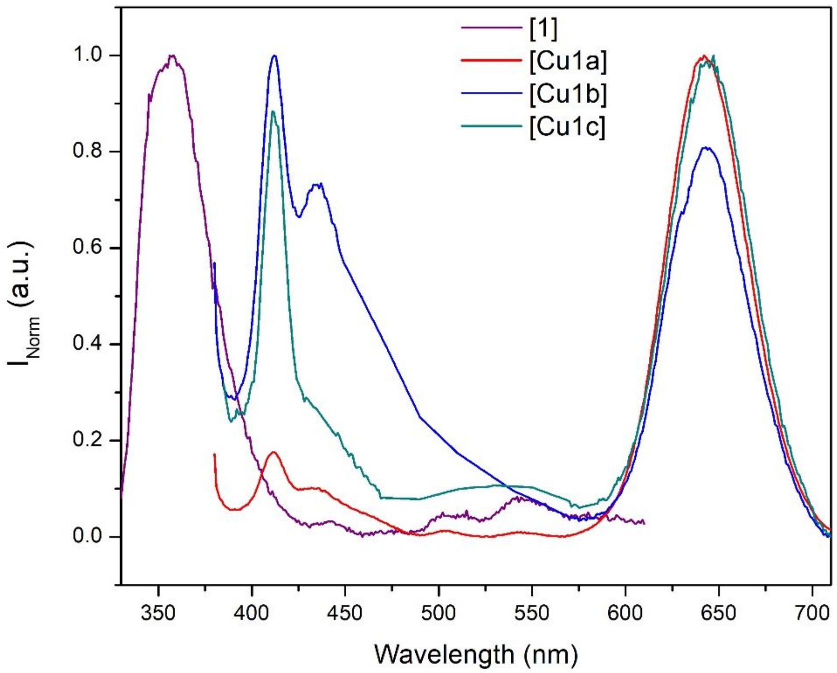

Fig. S38.- Normalized emission spectra of 10^{-5} M dichloromethane solutions (air-eq) of compounds **1 -Cu1a–Cu1c** at λ_{ex}: 350 nm (for bimetallic complexes).

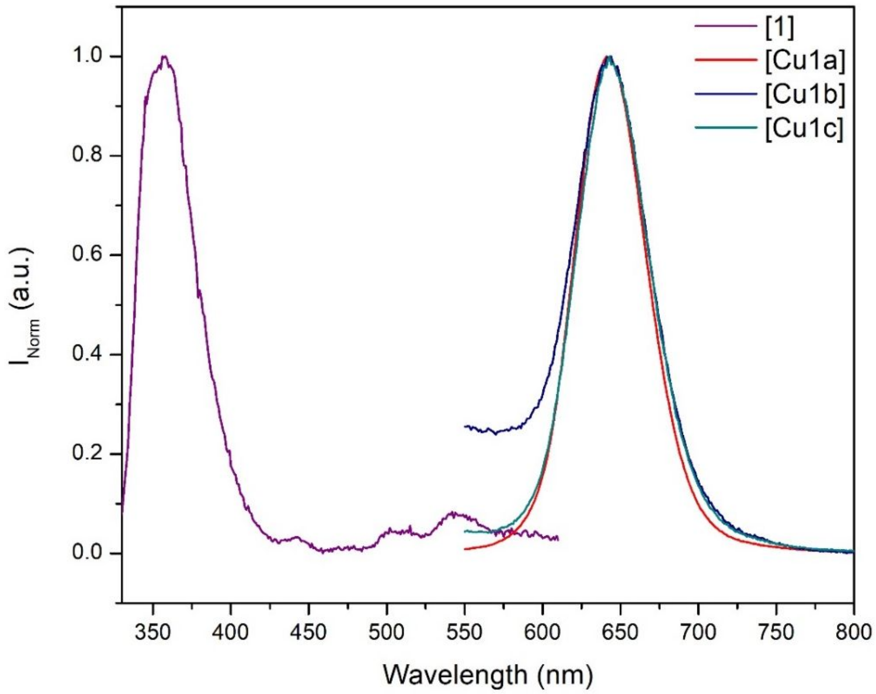

Fig. S39.- Normalized emission spectra of 10^{-5} M dichloromethane solutions (air-eq) of compounds **1 -Cu1a–Cu1c** at λ_{ex}: 450 nm (for bimetallic complexes).

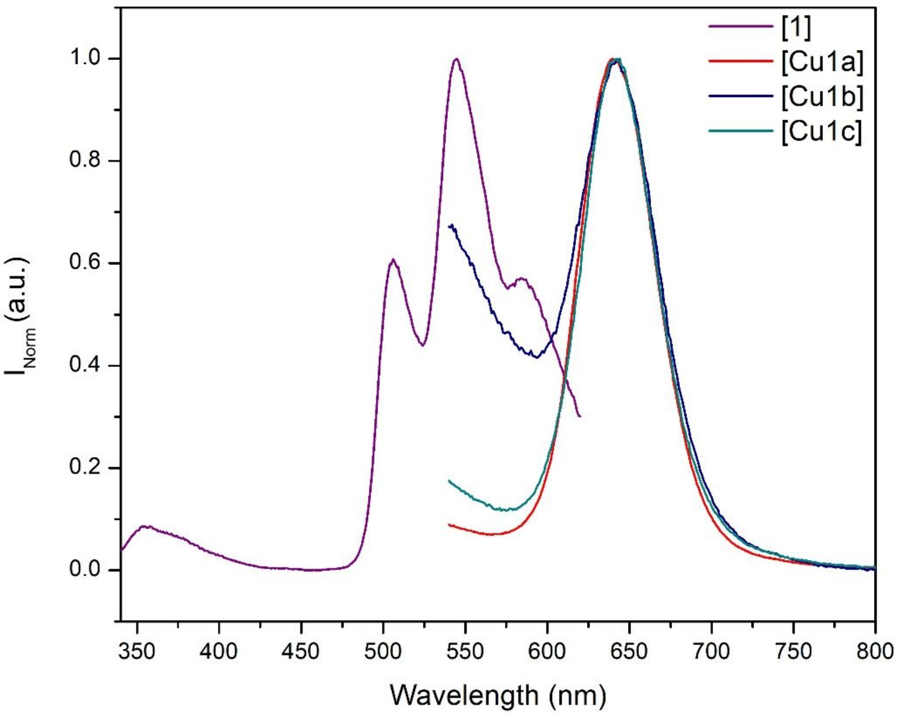

Fig. S40.- Emission spectra of 10^{-5} M dichloromethane solutions (N_2 sat) of compounds **1 - Cu1a–Cu1c** at λ_{ex}:450nm (for bimetallic complexes).

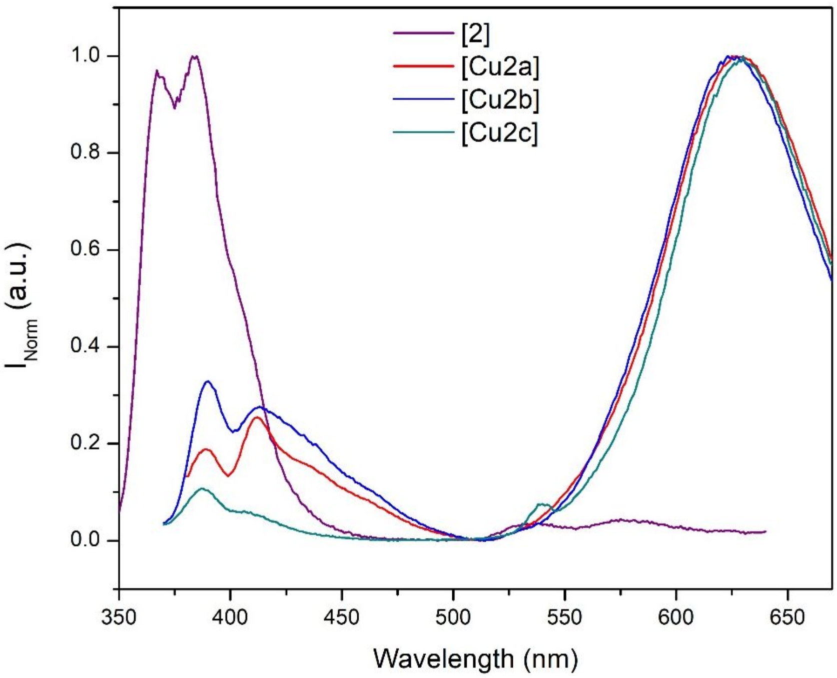

Fig. S41.- Emission spectra of 10^{-5} M dichloromethane solutions (air- eq) of compounds **2 - Cu2a–Cu2c** at λ_{ex}: 350 nm (for bimetallic complexes).

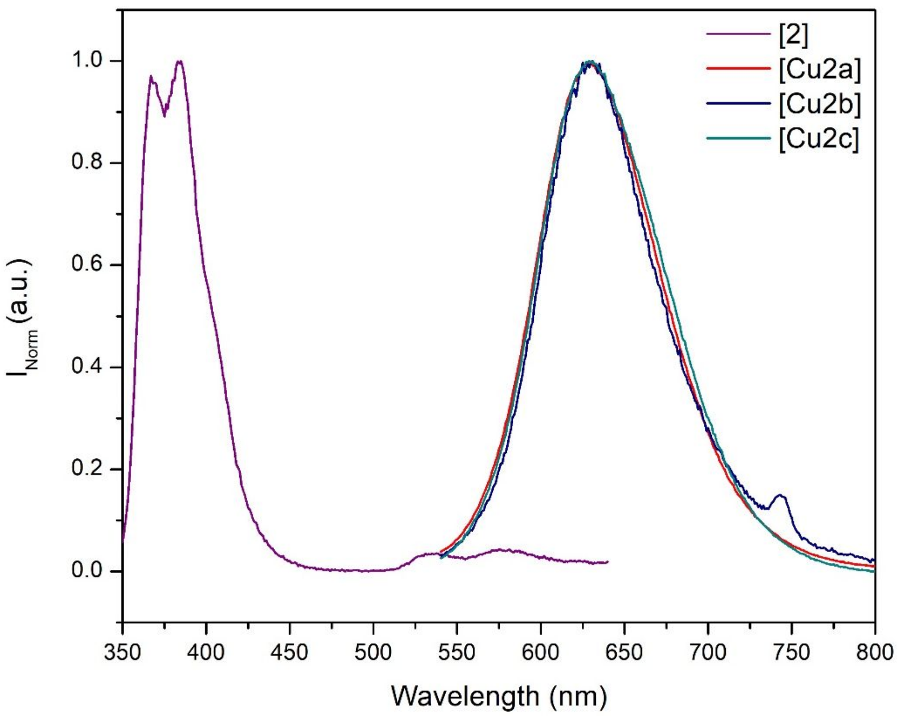

Fig. S42.- Emission spectra of 10^{-5} M dichloromethane solutions (air- eq) of compounds **2 - Cu2a–Cu2c** at λ_{ex}: 450 nm (for bimetallic complexes).

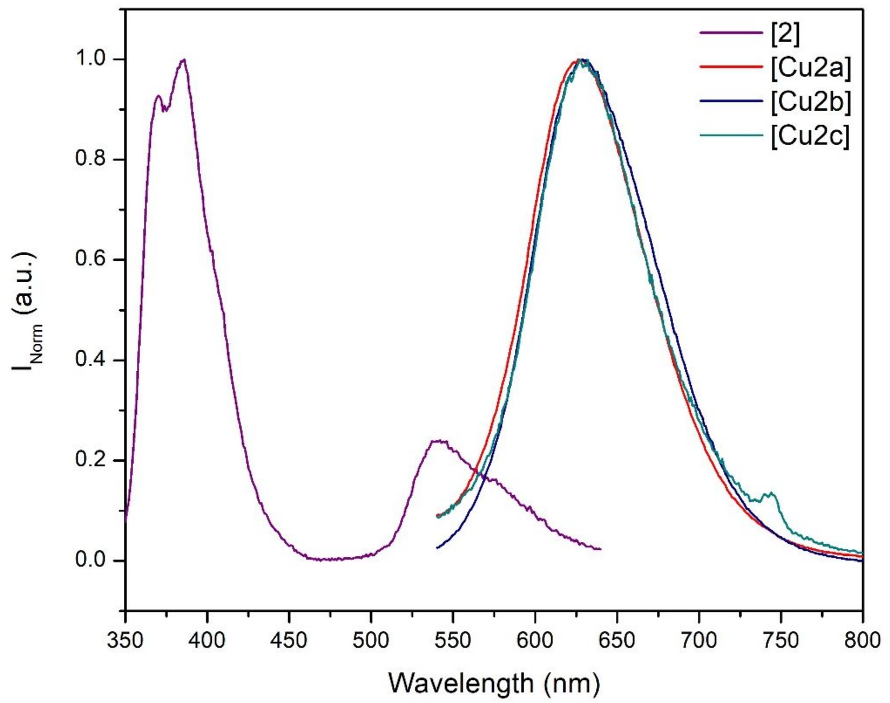

Fig. S43.- Emission spectra of 10^{-5} M dichloromethane solutions (N_2 sat) of compounds **2 - Cu2a–Cu2c** at λ_{ex}: 450nm (for bimetallic complexes).

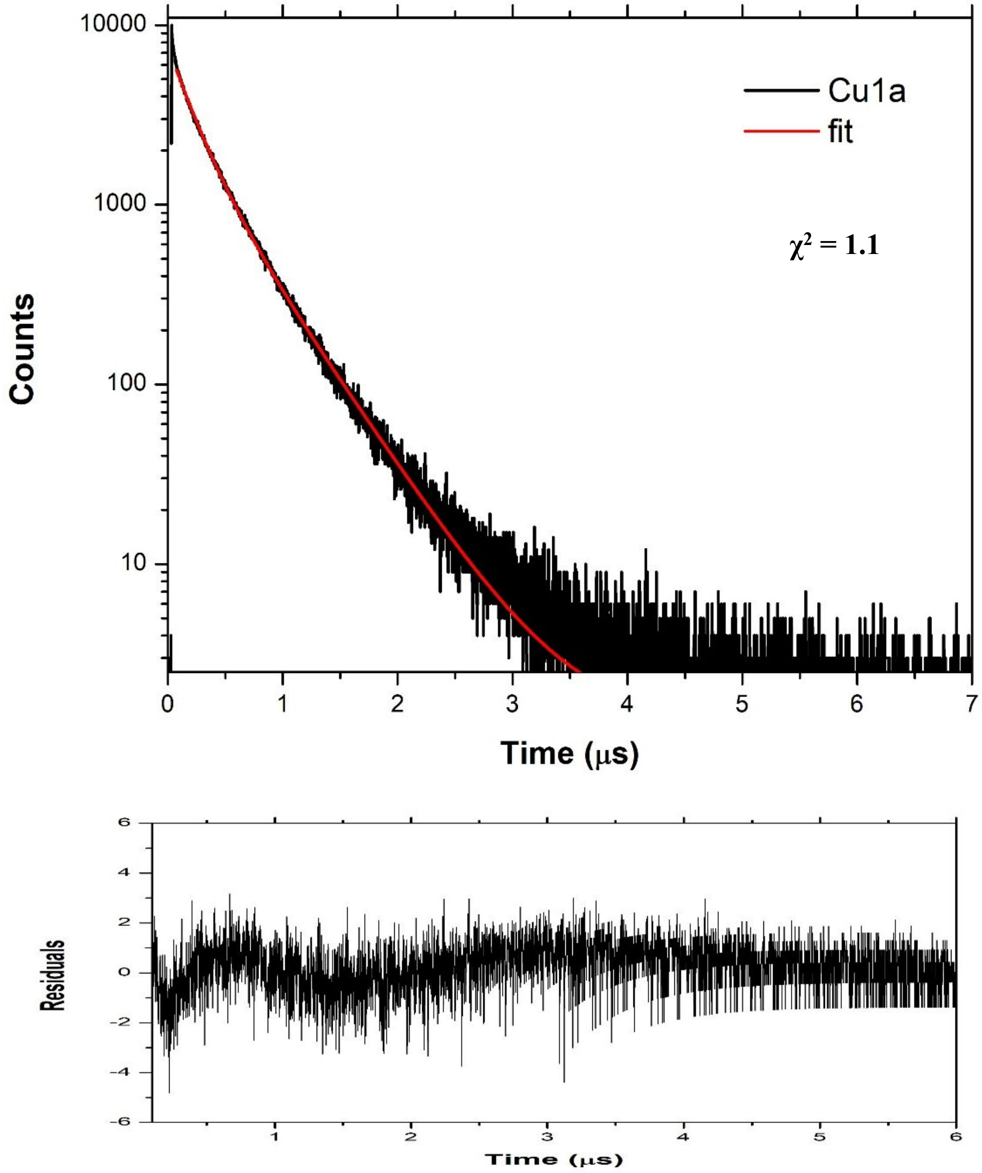

Fig. S44.- Phosphorescence lifetime and residuals of **Cu1a** in dichloromethane solution.

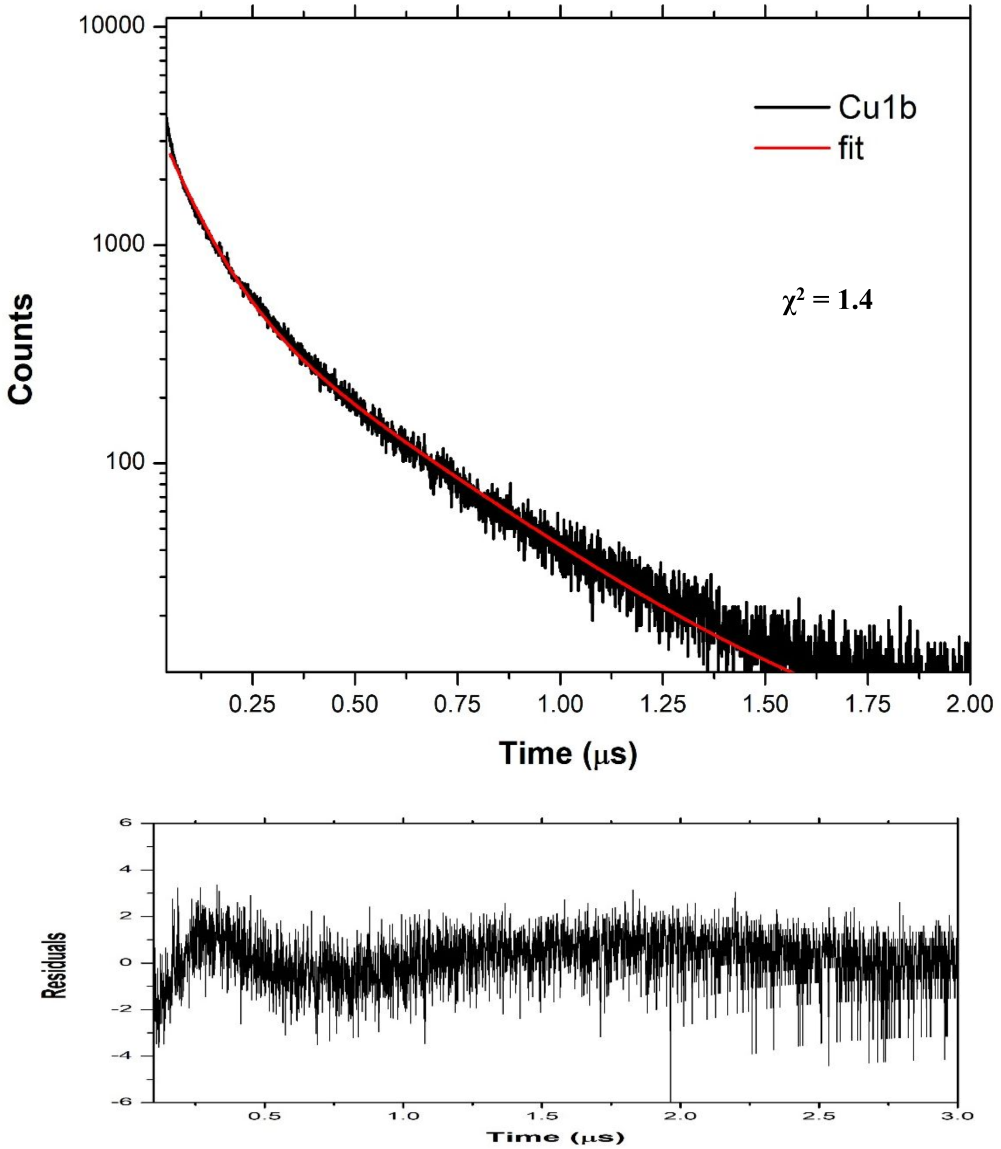

Fig. S45.- Phosphorescence lifetime and residuals of **Cu1b** in dichloromethane solution.

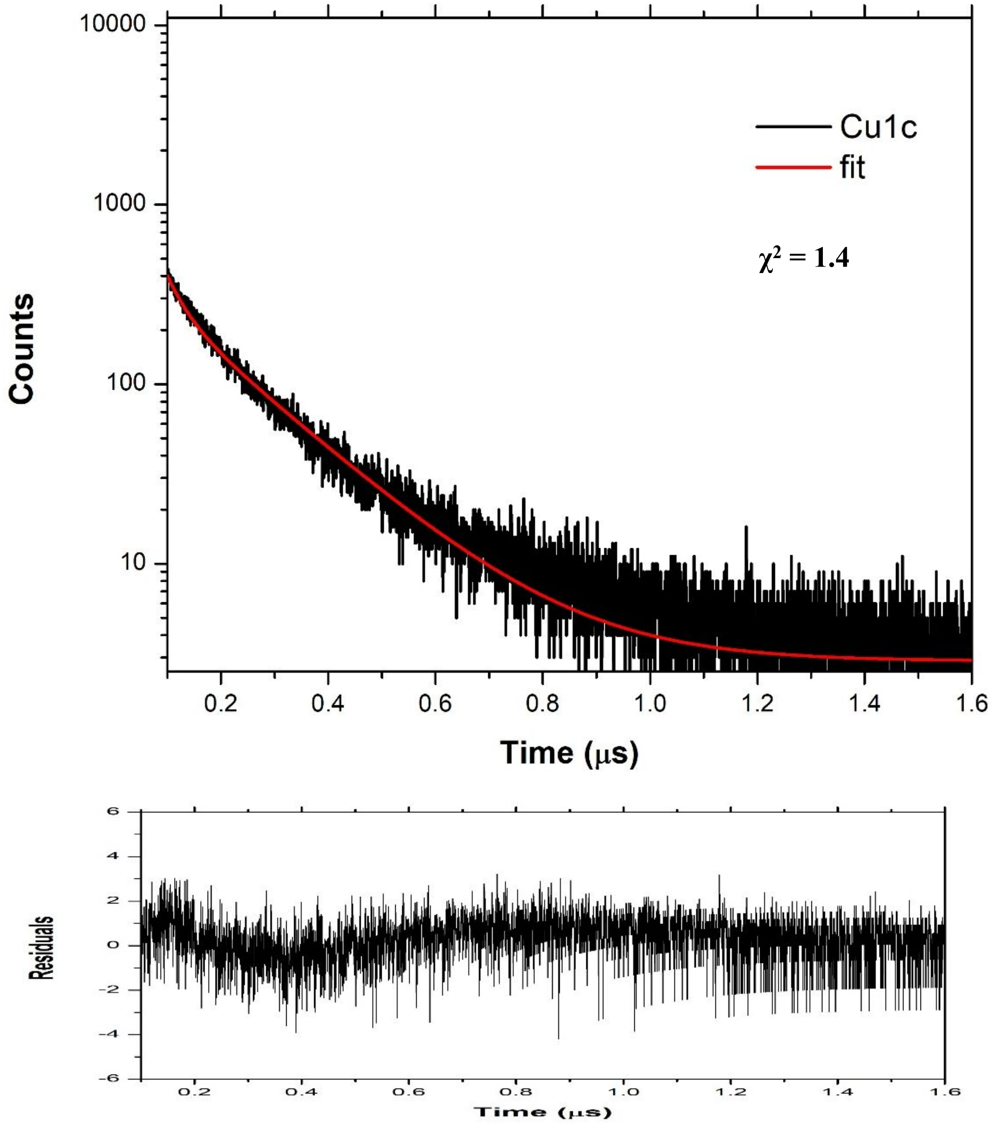

Fig. S46.- Phosphorescence lifetime and residuals of **Cu1c** in dichloromethane solution.

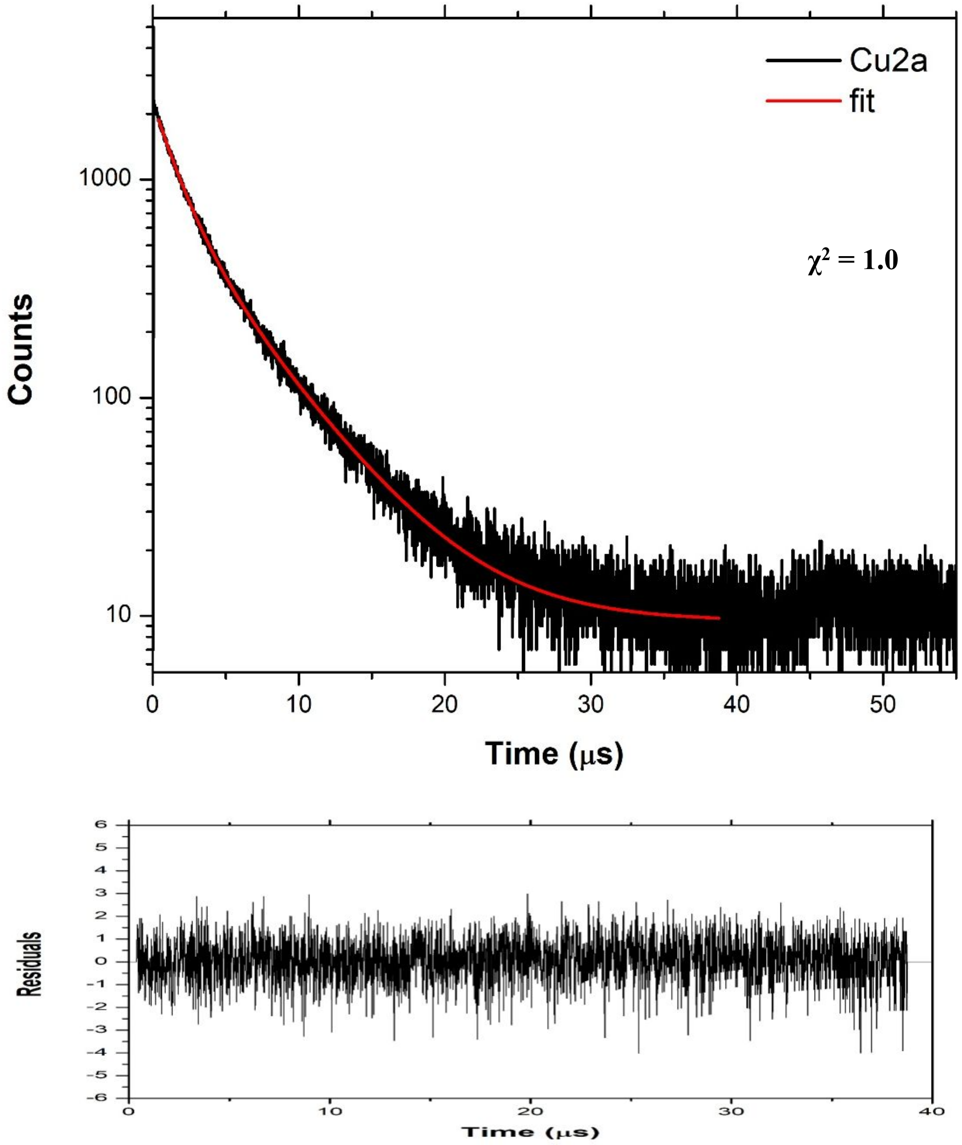

Fig. S47.- Phosphorescence lifetime and residuals of **Cu2a** in dichloromethane solution.

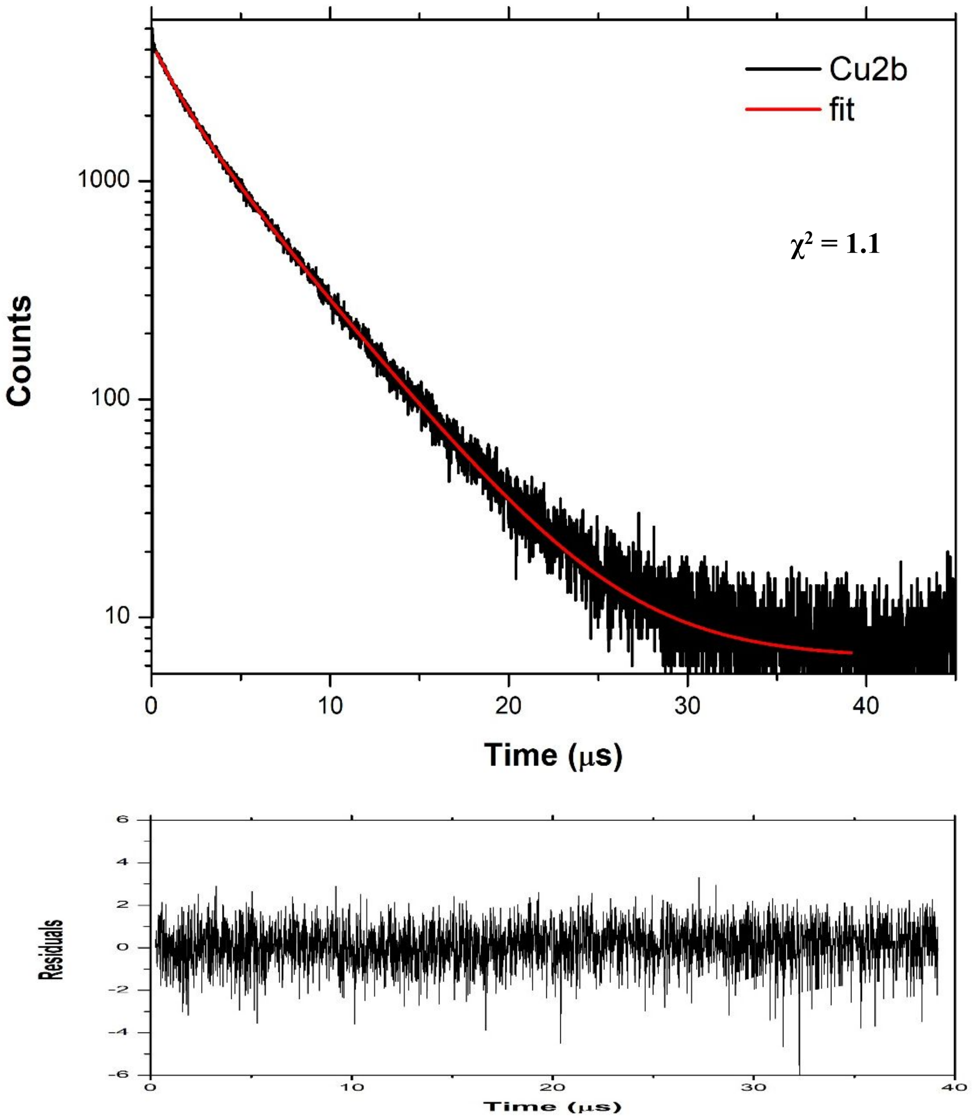

Fig. S48.- Phosphorescence lifetime and residuals of **Cu2b** in dichloromethane solution.

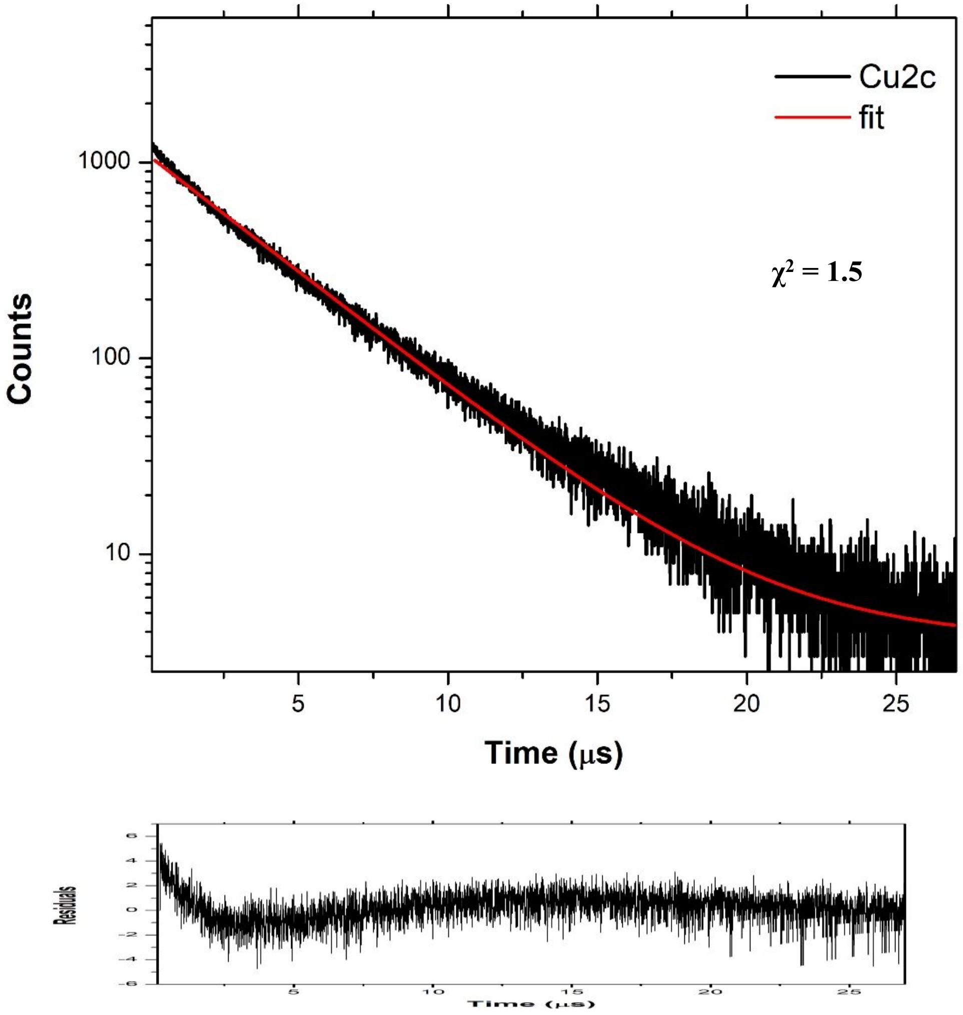

Fig. S49.- Phosphorescence lifetime and residuals of **Cu2c** in dichloromethane solution.

Table S1. Emission maxima values (λ_{max} em) of gold(I) complexes **1** and **2** and their heterometallic Au(I)-Cu(I) complexes in solid-state at different temperatures and supported in polymeric matrixes. FL = Fluorescence; Phos = Phosphorescence.

**Complex**	λ_{max} em Fl/Phos (nm) 298K solid-state	λ_{max} em Fl/Phos (nm) 77K solid-state	λ_{max} em Fl/Phos (nm) 298K (PMMA)	λ_{max} em Fl/Phos (nm) 298K (PS)
**1**	-/-	-/-	370/506,546	372/505, 547
**2**	-/-	-/-	412/539, 582	389/538, 578
**Cu1a**	-/641	-/673	-/641	-/651
**Cu1b**	-/640	-/678	-/648	-/646
**Cu1c**	-/643	-/672	-/657	-/661
**Cu2a**	-/651	-/658	-/606	-/626
**Cu2b**	-/661	-/644, 694	-/620	-/625
**Cu2c**	-/620	-/656	-/607	-/637

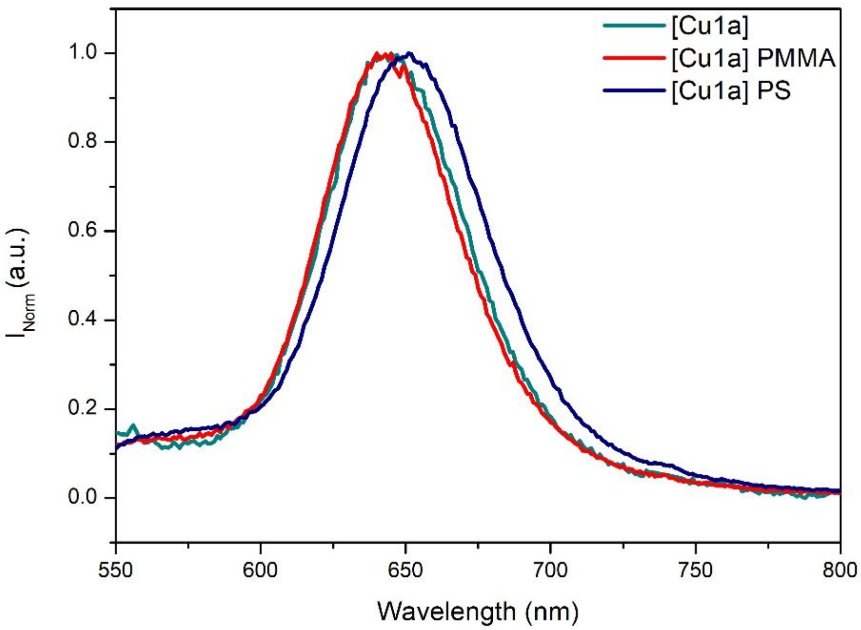

Fig. S50.- Normalized emission spectra for compounds **Cu1a** complexes in solid-state and supported in polymeric solid matrixes PMMA and PS at λ_{ex}: 450 nm.

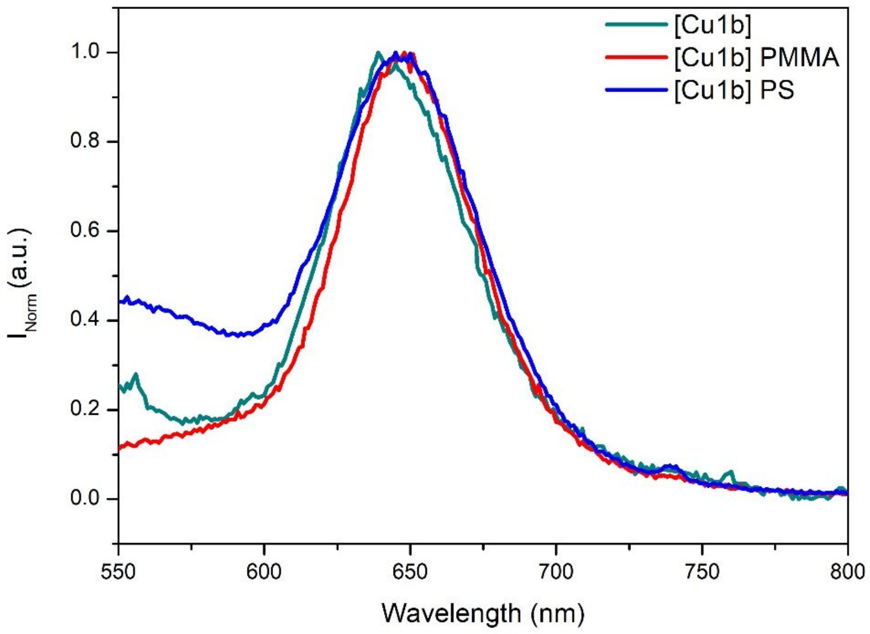

Fig. S51.- Normalized emission spectra for compounds **Cu1b** complexes in solid-state and supported in polymeric solid matrixes PMMA and PS at λ_{ex}: 450 nm.

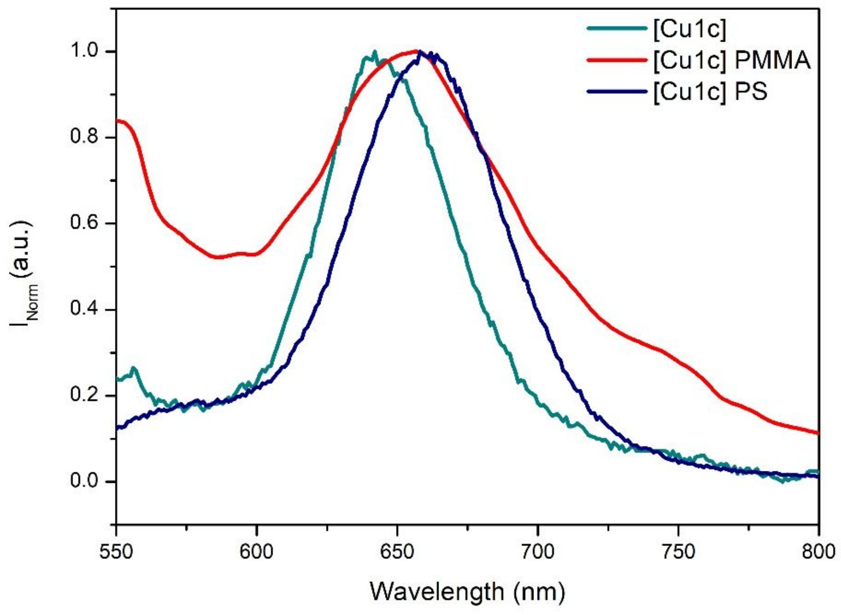

Fig. S52.- Normalized emission spectra for compounds **Cu1c** complexes in solid-state and supported in polymeric solid matrixes PMMA and PS at λ_{ex}: 450 nm.

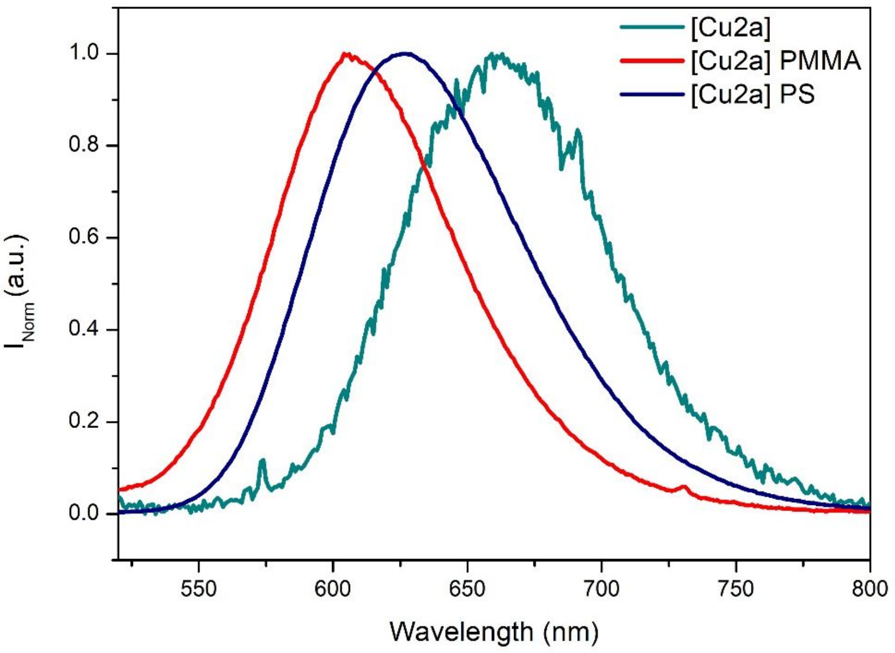

Fig. S53.- Normalized emission spectra for compounds **Cu2a** complexes in solid-state and supported in polymeric solid matrixes PMMA and PS at λ_{ex}: 450 nm.

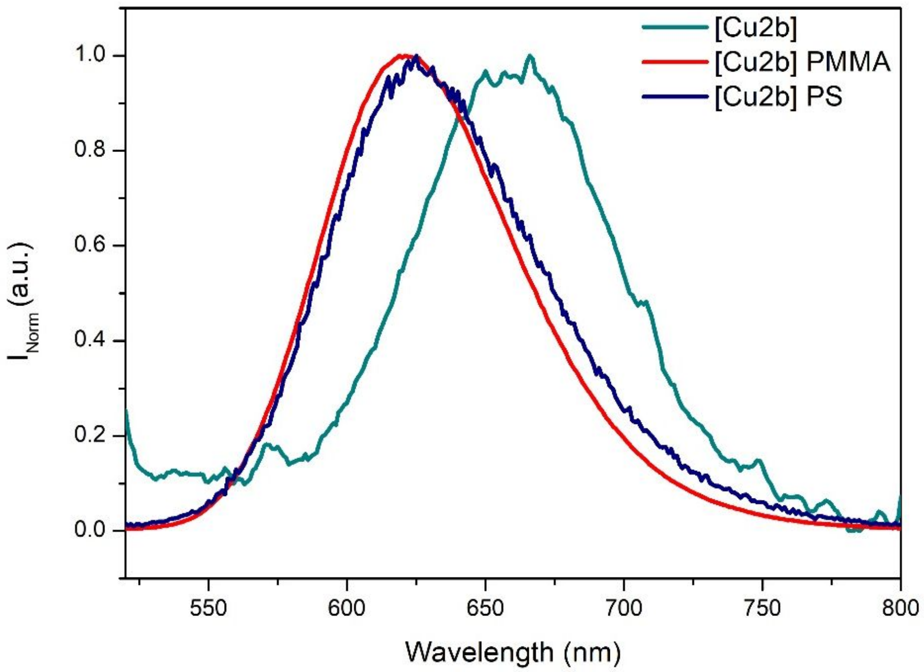

Fig. S54.- Normalized emission spectra for compounds **Cu2b** complexes in solid-state and supported in polymeric solid matrixes PMMA and PS at λ_{ex}: 450 nm.

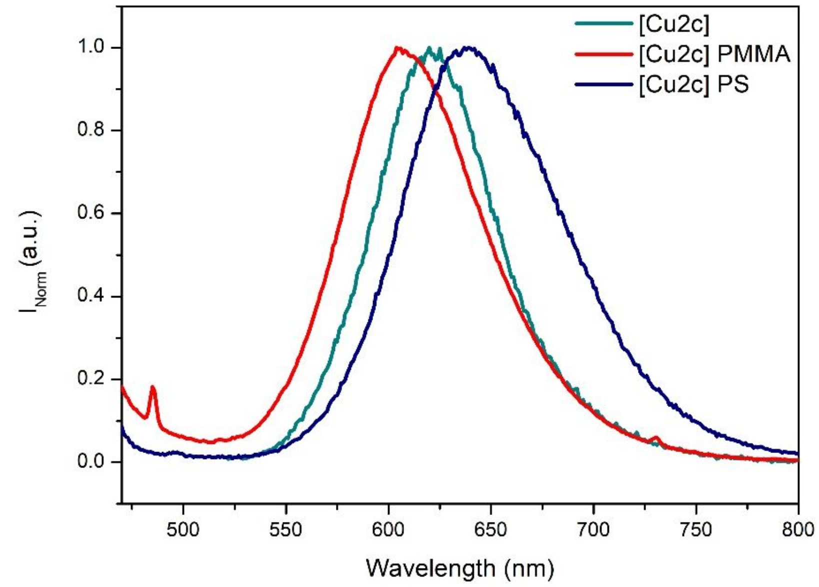

Fig. S55.- Normalized emission spectra for compounds **Cu2c** complexes in solid-state and supported in polymeric solid matrixes PMMA and PS at λ_{ex}: 450 nm.

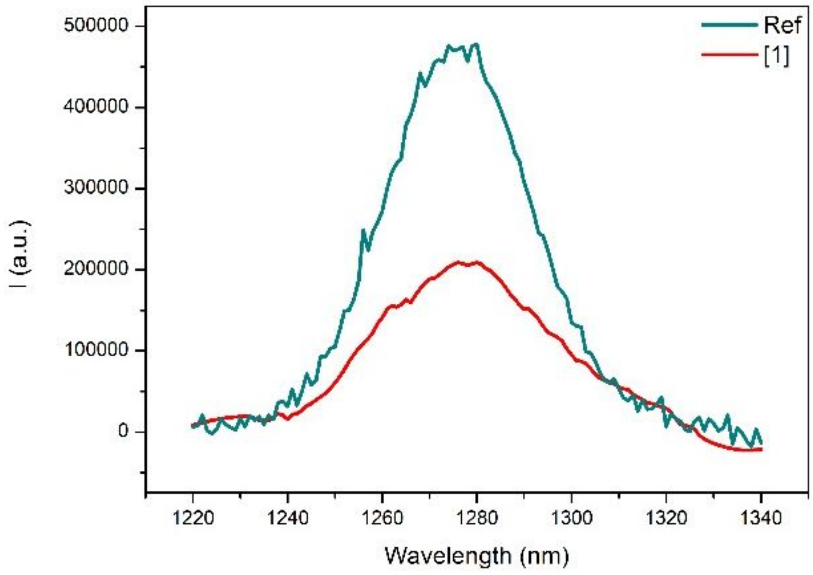

Fig. S56.- $^{1}O_2$ production of gold(I) complexes **1** in acetonitrile air-equilibrated solutions at λ_{ex}: 380nm.

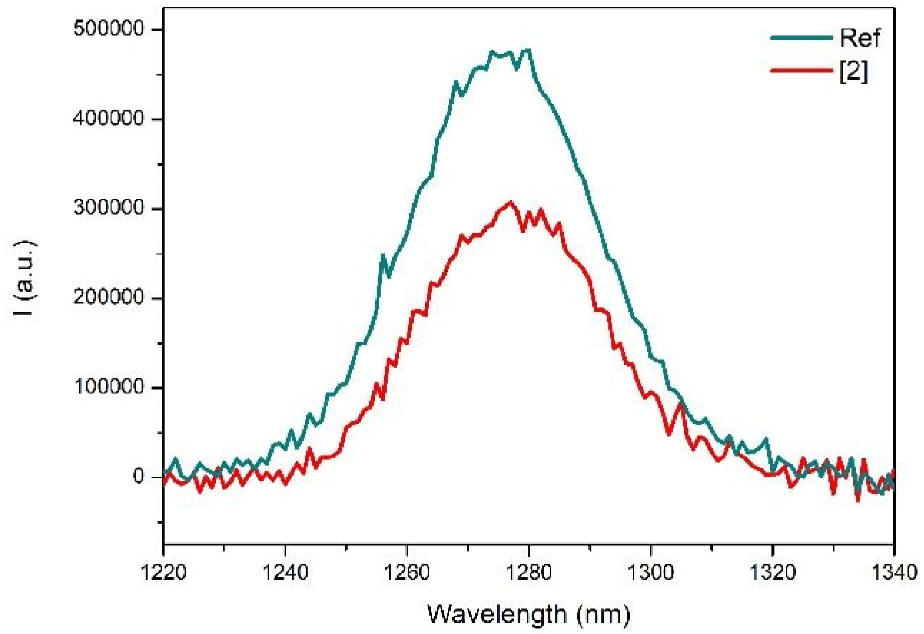

Fig. S57.- $^{1}O_2$ production of gold(I) complexes **2** in acetonitrile air-equilibrated solutions at λ_{ex}: 380nm.

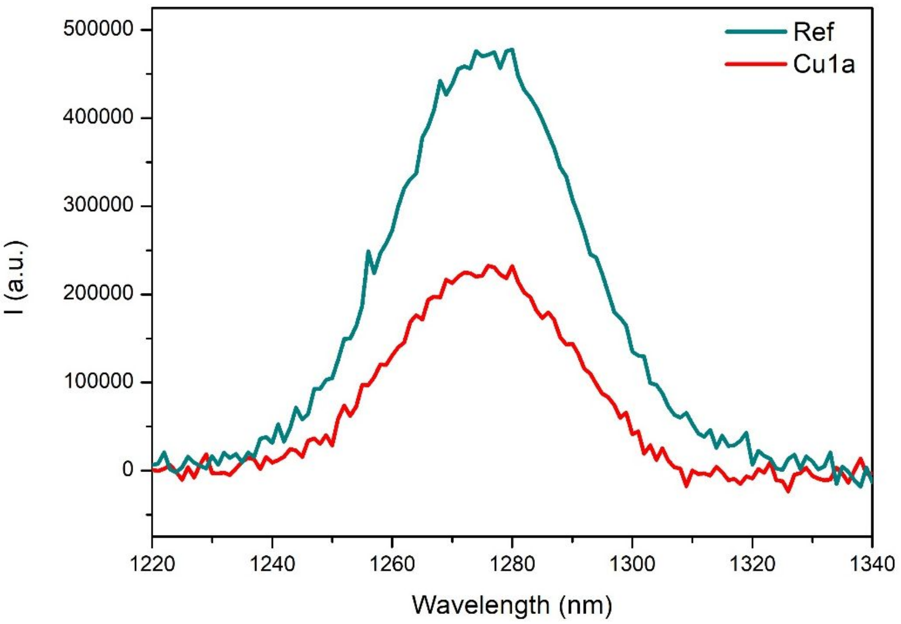

Fig. S58.- $^{1}O_2$ production of gold(I) complexes **Cu1a** in acetonitrile air-equilibrated solutions at λ_{ex}: 380nm.

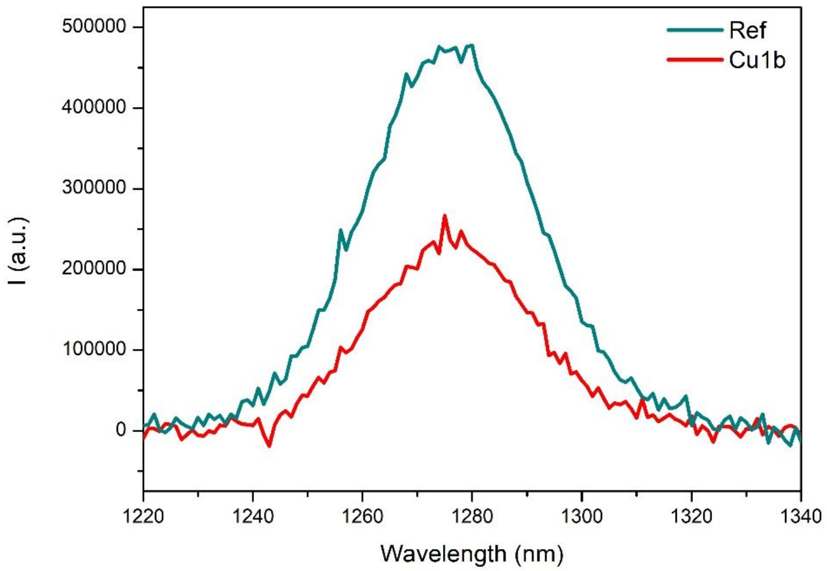

Fig. S59.- $^1O_2$ production of gold(I) complexes **Cu1b** in acetonitrile air-equilibrated solutions at λ_{ex}: 380nm.

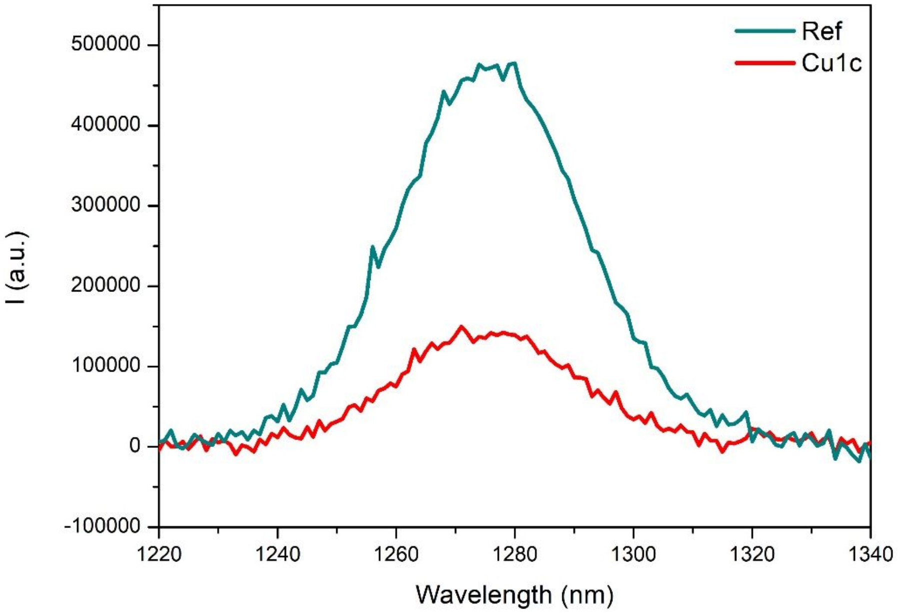

Fig. S60.- $^1O_2$ production of gold(I) complexes **Cu1c** in acetonitrile air-equilibrated solutions at λ_{ex}: 380nm.

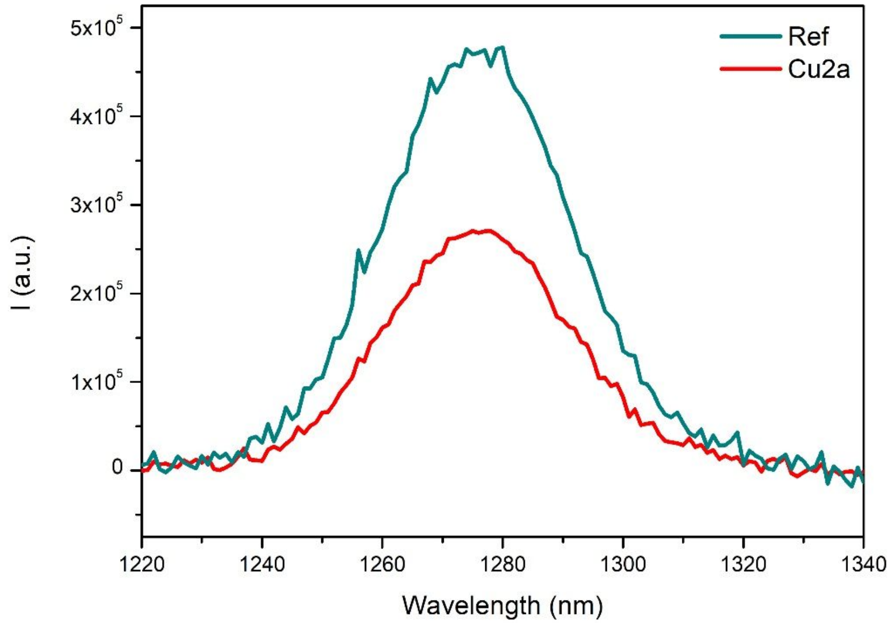

Fig. S61.- $^1O_2$ production of gold(I) complexes **Cu2a** in acetonitrile air-equilibrated solutions at λ_{ex}: 380nm.

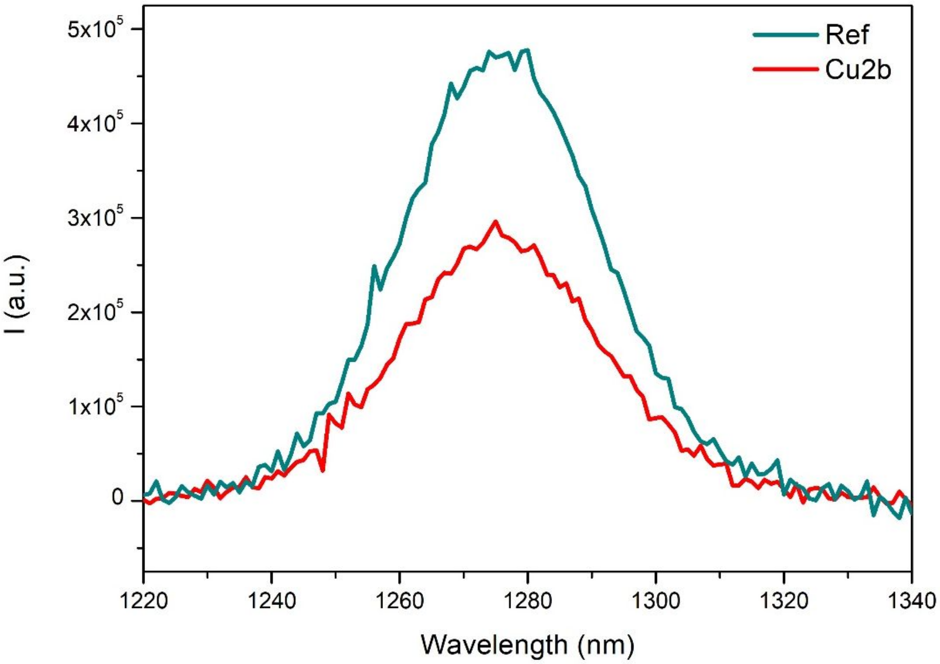

Fig. S62.- $^1O_2$ production of gold(I) complexes **Cu2b** in acetonitrile air-equilibrated solutions at λ_{ex}: 380nm.

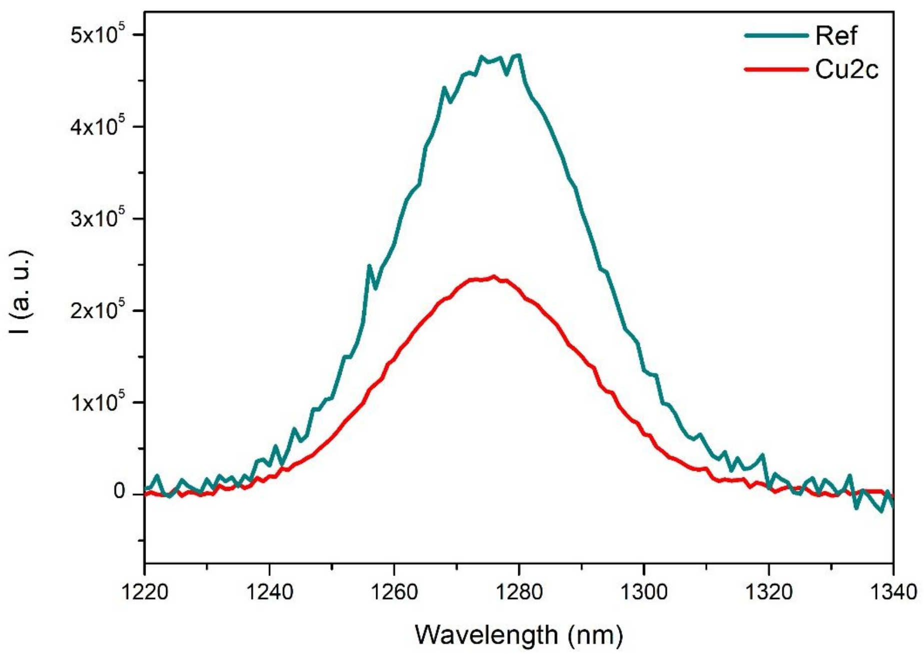

Fig. S63.- $^1O_2$ production of gold(I) complexes **Cu2c** in acetonitrile air-equilibrated solutions at λ_{ex}: 380nm.

Table S2. Crystal data and structure refinement for **Cu1a**.

Compound	Cu1a
Formula	$[C_{116}H_{84}Au_4Cu_4N_4P_4][PF_6]_4$
Crystal size, mm	0.25 x 0.157 x 0.062
Fw	3279.65
Temp., K	120.0(1)
Wavelength, Å	0.71073
Crystal system	Tetragonal
Space group	*I*-4
a, Å	26.07960(10)
b, Å	26.07960(10)
c, Å	16.19220(10)
α, °	90
β, °	90
γ, °	90
Volume, Å^3	11013.05(11)
Z	4
$D_{calc.}$, mg m^{-3}	1.978
Abs. coef., mm^{-1}	6.273
F(000)	6304
θ range for data coll, °	2.2 to 27.1
Refins coll./independent	93939/12149
Data/restraint/parameters	12149/0/722
GOF on F^2	1.05
Final R index (I > 2σ(I))	R_1 = 0.0233, wR_2 = 0.0511
R index (all data)	R_1 = 0.0285, wR_2 = 0.0536
Peak and hole, e Å^{-3}	1.45/-0.75
CCDC	2234334

Table S3. Crystal data and structure refinement for **Cu2a**.

**Compound**	Cu2a
**Formula**	$[C_{132}H_{92}Au_4Cl_2Cu_4N_4P_4][PF_6]_2$
**Crystal size, mm**	0.148 x 0.098 x 0.046
**Fw**	3260.84
**Temp., K**	120.0(1)
**Wavelength, Å**	0.71073
**Crystal system**	Tetragonal
**Space group**	$I4_1/a$
**a, Å**	19.7731(2)
**b, Å**	19.7731(2)
**c, Å**	65.4074(11)
**α, °**	90
**β, °**	90
**γ, °**	90
**Volume, Å^3**	25572.7(7)
**Z**	8
**$D_{calc.}$, mg m^{-3}**	1.694
**Abs. coef., mm^{-1}**	5.41
**F(000)**	12608
**θ range for data coll, °**	2.3 to 27.1
**Reflns coll./independent**	107592/14075
**Data/restraint/parameters**	14075/198/735
**GOF on F^2**	1.02
**Final R index (I > 2σ(I))**	R_1 = 0.0498,<br>wR_2 = 0.0992
**R index (all data)**	R_1 = 0.0747,<br>wR_2 = 0.1088
**Peak and hole, e Å^{-3}**	3.52/-3.48
**CCDC**	2234335

Table S4. Crystal data and structure refinement for **Cu2c**.

**Compound**	Cu2c
**Formula**	$[C_{132}H_{92}Au_4Cl_2Cu_4N_4P_4][BF_4]_2\cdot H_2O$
**Crystal size, mm**	0.124 x 0.076 x 0.029
**Fw**	3162.53
**Temp., K**	120.0(1)
**Wavelength, Å**	1.54184
**Crystal system**	Triclinic
**Space group**	*P*-1
**a, Å**	14.8726(3)
**b, Å**	16.9720(4)
**c, Å**	24.2848(6)
**α, °**	85.789(2)
**β, °**	83.592(2)
**γ, °**	84.499(2)
**Volume, Å^3**	6051.4(2)
**Z**	2
**D_{calc}., mg m^{-3}**	1.736
**Abs. coef., mm^{-1}**	11.054
**F(000)**	3060
**θ range for data coll, °**	2.6 to 72.1
**Reflns coll./independent**	71141/23710
**Data/restraint/parameters**	23710/346/1512
**GOF on F^2**	1.12
**Final R index (I > 2σ(I))**	R_1 = 0.0452,<br>wR_2 = 0.1054
**R index (all data)**	R_1 = 0.0522,<br>wR_2 = 0.1089
**Peak and hole, e Å^{-3}**	2.03/-1.27
**CCDC**	2234336
